# A photoactive injectable antibacterial hydrogel to support chemo-immunotherapeutic effect of antigenic cell membrane and sorafenib by near-infrared light mediated tumor ablation

**DOI:** 10.1016/j.mtbio.2023.100609

**Published:** 2023-03-14

**Authors:** Samin Abbaszadeh, Mohammad Reza Eskandari, Vahideh Nosrati-Siahmazgi, Kiyan Musaie, Soraya Mehrabi, Ruikang Tang, Mohammad Reza Jafari, Bo Xiao, Vahid Hosseinpour Sarmadi, Fakhri Haghi, Bo Zhi Chen, Xin Dong Guo, Hélder A. Santos, Mohammad-Ali Shahbazi

**Affiliations:** a Department of Pharmacology, School of Medicine, Zanjan University of Medical Sciences, 45139-56111, Zanjan, Iran; b Department of Pharmacology and Toxicology, School of Pharmacy, Zanjan University of Medical Science, 45139-56184, Zanjan, Iran; c Department of Pharmaceutical Biomaterials, School of Pharmacy, Zanjan University of Medical Science, 45139-56184, Zanjan, Iran; d Department of Neuroscience, Faculty of Advanced Technologies in Medicine, Iran University of Medical Sciences, 14496-14535, Tehran, Iran; e Center for Biomaterials and Biopathways, Department of Chemistry, Zhejiang University, Hangzhou, Zhejiang, 310027, China; f State Key Laboratory of Silkworm Genome Biology, College of Sericulture, Textile and Biomass Sciences, Southwest University, Chongqing, 400715, China; g Cellular and Molecular Research Center, Iran University of Medical Sciences, 14496-14535, Tehran, Iran; h Department of Microbiology and Immunology, School of Medicine, Zanjan University of Medical Sciences, 45139-56111, Zanjan, Iran; i Beijing Laboratory of Biomedical Materials, College of Materials Science and Engineering, Beijing University of Chemical Technology, Beijing, China; j Department of Biomedical Engineering, University Medical Center Groningen, University of Groningen, Antonius Deusinglaan 1, 9713 AV, Groningen, Netherlands; k W.J. Kolff Institute for Biomedical Engineering and Materials Science, University of Groningen, Antonius Deusinglaan 1, 9713 AV, Groningen, the Netherlands; l Drug Research Program, Division of Pharmaceutical Chemistry and Technology, Faculty of Pharmacy, University of Helsinki, FI-00014, Helsinki, Finland

**Keywords:** Combination therapy, Photothermal therapy, Chemotherapy, Immunotherapy, Breast cancer

## Abstract

Intravenously administered nanocarriers suffer from off-target distribution, pre-targeting drug leakage, and rapid clearance, limiting their efficiency in tumor eradication. To bypass these challenges, an injectable hydrogel with time- and temperature-dependent viscosity enhancement behavior and self-healing property are reported to assist in the retention of the hydrogel in the tumor site after injection. The cancer cell membrane (CCM) and sorafenib are embedded into the hydrogel to elicit local tumor-specific immune responses and induce cancer cell apoptosis, respectively. In addition, hyaluronic acid (HA) coated Bi_2_S_3_ nanorods (BiH) are incorporated within the hydrogel to afford prolonged multi-cycle local photothermal therapy (PTT) due to the reduced diffusion of the nanorods to the surrounding tissues as a result of HA affinity toward cancer cells. The results show the promotion of immunostimulatory responses by both CCM and PTT through the release of inflammatory cytokines from immune cells, which allows localized and complete ablation of the breast tumor in an animal model by a single injection of the hydrogel. Moreover, the BiH renders strong antibacterial activity to the hydrogel, which is crucial for the clinical translation of injectable hydrogels as it minimizes the risk of infection in the post-cancer lesion formed by PTT-mediated cancer therapy.

## Graphical abstract

**Figure:**
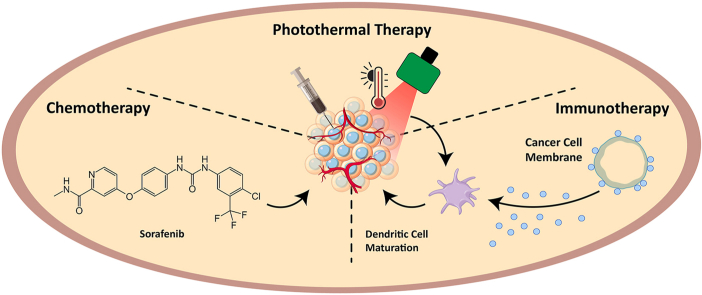

## 1 Introduction

Cancer therapy by systemic administration of drug-loaded nanoparticles (NPs) has been vastly investigated by different nanoplatforms in the past three decades to enhance drug entry to tumor tissues [1,2]. Scientists have already designed multifunctional carriers to cope with the hurdles of targeted therapy, such as the digestion of nanosystems by the reticuloendothelial system (RES) [3], enzymatic degradation [[4], [5], [6]], rapid clearance [7], protein corona formation around the nanosystems [8], and endosomal degradation after uptake into the cancer cells [9]. Nevertheless, the estimated targeting efficiency of chemotherapeutic-loaded NPs to the target tissue is still meaningfully poor, reported to be nearly 0.7% of the injected dose due to the above-mentioned physiological barriers [2,10,11]. This explains the lack of efficient products in the clinic for targeted therapy, despite huge investments over the past three decades. Contrary to popular conceptions, attachment of targeting ligands to the surface of drug-loaded NPs does not significantly alter their biodistribution in vivo because of the protein corona formation and clearance by the mononuclear phagocyte system [7,8]. In addition, monotherapy may result in drug resistance and cancer recurrence [[11], [12], [13]]. Therefore, combination therapies are superior to monotherapy and can provide promising anticancer effect with minimal adverse reactions by decreasing the amount of chemotherapeutics. This is considered as a new vision to bring better outcomes for the healthcare system from research on nanomaterials. In fact, the concept of local combination therapy by drug-loaded hydrogels [14] or solid implants [15,16] can be transformational to enhance therapeutic efficiency and overcome drug resistance with minimal systemic toxicity [17]. This can be accomplished using injectable hydrogels, which can incorporate NPs and drug molecules for multi-therapy through the preservation of therapeutic cargos in the target site [18,19].

The above discussion was the main rationale behind the conduction of the current study, in which we hypothesise a self-healable injectable hydrogel with a time-dependent viscosity enhancement feature would be able to present long-term maintenance in the tumor tissue after intratumoral injection to: (1) prolong the localization of a chemotherapeutic, a photothermal, and an immunotherapeutic agent in the cancer tissue; (2) to increase the binding and uptake of photothermal NPs with the cancer cells; (3) to directly deliver drug molecules into the cancer tissue; (4) to elicit a local immune response against cancer cells; and (5) to minimize adverse effects by preventing the non-specific distribution of drug and NPs in healthy surrounding tissues [20,21]. Accordingly, carboxylic acid groups of poly (methyl vinyl ether-*alt*-maleic anhydride) (PMVE-MA) and amines in gelatin were crosslinked by ring-opening of epoxide groups in highly water-soluble poly (ethylene glycol) diglycidyl ether (PEGDGE) to form a hydrogel that it’s stiffness and viscosity increases over time after the formation and it is adjustable by the alteration of the crosslinkerʼs concentration. The so-called PEGDGE crosslinked PMVE-MA-gelatin (PG) hydrogel was loaded with sorafenib (SFN; a multi-kinase inhibitor that promotes tumor cell apoptosis) [22], hyaluronic acid (HA) coated near-infrared (NIR)-active bismuth sulfide (Bi_2_S_3_) nanorods (called BiH), and cancer cell membrane (CCM) as an immuno-adjuvant [23,24].

Photothermal therapy (PTT) is a minimally invasive strategy for the thermal ablation of tumors with several key merits, including the capability for efficient tissue penetration, minimal systemic toxicity, and low rate of nonselective cell death on the surrounding healthy tissues due to the localized hyperthermia. Nutrient deficiency and the hypoxic nature of cancer cells make them more sensitive to heat as compared to healthy cells [25,26]. Therefore, the survival of healthy tissues during thermotherapy is expected due to the one-time usage of the platform with controlled localized heating on the tumor site [27,28]. Bi_2_S_3_ NPs were used in this study due to their high stability, high surface area, cost-effectiveness, low toxicity, and strong NIR absorbance [29]. Moreover, to enhance their retention time in the tumor tissue, they were coated with HA to produce BiH NPs. HA possesses a high affinity toward CD44 receptors of cancer cells [30,31], allowing the particles to bind to the cancer cells, remain for a longer time in the tumor tissue through less diffusion to the healthy tissues, and allow multi-intervals of PTT if needed.

In addition to direct killing of tumor cells by heat generation, PTT can also induce whole-body anti-tumor immune response through the release of tumor-specific antigens from the cancer cells, which leads to the activation of immune effector cells and the transformation of memory T lymphocytes [[32], [33], [34]]. Therefore, nanomaterial-based PTT can be further combined with immunostimulants to both ablate tumors and also perform a supplementary role in enhancing the whole body's anti-tumor immune responses by a direct effect on the release of tumor-associated antigens from the cancer tissue. That is the reason the CCM, as the source of a multi-antigenic agent, was used in this study within the hydrogel to promote anti-cancer immunities along with PTT by presenting tumor antigens to dendritic cells (DCs) to stimulate immune reactions [35,36]. This would allow the complete eradication of residual tumor cells after PTT and reduce the risk of tumor recurrence [37,38]. Moreover, the intrinsic antibacterial effect of Bi_2_S_3_ particles, which can be synergized by heat generation, would minimize serious complications related to the risk of post-treatment microbial infection in the lesion created by cancer ablation [[39], [40], [41]].

**Fig. 1:**
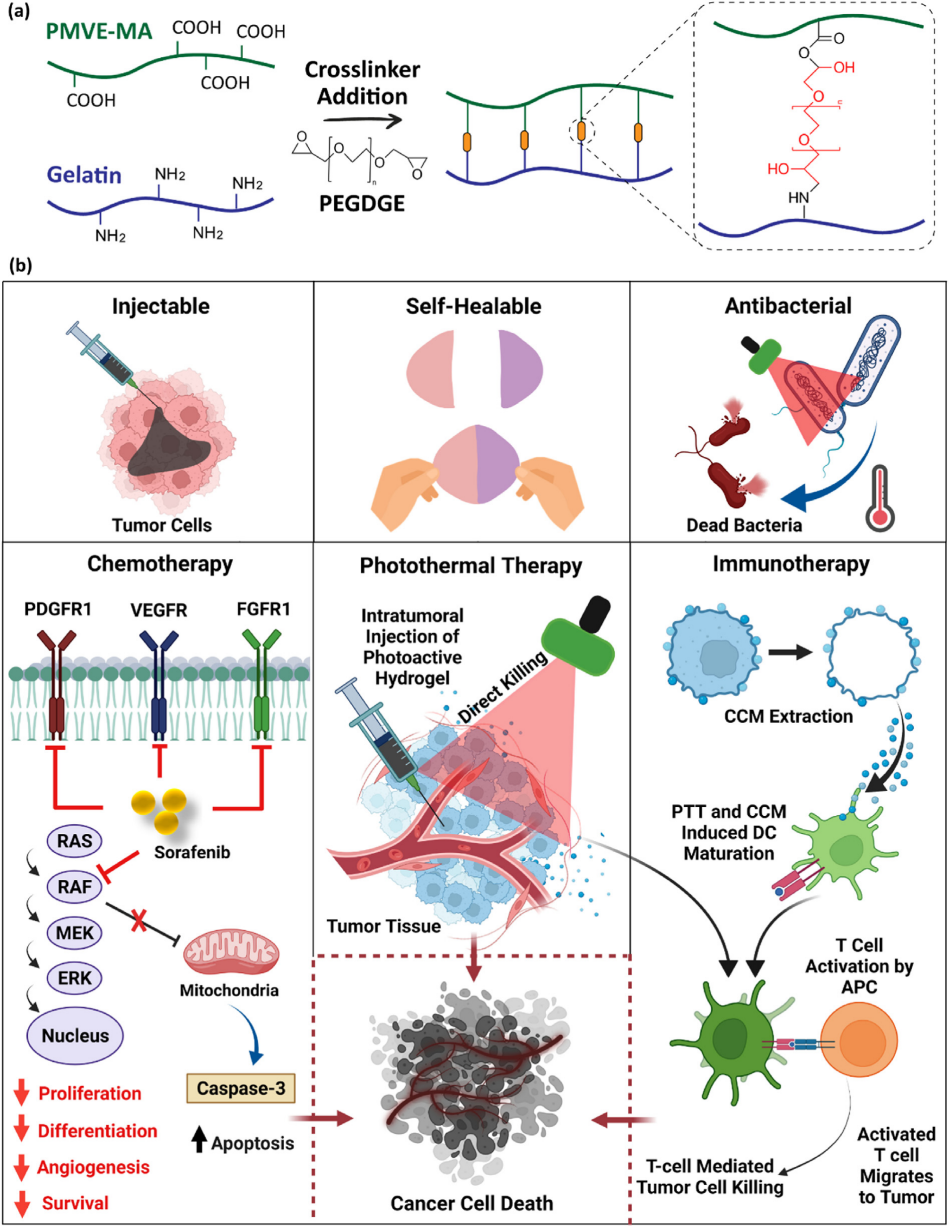
a) The chemical mechanism of hydrogel fabrication using PEGDGE crosslinker. b) schematic illustration of injectable and self-healable PG-BiH-CCM-SFN hydrogel, which presents antibacterial properties due to the intrinsic effect of the Bi_2_S_3_ and the generated heat. A synergistic mechanism of cancer chemotherapy, immunotherapy, and PTT would be able to induce potent cancer cell death. The anticancer effect of the SFN can be synergized by the PTT and delivery of tumor-associated antigens to antigen-presenting cells (APCs) through CCMs.

Overall, here we take advantage of a synergistic chemo-photothermal cancer therapy in combination with specific immunotherapy using an injectable hydrogel (Fig. 1) as a new technology to pave the road toward solving the challenges of poor targeting of intravenously injected NPs, systemic toxicity of chemotherapeutics, and non-specific immunostimulatory effect of the adjuvants for cancer therapy.

## 2 Results and discussion

### 2.1 Fabrication and characterization of nanorods and hydrogels

**Fig. 2:**
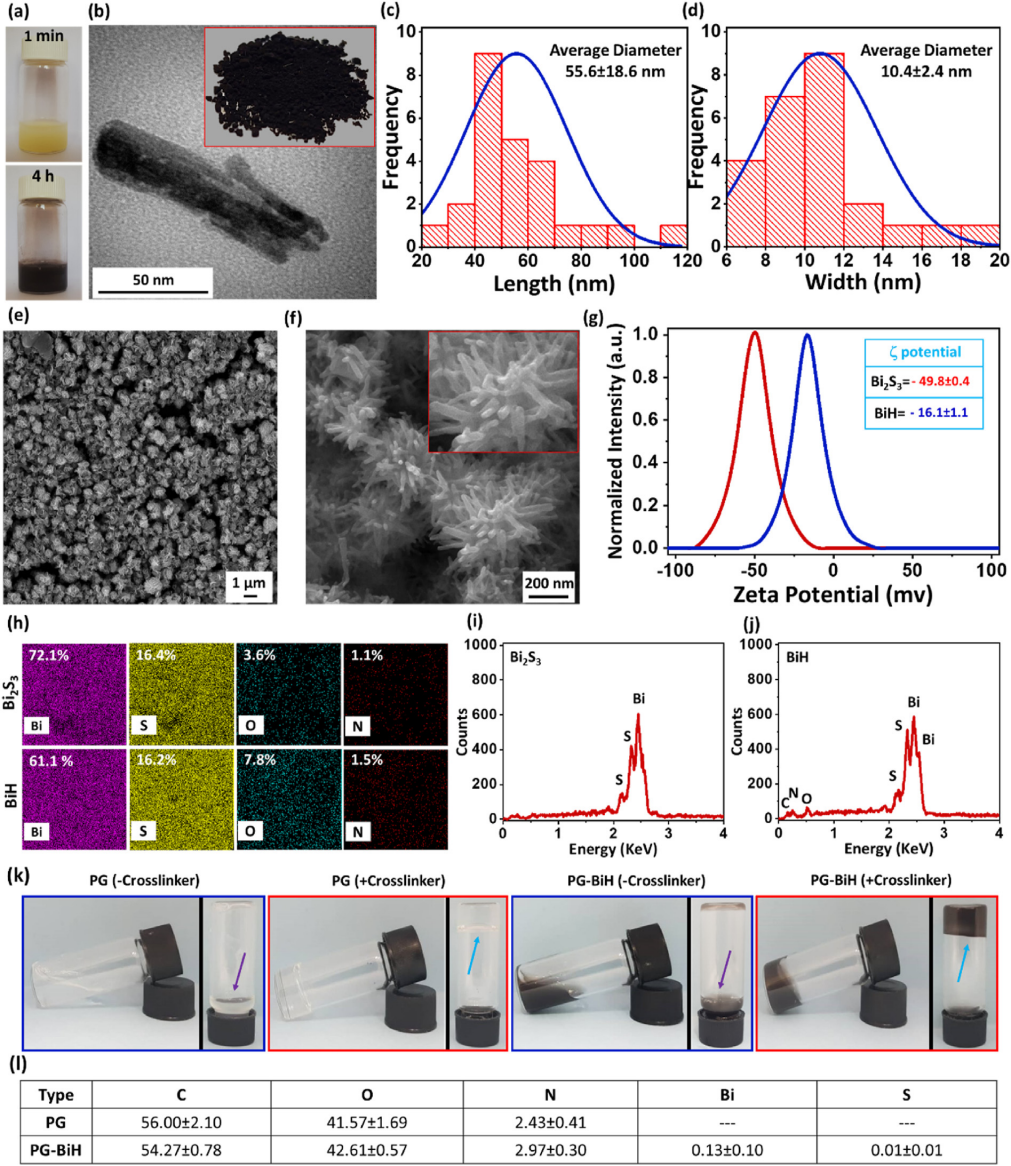
a) Photographs of Bi_2_S_3_ synthesis solution before and after particle formation. b) TEM image and the black powder of BiH nanorods. c,d) Length and width of BiH nanorods obtained from ImageJ analysis based on TEM images. e,f) FE-SEM images of BiH nanorods at different magnifications. Nanorods agglomerate to form microspheres with a diameter of 760.2 ​± ​24.3 ​nm (Fig. S2). g) ζ-potential of Bi_2_S_3_ and BiH nanorods. h-j) EDAX elemental mapping and spectra of Bi_2_S_3_ and BiH nanorods. k) Photographic images of the PG and PG-BiH with and without PEGDGE crosslinker at 55 ​°C. l) EDAX elemental analysis of PG and PG-BiH. Results are presented as mean ​± ​SD (N ​= ​3).

To demonstrate the successful fabrication of the nanorods, photographs of the synthesis media were taken at 1 ​min and 4 ​h after the starting of the Bi_2_S_3_ formation, showing the change of color from yellow to black over time (Fig. 2a). Transmission electron microscopy (TEM) imaging of Bi_2_S_3_ (Fig. S1a) and BiH nanorods (Fig. 2b) showed the rod-shaped structure of the particles before and after coating by HA. The length and width of BiH nanorods were 55.6 ​± ​18.6 ​nm and 10.4 ​± ​2.4 ​nm (Fig. 2c and d), respectively, which did not show a meaningful difference as compared to values obtained for Bi_2_S_3_ nanorods (Figs. S1b and S1c). The field emission scanning electron microscopy (FE-SEM) images supported the results obtained from TEM analysis; however, it was observed that both Bi_2_S_3_ (Figs. S1d and S1e) and BiH (Fig. 2e and f) nanorods tend to agglomerate into flower shape microspheres during the bulk drying process needed for FE-SEM imaging (Fig. S2). Moreover, the photograph of the black powder of the synthesized Bi_2_S_3_ nanorods is shown in Fig. S1f.

The surface zeta (ζ)-potential of the Bi_2_S_3_ nanorods changed from −49.8 ​± ​0.4 ​mV to −16.1 ​± ​1.1 ​mV after coating with HA (Fig. 2g). This observation demonstrates the successful coating of Bi_2_S_3_ nanorods by HA and the coverage of abundant negatively ionized sulfur groups of the particles with the carboxylic acid groups of HA. Although the less negative charge of the nanorods might enhance the chance of aggregation and less stability due to the van der Waals attractions [42], we observed higher colloidal stability of the BiH in an aqueous medium compared with Bi_2_S_3_ due to the hydrophilic nature of the HA (Figs. S3a and S3b).

Fig. 2h–j indicate the energy-dispersive X-ray spectroscopy (EDAX) elemental analysis of the Bi_2_S_3_ and BiH nanorods. The former one was composed of 72.1 ​wt-% of Bi and 16.4 ​wt-% of S, presenting a Bi:S weight ratio of 4.4, which is very close to the ideal ratio of 4.34, according to the stoichiometric ratio of the elements in Bi_2_S_3_ (81.29% for Bi and 18.71% for S) [43]. Under the experimental condition, due to the initial compounds used in Bi_2_S_3_ synthesis, a small amount of O (3.6%) and N (1.1%) were present in the obtained particles. In BiH nanorods, a higher amount of O (7.8%) and N (1.5%) was observed in the structure of the particles, supporting further the results of ζ-potential measurement and approving the presence of HA on the surface of Bi_2_S_3_ nanorods. The Brunauer-Emmett-Teller (BET) analysis of particles (Table S1) revealed the increased volume of N_2_ adsorption and surface area after HA coating due to the presence of polymeric chains on the surface of the Bi_2_S_3_ nanorods.

To evaluate the successful formation of the hydrogels, inverted tube test method was conducted 20 ​h after preparing the PG and PG-BiH hydrogels (Fig. 2k). If a vial containing a solution is tilted with no flow observation, it is defined as a gel phase. When the PG and PG-BiH were prepared without adding a crosslinker, the flow of the solution was observed at 55 ​°C, and gels were not formed. In contrast, the crosslinker led to the gel formation and no flow was observed upon inversion after incubation at 55 ​°C. The reason for performing this test at 55 ​°C was to make assure the gelation would not be due to the intrinsic gelation of gelatin at temperatures below 35–40 ​°C. The PEGDGE has two epoxide groups on two ends, which can open and react with the –COOH and –NH_2_ groups of the PMVE-MA and gelatin to form an injectable hydrogel.

The effect of PMVE-MA and gelation in the hydrogel formation was also evaluated and the results showed no hydrogel formation in the absence of PMVE-MA or gelatin 20 ​h after preparation. However, the presence of both PMVE-MA and gelatin could provide more functional –COOH and –NH_2_ groups, respectively, for the epoxides of the crosslinker and resulted in gel formation at the above-mentioned condition (Fig. S4). In addition, the well-formed hydrogel network led to the stable dispersion of BiH nanorods as shown in Fig. S5. The elemental analysis of the PG and PG-BiH hydrogels showed that in addition to the C, O, and N elements, Bi and S were present in the PG-BiH sample as compared to the PG hydrogel. In addition, the elemental mapping of PG-BiH hydrogel showed uniform distribution of Bi in the hydrogel matrix (Figure 2l and S6), confirming the well dispersity of the particles in the matrix of the hydrogel. Other characterizations, including swelling at the pH values of 7.4 and 5.8 (Fig. S7a), degradation rates (Fig. S7b), initial water content (Fig. S8a), and yield (Fig. S8b) were also performed and are discussed in the Supplementary Information. In addition, prepared nanorods and hydrogels were characterized by attenuated total reflection-Fourier transform infrared (ATR-FTIR; Fig. S9), X-ray diffraction (XRD; Fig. S10), thermogravimetric analysis (TGA), and derivative thermogravimetry (DTG; Fig. S11), and all results, as well as corresponding discussions, are present in the Supplementary Information.

### 2.2 Rheological studies of the hydrogels

**Fig. 3:**
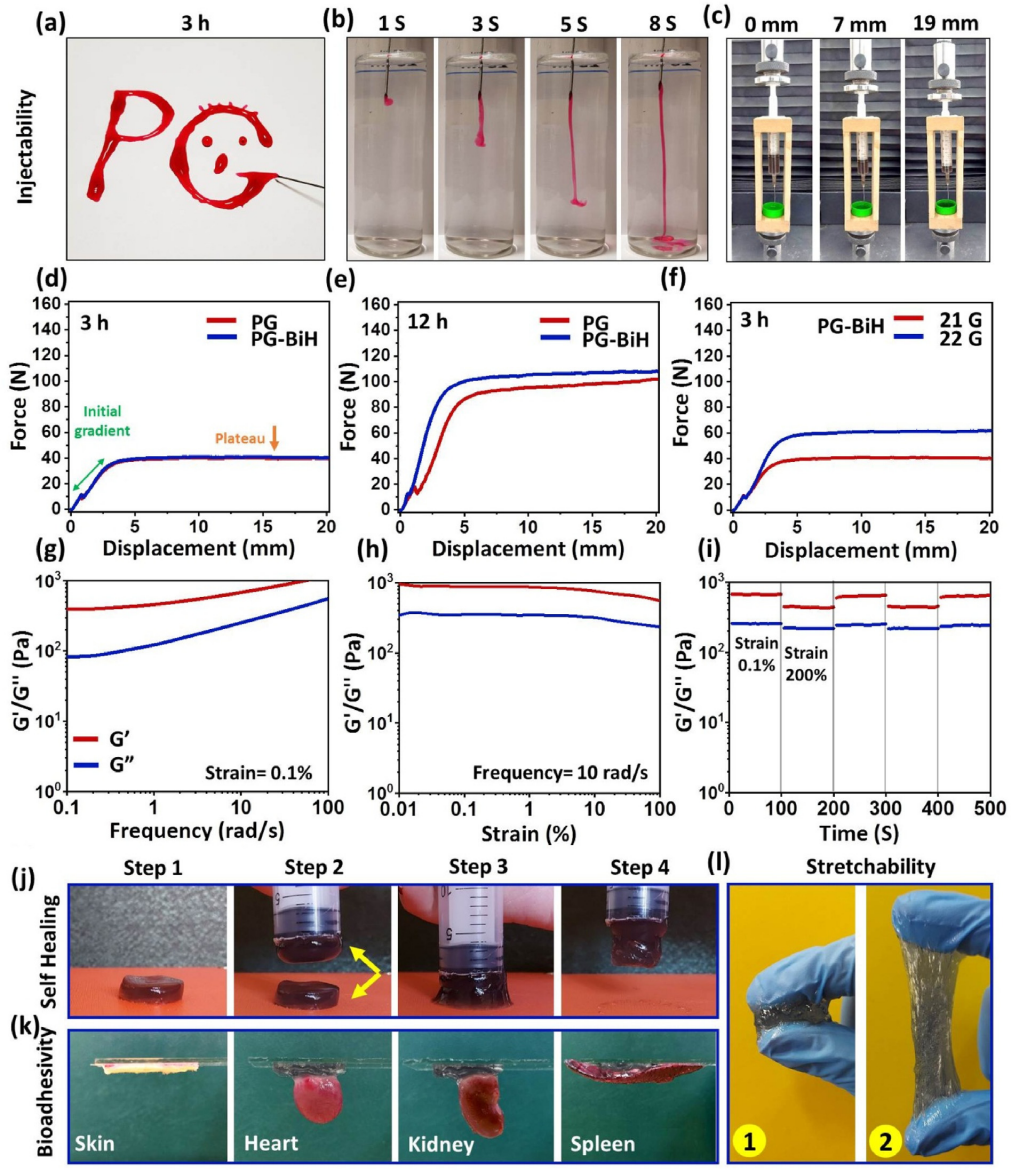
a) Photo of the hydrogel extruded using a syringe to write “PG”. b) The injectability of the hydrogel over 8 ​s. c) Setup of the mechanical tester for injectability assay. d,e) Injection force-displacement curves for PG and PG-BiH hydrogels injected from a 10 ​ml syringe using a 21 ​G needle at 3 and 12 ​h after the preparation of the hydrogels. f) Comparison of needles with different Gs to evaluate the force needed for the injection of PG-BiH from a 10 ​ml syringe 3 ​h after preparation. g-i) Rheological properties of the PG-BiH hydrogel 10 days after hydrogel formation. The graphs of 24 ​h post-formation of the hydrogel are presented in Fig. S16. J) Self-healing, K) bioadhesivity, and l) stretchability of PG-BiH hydrogel 10 days after preparation.

Injectability and the force required for the injection of the hydrogel are important parameters to take into account for the development of injectable hydrogels. These criteria are directly related to the rheological properties of the hydrogel. To test the injectability of the hydrogels, the letters “PG” were successfully written using the PG hydrogel 3 ​h after preparation (Fig. 3a). In addition, the PG hydrogel could be ejected through a 21-gauge (G) needle without clogging overtime (Fig. 3b and Video 1).

Viscosity is the primary rheologic feature used to characterize injectable hydrogels. The viscosities of PG (Fig. S12a) and PG-BiH (Fig. S12b) hydrogels decrease with increasing shear rates at 3 ​h, 24 ​h, and 10 days after preparation, indicating the shear-thinning behavior of both hydrogels [44], which is one of the desired features of the injectable hydrogels. Notably, when the BiH nanorods were incorporated into the hydrogel, the viscosity of PG-BiH hydrogel increased, showing that the strength of PG hydrogel could be increased by the addition of BiH nanorods. In addition, the viscosity of both hydrogels was increased time-dependently, indicating time-dependent chemical crosslinking of the polymers in the hydrogel. This behavior allows the hydrogel to be injected locally into the tumor site after preparation and overtime increment of the hydrogel's viscosity can assist the retention of the hydrogels in the tumor site after injection.

Injectability was also evaluated by monitoring the force needed for the injection of the hydrogel versus displacement under a constant speed [45]. The injection force to eject both PG and PG-BiH hydrogels from 10-ml syringes with different needle Gs was tested using a set-up shown in Fig. 3c and S13a. The obtained force-displacement curve demonstrates an initial upward gradient associated with the force needed to overcome the friction between the plunger and the barrel of the syringe at the beginning of the injection. Then the hydrogel starts to exit from the needle and the needed force for the extrusion of the hydrogel reaches a plateau [46]. We observed that when hydrogels were prepared and stored at room temperature, the force required for injection of the PG-BiH hydrogel was about 41 ​N using a 21 ​G syringe and 3 ​h after preparation, which did not show a significant difference as compared to PG hydrogel (Fig. 3d). However, 12 ​h and 24 ​h after preparation of hydrogels, the maximum needed force was meaningfully increased and showed even higher values for PG-BiH as compared to the PG (Fig. 3e and S13b). This data shows that the hydrogel should be injected a maximum of 3 ​h after preparation and it will become more viscous post-injection, which is desirable to keep the hydrogel and its payloads in the site of interest for a longer period. Moreover, as expected, reducing the needle diameter from 21 ​G to 22 ​G increased the force required for the injection of PG and PG-BiH hydrogels at all-time points (Fig. 3f and S14).

Furthermore, when the hydrogels were stored at 37 ​°C for 3 ​h post-preparation, the results showed the increment of force required for the ejection of both hydrogels from both 21 ​G and 22 ​G needles compared to storing at room temperature (Fig. S15). In fact, the rate of crosslinking reactions can be increased at higher temperatures [47], leading to the enhanced stiffness of the hydrogel.

The viscoelastic properties of the PG and PG-BiH hydrogels were tested 24 ​h and 10 days after preparation. A hydrogel is generally recognized by a bigger G′ value than G″, which means the material has a more elastic nature than vicious nature. Frequency sweep measurements at both above-mentioned time points showed that PG-BiH (Fig. 3g and S16a) and PG (Figs. S17a and S17b) possess higher G′ values than G″ values throughout the frequency sweep of 0.1–100 ​rad ​s^−1^ under the constant strain of 0.1%, which indicates gel-like behavior. Fig. 3h and S16b show that the moduli of PG-BiH were independent of strain amplitude 24 ​h and 10 days after the hydrogel formation, confirming linear viscoelastic behavior at the strain range of 0.01–100%. The stable viscoelastic behavior at different strains approves the synthesis of a gel-like hydrogel that can provide the required local retention of the hydrogel at the injection site, which is necessary for targeted delivery applications for tumor therapy. The moduli of PG were independent of strain amplitude at low strain ranging from 0.01 to 2% at 24 ​h after preparation and 0.01–100% after 10 days of hydrogel preparation (Figs. S17c and S17d). Moreover, G′ and G″ values on day 10 after hydrogel formation were higher than those of 24 ​h in the whole investigated frequency range (0.1–100 ​rad ​s^−1^) and strain scanning at a fixed frequency. This observation can be attributed to the time-dependent enhanced chemical interaction in the cross-linked hydrogel, which contributes to the stiffness of the hydrogel over time and increase in G′ and G″ values.

Furthermore, the self-recovering ability of hydrogels was assessed by alternate-step strain measurement. For this purpose, continuous changing of strain between 0.1% and 200% at the frequency of 10 ​rad ​s^−1^ was performed to assess the strain-induced damage and self-healing ability of PG and PG-BiH hydrogels. The results showed that G′ and G″ remained constant after 5 cycles of breaking down and reforming, supporting that PG (Figs. S17e and S17f) and PG-BiH (Fig. 3i and S16c) hydrogels had desirable self-healing properties attributed to the abundantly available hydrogen bonds of gelatin and PMVE-MA, which provides physical crosslinks [48]. Under a high strain (200%), the G′ value of both hydrogels dramatically decreased, demonstrating the collapse of the hydrogel structure. However, G″ was rapidly restored to the initial value at a low strain of 1%, showing that the network of the hydrogel recovered rapidly.

To further evaluate the self-healing ability of the PG and PG-BiH hydrogels, we monitored the healing process with the naked eye. When two disk-shaped PG-BiH (Fig. 3j) and PG (Fig. S18a) hydrogels were closely contacted along with each other for 3 ​min at room temperature, they healed into a single disk-shaped hydrogel without any external intervention. This self-healing phenomenon enables the hydrogel to repair after getting damaged while passing through the syringe and later become a single bulk hydrogel after injection to the site of interest and prolong the remaining time in the target site. The PG-BiH and PG hydrogels also represented bioadhesivity to the surface of different organs, including skin, heart, kidney, and spleen (Fig. 3k and S18b) and high stretchability (Fig. 3l and S18c), which are essential factors for the practical bio-applications of hydrogels. In general, the rheological studies demonstrated that the incorporation of BiH nanorods in the hydrogel increases the viscosity and as a result improves the mechanical properties of the hydrogels compared to PG hydrogel without compromising the shear-thinning properties and injectability.

### 2.3 Evaluation of the photothermal effect

#### 2.3.1 Photothermal effect of the nanorods and hydrogels in vitro

**Fig. 4:**
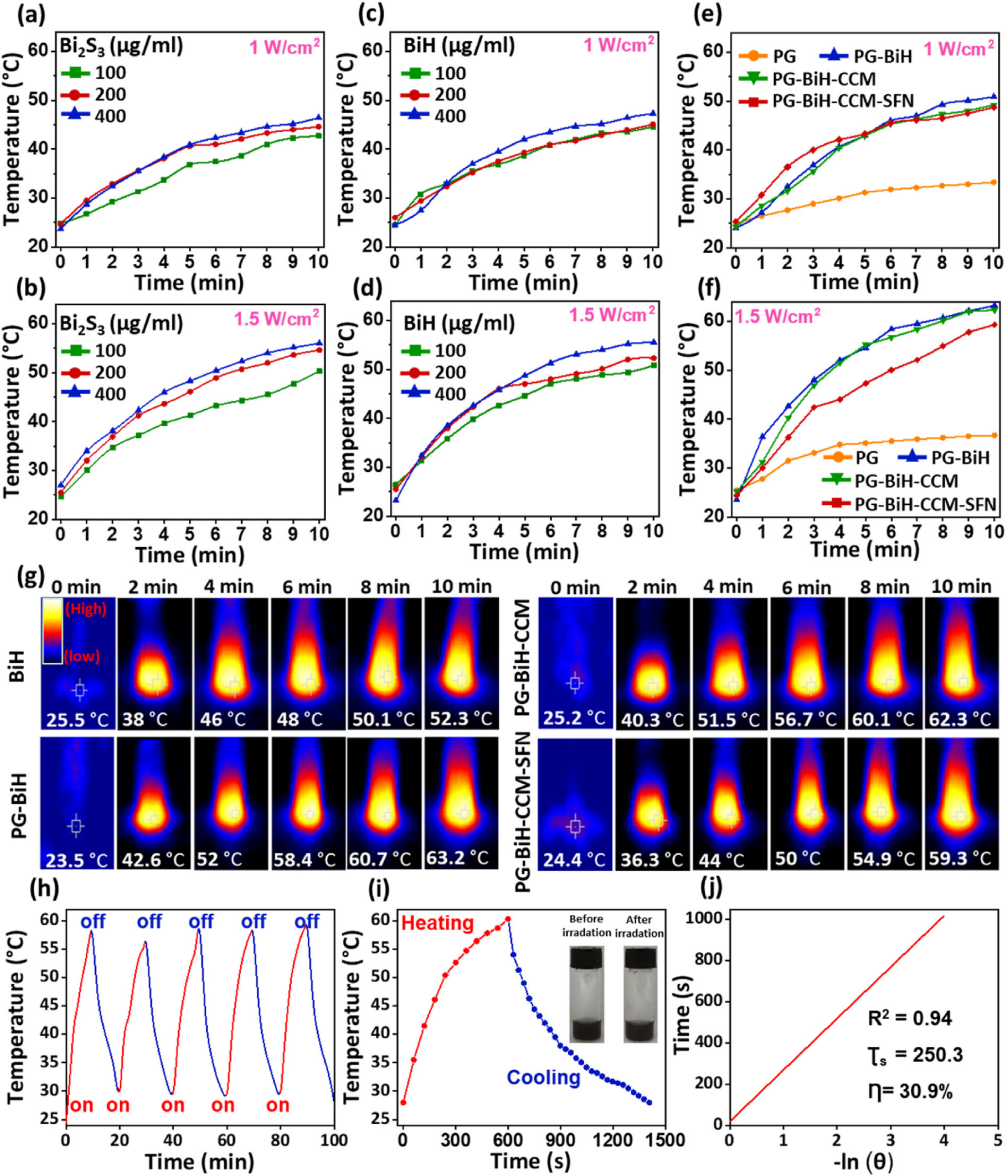
Temperature curves of the aqueous dispersions of a,b) Bi_2_S_3_ and c,d) BiH nanorods at various concentrations and laser powers. e,f) Time-dependent temperature enhancement of PG, PG-BiH, PG-BiH-CCM, and PG-BiH-CCM-SFN hydrogels (all with 200 ​μg ​ml^−1^ of BiH), under NIR laser irradiation with 1, and 1.5 ​W ​cm^−2^ power for 10 ​min. g) Infrared thermal photographs of different formulations captured during irradiation of an 808 ​nm laser for 10 ​min (1.5 ​W ​cm^−2^). h) Temperature curve of PG-BiH-CCM-SFN hydrogel for five ON/OFF cycles under an 808 ​nm laser irradiation. i) Temperature elevation of PG-BiH-CCM-SFN hydrogel exposed to the NIR laser (808 ​nm, 1.5 ​W ​cm^−2^). Irradiation was continued for 10 ​min, and then the laser was turned off to monitor the cooling profile of the sample. j) Linear time data versus -ln θ obtained from the cooling period of Fig. 4i.

The UV–vis spectra of both the Bi_2_S_3_ and BiH nanorods with different concentrations indicated an absorption extending to the NIR region that suggested their perfect potential to be used as a photothermal agent in cancer treatment (Figs. S19a and S19b). Despite the difference between the UV-absorption of Bi_2_S_3_ and BiH nanorods at wavelengths less than 770 ​nm, they showed the same behavior at 808 ​nm, indicating that the absorption at the mentioned wavelength is not affected by the HA coating (Fig. S19c). To evaluate the NIR-induced photothermal effect of Bi_2_S_3_ and BiH nanorods, various concentrations (100, 200, and 400 ​μg ​ml^−1^) of nanorods (Fig. S20) were irradiated by a NIR laser at 808 ​nm (1 and 1.5 ​W ​cm^−2^) over 10 ​min. The results showed a time and concentration-dependent increase in the temperature of solutions containing different concentrations of Bi_2_S_3_ nanorods (Fig. 4a and b). The temperature of different concentrations (100, 200, and 400 ​μg ​ml^−1^) of BiH nanorods increased dramatically to 44.6 ​°C, 45 ​°C, and 47.3 ​°C, respectively, with a power density of 1 ​W ​cm^−2^ (Fig. 4c), and to 50.8 ​°C, 52.3 ​°C, and 55.5 ​°C with a power density of 1.5 ​W ​cm^−2^ (Fig. 4d), which is sufficient to kill cancer cells. Moreover, the UV-absorption of Bi_2_S_3_ and BiH nanorods did not differ before and after laser irradiation (10 ​min, 1.5 ​W ​cm^−2^) at 808 ​nm (Fig. S19d).

Typically, to achieve a relatively harsh environment for efficient ablation of tumors, a temperature of above 45 ​°C is required, which can lead to protein aggregation and denaturation, cell lysis, and apoptosis of cancerous cells [29,49,50]. Therefore, 200 ​μg ​ml^−1^ of BiH nanorods and an optimum power density of 1.5 ​W ​cm^−2^ were chosen in the other experiments.

The photothermal effect of different formulations (PG, PG-BiH, PG-BiH-CCM, and PG-BiH-CCM-SFN) was assessed in 10 ​min upon NIR light irradiation (808 ​nm; 1 and 1.5 ​W ​cm^−2^) (Fig. 4e and f). The temperature increased in all formulations containing 200 ​μg ​ml^−1^ of BiH nanorods to above 59 ​°C at the highest power (1.5 ​W ​cm^−2^). However, the NIR exposure did not make much change to the temperature of PG. The above results indicated the desirable light-heat conversion ability of the BiH nanorods. The photothermal conversion was higher for hydrogels containing BiH nanorods under NIR laser irradiation as compared to BiH nanorods solutions under the same condition. This is due to the poor dispersibility of nanorods in water, which reduces their photothermal conversion performance. The incorporation of nanorods into hydrogels could facilitate the dispersion and stabilization of nanorods and subsequently result in better heat generation. The thermal images of BiH nanorods and hydrogels containing BiH are shown in Fig. 4g.

The photothermal stability of the hydrogel is a crucial factor that can supply constant heat generation for multiple photothermal therapies at different intervals after a single injection. To evaluate the photothermal stability of the final hydrogel with all components the temperature was measured in five different cycles and demonstrated constant heat generation in all cycles (Fig. 4h). To assess the photothermal conversion efficiency (ƞ) of the BiH nanorods, the PG-BiH-CCM-SFN hydrogel (1 ​ml) was exposed to a laser (808 ​nm, 1.5 ​W ​cm^−2^) for 10 ​min and then cooled down naturally, as shown in Fig. 4i. Moreover, the appearance and color of the final hydrogel did not change after irradiation with an 808 ​nm laser for 10 ​min (Fig. 4i). ƞ of the BiH nanorods was calculated according to Equation (6) presented in the Material and methods Section of the Supplementary Information. The η value was determined to be 30.9% (Fig. 4j), which is high enough to reduce the dose of PTT agents during the process of cancer treatment.

#### 2.3.2 In vivo photothermal effect

The photothermal effect was also assessed on mice after subcutaneous (*s.c.*) injection of 100 ​μl phosphate-buffered saline (PBS), PG, BiH, and PG-BiH, followed by NIR irradiation (1.5 ​W ​cm^−2^, 5 ​min) 2 ​h after injection. As shown in Fig. S21a, the local temperature of the injected area with BiH and PG-BiH hydrogel rapidly enhanced to 56.1 ​°C and 58.7 ​°C, respectively, whereas PBS and PG showed only a little increase under the same irradiation condition. Moreover, we evaluated the PTT effect of PG-BiH hydrogel two days after injection and the temperature rose the same, which confirms the retention of the hydrogel at the injected site as well as photothermal stability of the hydrogel.

In addition, PG-BiH hydrogel (100 ​μl) was injected through the *s.c.* route to mice and then subjected to the 808 ​nm laser exposure with different power densities, followed by monitoring the temperature change using an infrared thermal camera (Figs. S21b and S21c). We found that the local temperature of the injected area with hydrogel under NIR laser irradiation at 0.65, 1, and 1.5 ​W ​cm^−2^ rapidly increased to ≈48.6 ​°C, 61.6 ​°C, and 61.5 ​°C, respectively within 8 ​min, which was high enough to kill the tumor cells in vivo.

### 2.4 Cellular viability and in vitro cytotoxicity studies

**Fig. 5:**
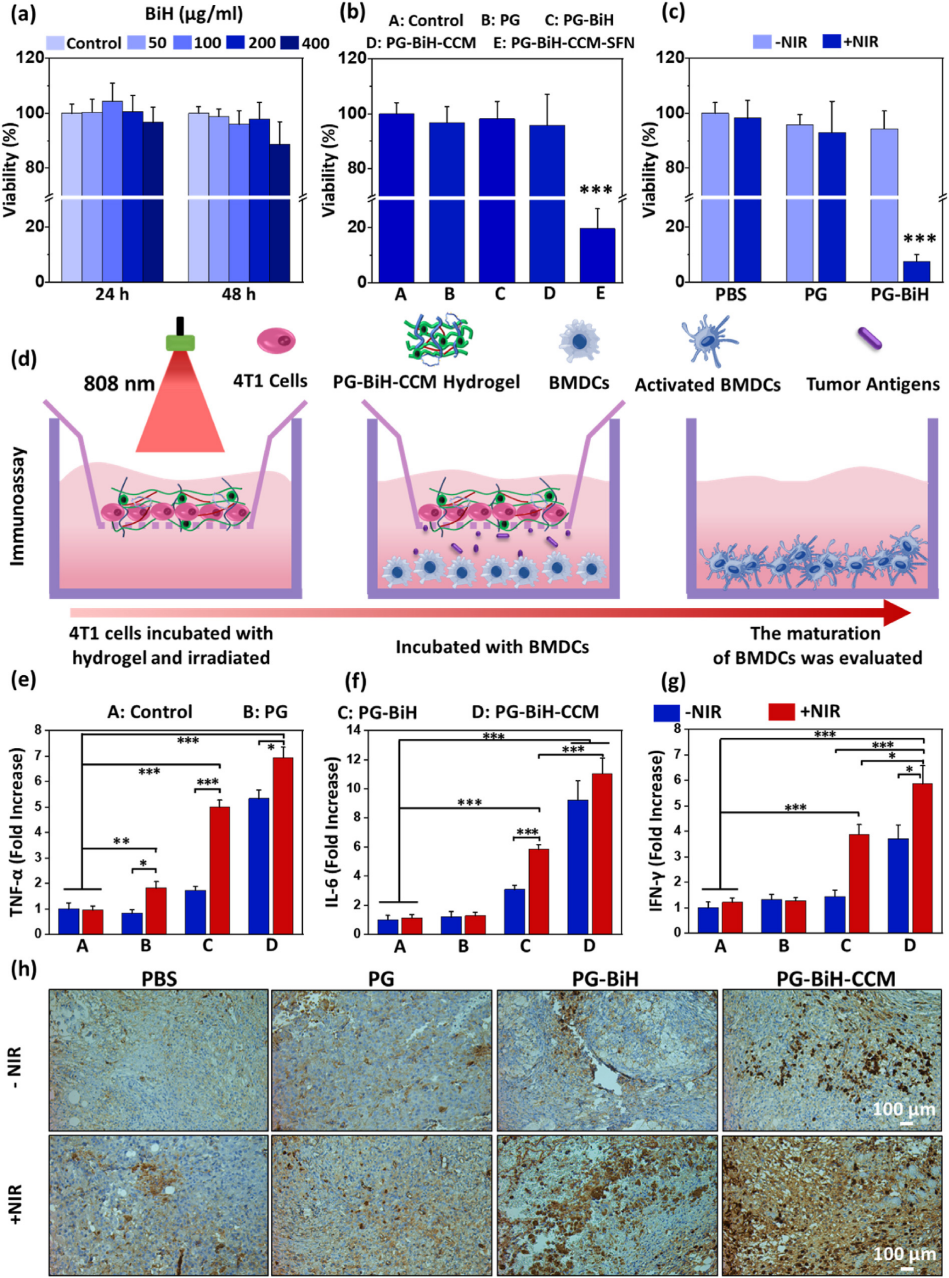
a) The effect of BiH (50, 100, 200, and 400 ​μg ​ml^−1^) on the viability of 4T1 cells as compared to the control group at 24 and 48 ​h after treatment. b) The viability of 4T1 cancer cells after treatment with different hydrogels at 37 ​°C for 24 ​h. c) In vitro photothermal killing effect of PG and PG-BiH hydrogels as a function of NIR laser irradiation (808 ​nm, 1.0 ​W ​cm^−2^ for 10 ​min). Data are presented as mean ​± ​SD (N ​= ​4), (∗∗∗p ​< ​0.001). d) Schematic illustration of the transwell system to study the immuno-stimulatory effect of the hydrogels. Fold increase in the sections of e) TNF-α, f) IL-6, and g) IFN-γ by the BMDCs in the transwell study (N ​= ​3). Data are presented as mean ​± ​SD (N ​= ​4), (∗p ​< ​0.05, ∗∗p ​< ​0.01, and ∗∗∗p ​< ​0.001). h) Representative IHC photographs of TNF-α expression in the tumor tissues of mice treated with different formulations. The staining was conducted 15 days after the injection of the hydrogels to the animals.

The in vitro cytotoxicity of nanorods and hydrogels was evaluated on the 4T1 breast cancer cell line, as a proof of concept. As shown in Fig. 5a and S22, no obvious cytotoxicity effect was observed for different concentrations of Bi_2_S_3_ and BiH nanorods as a function of concentration (up to 400 ​μg ​ml^−1^) at two different time points of 24 and 48 ​h. As for the hydrogels (Fig. 5b), the cell viability was found to be over 95% in all groups except for the PG-BiH-CCM-SFN hydrogel, which killed more than 80% of the cancer cells in vitro. This is due to the presence of SFN as a chemotherapeutic agent with high toxicity within the hydrogel network. SFN is a multikinase inhibitor that suppresses Raf serine/threonine kinases and induces cell apoptosis through a caspase-dependent mechanism [51]. This observation confirms the preserved potent anticancer effect of the SFN after incorporation into the hydrogel backbone.

The anticancer properties of PG and PG-BiH loaded hydrogels on the 4T1 cells after exposure to 808 ​nm laser were also evaluated to understand the killing effect of NIR-mediated heat generation on cancer cells. Without laser irradiation, none of the samples could induce anticancer effect due to the lack of heat generation (Fig. 5c). In addition, while PBS and PG treated cells were alive after NIR light exposure, PG-BiH ​+ ​NIR killed more than 92% of 4T1 cells. The result is in line with previous studies that have reported induced cancer cell necroptosis and apoptosis when the temperature touches above 46 ​°C [49].

Since hemocompatibility is one of the needed characteristics for the in vivo translation of biomaterials [52], the hemolysis effect of hydrogels at different concentrations and time points was also evaluated on human red blood cells (RBCs). According to ISO standards, a hemolysis index of less than 5% is considered safe for a substance [53]. As shown in Fig. S23a, the percentages of non-hemolysed RBCs were above 95% at all tested concentrations of hydrogels and different time points. The only exception was the highest examined concentration of hydrogels, which could induce 7–9% of hemolysis within 24 ​h. Fig. S23b exhibits the optical photographs of the supernatants after the centrifugation of the RBCs treated with different samples. This study suggests that the prepared hydrogels are hemocompatible, making them appropriate candidates for biomedical applications.

### 2.5 In vitro and in vivo evaluation of tumor immunity

In vitro transwell assay was employed to observe the immuno-stimulatory effect of the hydrogels and PTT treatment on dendritic cells in the presence of cancer cells (Fig. 5d). As compared to the control, PG hydrogel displayed no obvious enhancement of pro-inflammatory cytokines (tumor necrosis factor-alpha (TNF-α), interleukin-6 (IL-6), and interferon-gamma (IFN-γ)) in the culturing media of the bone marrow-derived dendritic cells (BMDCs) for both ​+ ​NIR and –NIR groups (Fig. 5e–g). The only exception was the slight enhancement of TNF-α by the PG when the NIR light was irradiated to the sample. This can be presumably due to the partial immuno-stimulatory effect of PMVE-MA released from the hydrogel [54]. In the PG-BiH ​+ ​NIR group, the secretions of all the above-mentioned cytokines were substantially increased by the BMDCs. This is attributed to the release of tumor antigens, heat-shock protein, and cell debris from cancer cells by the induced PTT effect of the BiH particles [55]. Without laser irradiation, PG-BiH treated cells could just slightly promote the release TNF-α and IL-6, which is assumed to be associated with the limited release of cancer cell antigens, the partial immuno-stimulatory effect of PMVE-MA, and the effect of BiH particles on immune cells. Impressively, the PG-BiH-CCM hydrogel could elicit remarkable pro-inflammatory responses in both ​+ ​NIR and –NIR groups with higher values for the former one. This is due to the presence of CCM and its direct effect on the BMDCs even in the absence of NIR light. However, the NIR-mediated killing of the cancer cells and the release of antigens from the apical part of the transwell plate could synergize the effect of CCM.

The maturation of DCs cultured in the basal part of the transwell was also studied by monitoring the expression of co-stimulatory CD80 and CD86 proteins on the surface of the cells at the end of the study. The results were in line with the observed cytokine secretion data in which the combination of CCM and NIR light could induce the highest rate of CD80 and CD86 expression by the BMDCs (Fig. S24).

To support the in vitro data, the expressions of TNF-α and CD86 were monitored in vivo by the immunohistochemistry (IHC) tumor staining of cancer-bearing mice in different groups 15 days after the start of the therapy (Fig. 5h, S25, and S26; methodology is explained in the SI file). The expressions of both factors were negligible in the control and PG hydrogel receiving groups. In addition, their expressions were remarkably higher in the tumor tissue of animals receiving PG-BiH-CCM ​+ ​NIR as compared to the PG-BiH ​± ​NIR as well as PG-BiH-CCM without NIR irradiation. TNF-α and CD86 are key mediators of cellular immunity in the tumor microenvironment and their higher expressions indicate the enhanced immune response due to the combined immuno-stimulatory effects of CCM and PTT.

Taken together, these data indicate that the CCM and BiH-mediated heat generation by NIR light can work together to promote antitumor responses and enhance the efficacy of cancer treatment. It is already known that T-cells are responsible for anticancer immunity and their activation is through their crosstalk with antigen presenting cells [[56], [57], [58], [59]]. Therefore, while DCs do not directly destroy cancer cells, they have a key role on the initiation of systemic immunity against cancer by presenting antigens to the T-cells [60,61]. Therefore, the main goal of our work was mainly to develop a hydrogel with the CCM to study whether the presence of CCM in the hydrogel can activate DCs or not. Here, the observed promising results of the DCsʼ activation in the in vitro assays demonstrate that our hydrogel can contribute in the initiation of anticancer immunity.

### 2.6 Antibacterial activity of BiH nanorods and hydrogel

**Fig. 6:**
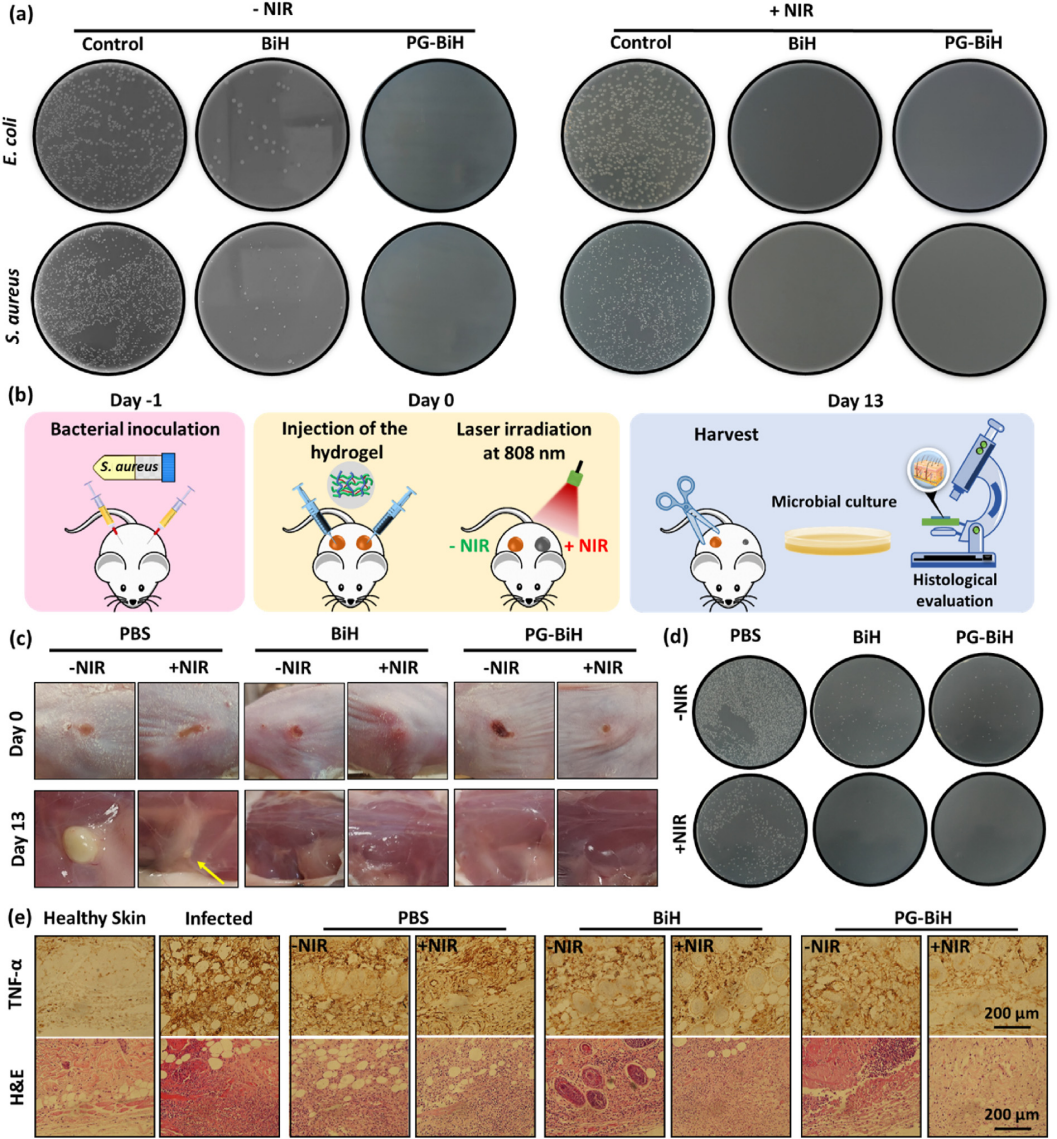
a) Representative images of bacterial colonies for *E. coli* and *S. aureus* suspensions on agar plates with and without NIR irradiation following 24 ​h of incubation. b) Schematic illustration of anti-infective treatment. c) Photographs of S. aureus-infected area after various treatments on days 1 and 13. d) The corresponding photographs of agar plates of *S. aureus* treated with PBS, BiH, and PG-BiH with and without NIR irradiation. e) H&E staining and immunohistochemical study of TNF-α expression in infected tissue of different groups.

During cancer treatment, microbial infections can disrupt the therapeutic effectiveness and lead to serious complications due to the suppressed systemic immunity of cancer patients [39]. Therefore, the antibacterial effect of materials used for cancer treatment can add more value to their therapeutic effects, especially when the cancer treatment leads to the creation of local lesion. In Fig. 6a, the images of agar plates in the colony counting assay show the antibacterial activity of BiH nanorods and PG-BiH hydrogel against *Escherichia coli* (*E. coli*) and *Staphylococcus aureus* (*S. aureus*) bacteria. The results demonstrated that BiH nanorods and PG-BiH hydrogel could destroy most of the bacteria even without NIR laser irradiation, which was associated with the strong antibacterial activity of BiH and maleic acid in the structure of the PMVE-MA copolymers [62]. In fact, BiH nanorods showed antibacterial activity via the destruction of the bacterial membrane and induction of reactive oxygen species (ROS) generation in bacteria [40]. Moreover, with the assistance of NIR, its bactericidal potency against *E. coli* and *S. aureus* could rise to 100%. Since BiH nanorods possess high photothermal conversion efficiency, the light energy can convert into heat by NIR light irradiation, consequently leading to the collapse of the bacterial structure and its death [63].The results suggest the bacteria-killing effect of photoactive NPs, which is in line with the results of other studies. For example, Feng et al. showed the good antibacterial activity of 2D reduced graphene oxide supported Au nanostar nanocomposites under NIR irradiation for methicillin-resistant *S. aureus* (MRSA) bacteria due to the synergistic antibacterial activity [64]. Moreover, some studies have combined the intrinsic antibacterial activity of NPs with photothermal therapy to decrease the dose of nanoparticles as well as NIR exposure time [65].

The in vivo antibacterial activity of BiH nanorods and PG-BiH hydrogel was further tested in an abscess animal model of MRSA infection (Fig. 6b).

After treatment, the mice in BiH and PG-BiH groups showed a remarkable decrease in the size of the abscess, and no evident inflammation and abscess was observed in NIR irradiated groups. However, obvious infection and swelling of the skin containing bacteria were still observed in the PBS-treated groups with or without laser irradiation (Fig. 6c). The infected tissues were also homogenized for quantifying the number of bacteria. As shown in Fig. 6d the results of colony counting further confirmed the good antibacterial activity of BiH nanorods and in NIR-treated BiH and PG-BiH groups, almost all of the bacteria were eliminated, presenting a relatively higher antibacterial property, which was consistent with the in vitro results. Moreover, Hematoxylin-eosin (H&E) staining and the immunohistochemical study of TNF-α expression in infected tissue were performed on day 13. As shown in Fig. 6e and S27, the blank group showed normal histology. In the infected group and PBS-treated groups with and without NIR irradiation, TNF-α expression is greatly increased. As well, a large number of neutrophils was revealed in these groups, indicating a significant infection by *S. aureus*. Although the accumulation of immune cells in the BiH treated groups was slightly decreased, TNF-α expression was still observed. Because the inflammation caused by the presence of bacteria lasts for a while. The accumulation of immune cells and inflammation in the PG-BiH treated group was slightly less than that in the BiH group without NIR laser irradiation due to the loading of BiH nanorods in the hydrogel. The lowest TNF-α expression was observed in the PG-BiH group after NIR laser irradiation, which may be due in part to the anti-inflammatory properties of gelatin.

### 2.7 In vivo toxicity of the hydrogels

**Fig. 7:**
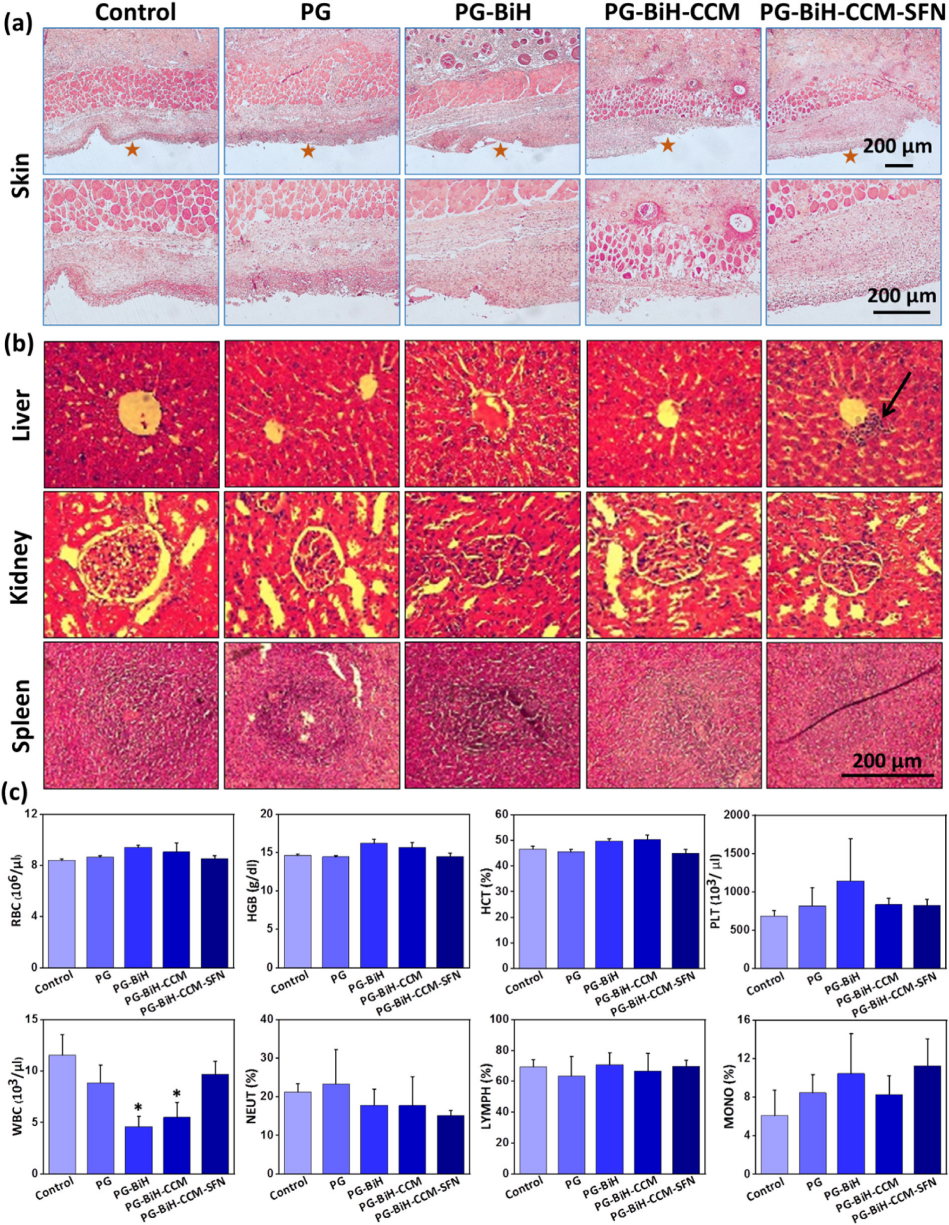
In vivo toxicity assessment of hydrogels. H&E stained tissue slices. a) Skin with two different magnifications (star symbols represent the site of injection of hydrogel) and b) liver, kidney, and spleen of control and hydrogel treated mice after 14 days. The black arrow shows the accumulation of inflammatory cells. c) Hematological factors of animals for treated groups. Results are presented as mean ​± ​SD (N ​= ​4). The statistical analysis was performed using One-way ANOVA (∗denotes significant differences with a significance level of p ​< ​0.05), by comparing each group with the corresponding control group.

In vivo toxicity of hydrogels was further examined after 14 days. As shown in Fig. 7a, no abnormality was observed in the histopathological analysis of the skin. However, PG-BiH-CCM and PG-BiH-CCM-SFN treated groups showed blood vessel congestion results in activation of vascular endothelium to promote inflammation and mild infiltration by neutrophils. Furthermore, the main organs, including the liver, kidney, and spleen in all treated groups, exhibited an integrated tissue structure after 14 days and no obvious histopathological changes, such as necrosis (Fig. 7b and S28). However, histologic findings of the liver biopsy of the PG-BiH-CCM-SFN treated group showed mild portal inflammation with immune cell infiltration. In addition, hematological and serum biochemical parameters are measured to evaluate the normal function of different organs (Fig. 7c and S29). Except for the number of white blood cells (WBCs) in the PG-BiH and PG-BiH-CCM treated groups and alkaline phosphatase (ALP) of the PG-BiH treated group, no significant change was observed in blood level of RBCs, hemoglobin (HGB), hematocrit (HCT), mean corpuscular volume (MCV), platelet (PLT), mean platelet volume (MPV), neutrophils (NEUT), lymphocytes (LYMPH), and monocytes (MONO), when compared to the control group. In addition, blood levels of total protein (TP), calcium (Ca), phosphor (Ph), blood urea nitrogen (BUN), creatinine (CREA), BUN/CREA ratio, and lactate dehydrogenase (LDH), showed no significant toxicity of hydrogels. Overall, it can be concluded that the prepared hydrogels are safe to use as tumor drug delivery agents.

### 2.8 In vivo anti-tumor effect

**Fig. 8:**
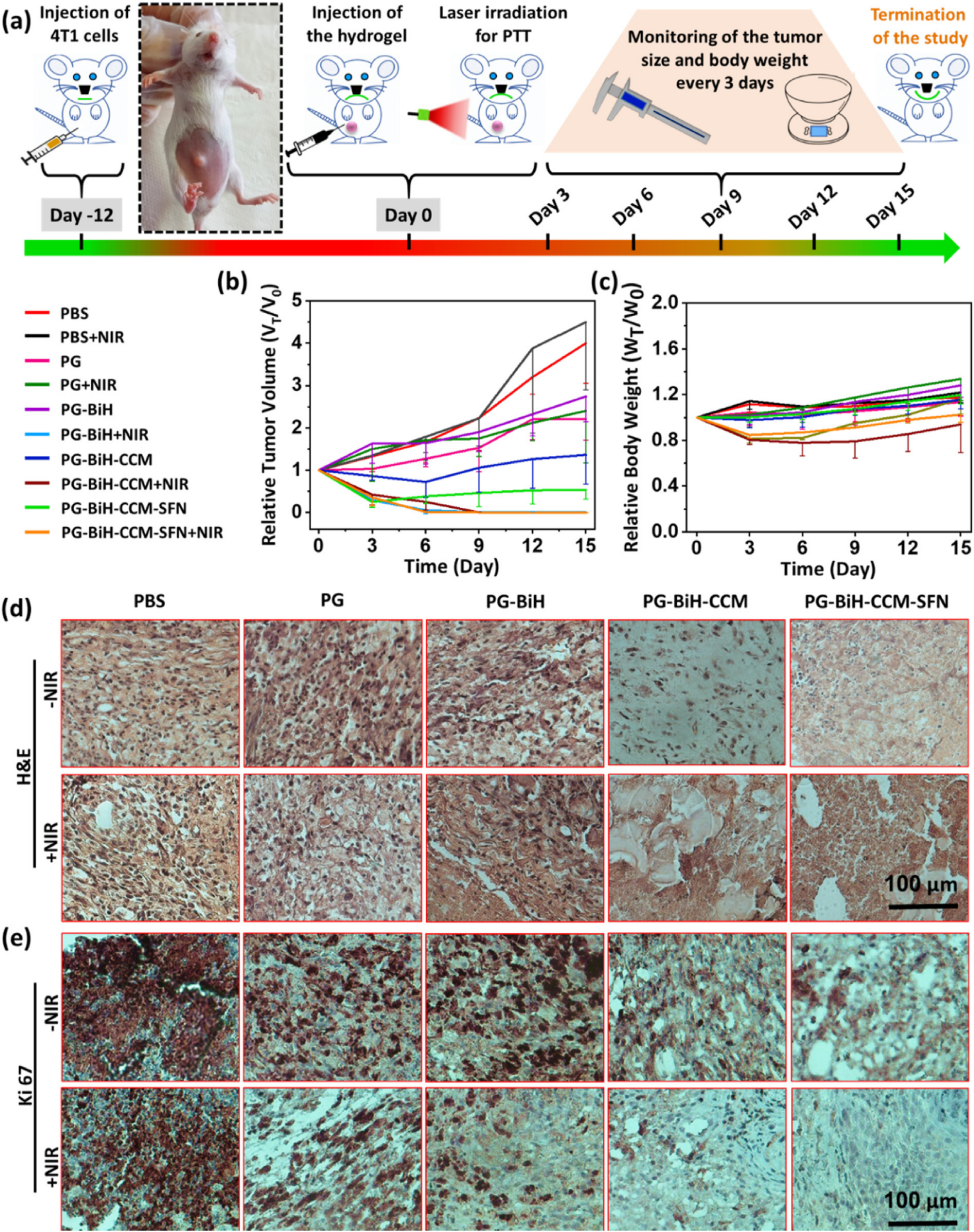
a) Schematic illustration of anti-tumor treatment method. b) Relative tumor volumes in the mice after different treatments with and without NIR irradiation. c) Relative body weight change of mice in different groups after treatment. d,e) Corresponding H&E and Ki67 staining of the tumor tissues after 15 days of treatment. Results are expressed as mean ​± ​SD (N ​= ​6).

The anti-tumor effect was evaluated on 4T1 bearing female BALB/c mice after intratumoral injection of different formulations, including PBS, PG, PG-BiH, PG-BiH-CCM, and PG-BiH-CCM-SFN with and without NIR irradiation. When the mice were subjected to laser irradiation (1.5 ​W ​cm^−2^) 2 ​h after injection, the temperature of the tumor site was rapidly increased to ≈52 ​°C within the 90 ​s in groups treated with BiH-loaded hydrogels and then remained constant for 3 ​min, which was high enough to kill the tumor cells. Relative tumor volume and animal body weight were recorded at 3-day intervals for 15 days (Fig. 8a). The relative tumor volume curves are shown in Fig. 8b. The tumor progression of mice treated with PG with or without laser irradiation and also PG-BiH in the absence of laser exposure was similar to the ones treated with PBS, indicating that PG ​± ​NIR or PG-BiH had no inhibitory effect on tumor progression. However, heat generation through irradiation of BiH nanorods presented in PG-BiH, PG-BiH-CCM, and PG-BiH-CCM-SFN hydrogels could effectively inhibit the growth of 4T1 tumors right after the treatment. The mice treated with PG-BiH-CCM and PG-BiH-CCM-SFN without laser irradiation showed remarkable tumor inhibition at the beginning of the treatment, but after 6 and 3 days, respectively, they showed tumor growth with a slight rate over 15 days. In fact, BiH-induced photothermal therapy provides a highly efficient strategy for tumor ablation, and coating of Bi_2_S_3_ nanorods with HA causes the longer remain of particles in the tumor region by binding to CD44 receptors, for multiple photothermal tumor heating in case of necessity. In fact, the higher interaction of the BiH with the cancer cells (Fig. S30) would promote the efficiency of PTT. However, despite its effectiveness in eradicating primary tumors, PTT alone still faces several challenges, including a lack of deep heating of cancer cells and the inability to remove all cancer cells in the edges of tumor tissues [66]. Therefore, PTT as monotherapy can lead to tumor metastasis and cancer recurrence [67]. The combined chemo-photo-immunotherapy showed better efficacy in controlling the tumor growth when compared to every single therapy, with no remarkable differences in the body weight changes in treated groups within 15 days (Fig. 8c). Moreover, the H&E staining results of tumor slices of different groups are shown in Fig. 8d, representing significant cellular necrosis in tumor section treated with PG-BiH-CCM-SFN hydrogel and NIR irradiation. This can be associated with the hyperthermia induced by BiH nanorods under laser irradiation, as well as SFN release (Fig. S31) and elicited immune responses from CCM. The results of antigen Ki-67 staining (Fig. 8e and S32) demonstrated that ≈70% of the tumor cells were Ki-67 positive in the PBS-treated group, showing high proliferation activity of primary cancer cells. The proliferation rate of 4T1 tumor cells was significantly suppressed in NIR-treated groups. However, in the PG-BiH-CCM-SFN ​+ ​NIR treated group, the number of Ki-67 positive proliferating tumor cells was less in comparison to PG-BiH ​+ ​NIR and PG-BiH-CCM ​+ ​NIR groups. Apart from the notable performance of PG-BiH and PG-BiH-CCM under laser irradiation in eliminating solid tumors, the PG-BiH-CCM-SFN hydrogel-mediated combined therapy can be effective in inhibiting tumor metastasis and cancer recurrence due to combination therapy [56,68]. In fact, SFN inhibits the action of tyrosine kinase Raf and other kinases involved in tumor cell growth and angiogenesis and additionally activates apoptosis to trigger cancer cell death. To support this claim, Yang et al. reported a combinatory chemo-photothermal system that completely eliminated the primary breast tumor and inhibited lung metastasis, while monotherapies were not sufficient for the complete eradication of the established tumor and led to cancer recurrence [69]. In fact, nanomedicine-based tumor PTT is a promising strategy for cancer ablation through the temperature-dependent killing of cancer cells, as well as induction of immunogenic cell death of tumor cells. When used in combination with immunotherapy, the efficacy of PTT can be improved. Immunotherapy is also able to eradicate both primary and distant tumors and potentially prevent tumor recurrence. Based on the study of Zhao et al., the combined photothermal immunotherapy strategy displays a stronger antitumor activity through the elimination of primary tumor and effectively inhibiting the growth of distant tumor while inducing an immune memory effect against tumor rechallenge [70]. In our study, to achieve immunotherapeutic effect, CCM was obtained from 4T1 cancer cells to alter the immunogenic physiology of the tumor microenvironment by local activation of antigen-presenting cells (APCs) that can later initiate tumor-specific immunity cascade by presenting antigens to T cells that are responsible for anti-tumor immune response [56,58,60]. This is the reason why the control group without CCM does not show an anticancer effect since T cells could not be activated. In contrast, the hydrogel with the CCM, even without PTT can reduce the growth of the tumor. This observation supports the fact that an injectable hydrogel with incorporated CCM can effectively show an anticancer response that could even be boosted in combination with the photothermal effect.

Overall, the combined chemo-photo-immunotherapy is an effective approach for the management of breast cancer [[22], [23], [24]]. In this context, further complementary studies should be performed in the future on metastatic models and evaluating the abscopal effect of the developed multitherapeutic hydrogel. Such studies will better demonstrate the tumor shrinking effect of the hydrogel and leads to our better understanding on its suppression efficacy on both primary and distant tumors. Moreover, since the hydrogel could be facilely injected into the solid tumor, it can minimize the unpleasant destruction of the surrounding normal cells. These data support our idea that the developed multifunctional and biocompatible hydrogel can be used as a promising combination therapy system for breast cancer.

## 3 Conclusions

In summary, we developed a multifunctional therapeutic hydrogel for the efficient synergistic treatment of breast cancer by loading HA-coated Bi_2_S_3_ nanorods as a photothermal agent, CCM as an immuno-adjuvant, and SFN as a chemotherapeutic agent. The simple and rapid fabrication of the developed hydrogels make them good candidates for clinical translation. Moreover, enhanced retention time and therapeutic effects after intratumoral injection of chemically cross-linked hydrogels through time-dependent increment in the viscosity was able to result in efficient anticancer effect. The NIR laser-based photothermal effect of this novel BiH-loaded hydrogel led to cancer ablation by localized heat generation in the tumor tissue. Furthermore, the synthesized Bi_2_S_3_ nanorods presented intrinsic antibacterial effects against both gram-positive and gram-negative bacteria as well as MRSA infection, which was synergized by PTT-assisted heat generation to prevent microbial infections during cancer therapy. Moreover, PTT induces tumor-related antigen release from destroyed cancer cells, leading to synergized immunostimulatory effect in combination with CCM in PG-BiH-CCM ​+ ​NIR treated group, by triggering antitumor immune response and cytokine release. Additionally, SFN incorporation in the hydrogel reduced the viability of 4T1 cells providing an opportunity for efficient eradication of aggressive tumors. In this study, a single round PTT with a single intratumoral injection of PG-BiH-CCM-SFN hydrogel revealed enhanced cancer therapeutic efficiency due to localized thermal cancer ablation, and drug retention at the tumor site. Moreover, this strategy showed strong tumor-specific immune responses, which can inhibit possible metastasis and tumor recurrence.

Overall, the combination of these approaches improve the outcome of cancer therapy and present a strategy to overcome current concerns on cancer resistance to chemotherapeutics, which can be expanded and used in the near future for a broad range of malignancies.

## 4 Material and methods

The experimental details are explained in the Supporting Information.

## Credit authors statement

**Samin Abbaszadeh**: Conceptualization, Investigation, Methodology, Writing – original draft. **Mohammad Reza Eskandari**: Resources, Conceptualization, Supervision, Methodology. **Vahideh Nosrati-Siahmazgi**: Conceptualization, Investigation, Methodology. **Kiyan Musaie**: Methodology, Investigation. **Soraya Mehrabi**: Methodology, Investigation. **Ruikang Tang**: Investigation, Data curation. **Mohammad Reza Jafari**: Conceptualization, Formal analysis, Validation. **Bo Xiao**: Formal analysis, Validation. **Vahid Hosseinpour Sarmadi**: Methodology. **Fakhri Haghi**: Methodology. **Bo Zhi Chen**: Investigation, Methodology. **Xin Dong Guo**: Supervision, Validation, Writing – review & editing. **Hélder A. Santosj**: Supervision, Validation, Writing – review & editing. **Mohammad-Ali Shahbazi**: Conceptualization, Supervision, Validation, Funding acquisition, Resources, Methodology, Formal analysis, Writing – review & editing.

## Declaration of competing interest

The authors declare that they have no known competing financial interests or personal relationships that could have appeared to influence the work reported in this paper.

## Data Availability

Data will be made available on request.
