# Supplementary material for: A photoactive injectable antibacterial hydrogel to support chemo-immunotherapeutic effect of antigenic cell membrane and sorafenib by near-infrared light mediated tumor ablation
Source: Mater Today Bio. 2023 Mar 14;19:100609. doi: 10.1016/j.mtbio.2023.100609 (PMC10034508; doi:10.1016/j.mtbio.2023.100609)
Supplement: Multimedia component 1 [file mmc1.docx]

**SUPPORTING INFORMATION**

**A Photoactive Injectable Antibacterial Hydrogel to Support**

**Chemo-Immunotherapeutic Effect of Antigenic Cell Membrane and Sorafenib by**

**Near-Infrared Light Mediated Tumor Ablation**

Samin Abbaszadeh^a^, Mohammad Reza Eskandari^b^, Vahideh Nosrati-Siahmazgi^c^, Kiyan Musaie^c^, Soraya Mehrabi^d^, Ruikang Tang^e^, Mohammad Reza Jafari^a^, Bo Xiao^f^, Vahid Hosseinpour Sarmadi^g^, Fakhri Haghi^h^, Bo Zhi Chen^i^, Xin Dong Guo^i^*, Hélder A. Santos^j,k,l^*, and Mohammad-Ali Shahbazi^j,k^*

^a^ Department of Pharmacology, School of Medicine, Zanjan University of Medical Sciences, 45139-56111 Zanjan, Iran

^b^ Department of Pharmacology and Toxicology, School of Pharmacy, Zanjan University of Medical Science, 45139-56184 Zanjan, Iran

^c^ Department of Pharmaceutical Biomaterials, School of Pharmacy, Zanjan University of Medical Science, 45139-56184 Zanjan, Iran

^d^ Department of Neuroscience, Faculty of Advanced Technologies in Medicine, Iran University of Medical Sciences, 14496-14535 Tehran, Iran

^e^ Center for Biomaterials and Biopathways, Department of Chemistry, Zhejiang University, Hangzhou, Zhejiang 310027, China

^f^ State Key Laboratory of Silkworm Genome Biology, College of Sericulture, Textile and Biomass Sciences, Southwest University, Chongqing, 400715 China

^g^ Cellular and Molecular Research Center, Iran University of Medical Sciences, 14496-14535 Tehran, Iran

^h^ Department of Microbiology and Immunology, School of Medicine, Zanjan University of Medical Sciences, 45139-56111 Zanjan, Iran

^i^ Beijing Laboratory of Biomedical Materials, College of Materials Science and Engineering, Beijing University of Chemical Technology, Beijing, China

^j^ Department of Biomedical Engineering, University Medical Center Groningen, University of Groningen, Antonius Deusinglaan 1, 9713 AV Groningen, Netherlands

^k^ W.J. Kolff Institute for Biomedical Engineering and Materials Science, University of Groningen, Antonius Deusinglaan 1, 9713 AV Groningen, The Netherlands

^l^ Drug Research Program, Division of Pharmaceutical Chemistry and Technology, Faculty of Pharmacy, University of Helsinki, FI-00014 Helsinki, Finland

*Correspondence to:

Prof. Xin Dong Guo; Email [xdguo@buct.edu.cn](mailto:xdguo@buct.edu.cn)

Prof. Hélder A. Santos; Email [h.a.santos@umcg.nl](mailto:h.a.santos@umcg.nl)

Prof. Mohammad-Ali Shahbazi; Email [m.a.shahbazi@umcg.nl](mailto:m.a.shahbazi@umcg.nl)

**1. Material and methods**

1.1. Synthesis of Nanorods: Bi_2_S_3_ nanorods were prepared using a simple chemical reaction [1]. Briefly, 121.25 mg of bismuth (III) nitrate pentahydrate (Bi(NO_3_)_3_.5H_2_O; 98%, SAMCHUN, South Korea) was dissolved in 3 ml of deionized water (DW) at room temperature under vigorous stirring. Next, 260 mg of thioacetamide (C_2_H_5_NS; >98%, Merck, Germany) was dissolved in 2 ml of DW before adding it to the Bi(NO_3_)_3_^.^5H_2_O solution under stirring to obtain a homogenous solution. The pH of the mixture was adjusted to 0.5 using HCl solution and the temperature was raised and maintained at 50 °C under stirring at 500 rpm for 4 h till the mixture solution changed its color from yellow to black as an indication of the Bi_2_S_3_ formation. In the next step, to produce BiH nanorods, 375 mg of hyaluronic acid (HA; High molecular weight, Bloomage Biotechnology Corp., Ltd., China) was dissolved in 10 ml of NaOH solution (2 M), and it was added slowly to the above mixture within 10 min while the nanorods were under probe sonication. The sample was then kept under constant stirring (500 rpm) at room temperature for 24 h. Finally, the black pellet of the BiH nanorods was collected through centrifugation at 14550 g for 10 min, washed 3 times with DW, and kept at 4 °C for further usage.

1.2. Isolation of CCM from 4T1 Cells: 4T1 cells were maintained in Roswell Park Memorial Institute (RPMI) 1640 medium supplemented with 10% (v/v) fetal bovine serum (FBS; Gibco, USA), 1% (v/v) L-glutamine, 1% (v/v) penicillin-streptomycin, and 1% (v/v) non-essential amino acid (KeyGEN, China). After washing with the 1×PBS (pH 7.4), the 4T1 cells were detached using PBS- ethylenediaminetetraacetic acid (EDTA, Gibco, USA; pH 7.4), and afterward, all the collected cells were centrifuged at 86 g for 5 min and washed three times with PBS (pH 7.4). Then, 8×10^7^ cells were suspended in the lysing buffer containing 20 mM Tris-HCl (Sigma-Aldrich, USA), 10 mM KCl (Sigma-Aldrich, USA), 2 mM MgCl_2_ (Sigma-Aldrich, USA), and one EDTA-free protease inhibitor mini-tablet (ThermoFisher, USA). Next, the cells were centrifuged at 3100 g for 5 min to collect the supernatant. Next, the lysing buffer was added again to the re-suspended precipitation, followed by centrifuging at 3100 g for 5 min to collect more of the supernatant. The separated supernatant was centrifuged in the next step at 22×10^3^ g for 20 min using an ultracentrifuge (Optima Max, Beckman Coulter, USA). The obtained pellet was thrown away and the supernatant was centrifuged again at 100×10^3^ g for 12 min to collect the CCM. Next, the obtained CCMs were dispersed in 1 ml of the PBS buffer (pH 7.4) to prepare aliquots of 100 µl and store them at -80 °C for further usage in each batch of hydrogel formation [2].

1.3. Preparation of the Hydrogels: Initially, 10% w/v of poly (methyl vinyl ether-alt-maleic anhydride) (PMVE-MA; Sigma-Aldrich, St. Lo., USA; average molecular weight 1,080,000 Da) solution was prepared in DW under vigorous agitation for 10 h at 75 °C until getting a clear solution. To prepare the PG hydrogel, 300 μl of poly (ethylene glycol) diglycidyl ether (PEGDGE, Sigma-Aldrich, Japan), as the cross-linker, was added to 1.5 ml of PMVE-MA solution. Then, 500 μl of gelatin solution (5% w/v, Sigma-Aldrich, USA) was prepared under stirring at 500 rpm at 50°C and added to the above mixture to obtain the injectable hydrogel after 3 h of incubation at room temperature.

To prepare PG-BiH hydrogel, BiH nanorods were added to 1.5 ml of PMVE-MA solution to obtain the final concentration of 200 μg ml^-1^ of the nanorods in the final hydrogel after following the procedure explained above. PG-BiH-CCM and PG-BiH-CCM-SFN hydrogels were also prepared by adding the CCM (100 µl) alone or CCM and SFN (19.2 μg of the drug in 48 μl of acetone) to the PMVE-MA solution containing BiH nanorods before following the above-explained gel formation protocol. All drug molecules and CCM were incorporated within the hydrogel with 100% efficiency since they were mixed with the polymer solution before adding the crosslinker for hydrogel formation.

1.4. Characterization of Bi_2_S_3_ and BiH Nanorods: The morphologies of the nanorods were investigated using a field-emission scanning electron microscope (FE-SEM, TESCAN MIRA3, Czech Republic) and transmission electron microscope (TEM, Phillips EM 2085, USA) and the elemental compositions were determined by energy-dispersive X-ray spectrometry (EDAX) for Bi_2_S_3_, BiH, and different hydrogels. The particle size distribution of the nanorods was quantified based on the obtained TEM and FE-SEM images.

The zeta- potential of Bi_2_S_3_ and BiH nanorods was detected using a zeta sizer (SZ-100z, Horiba Jobin Jyovin, Japan) at room temperature. The suspension stability of Bi_2_S_3_ and BiH nanorods dispersed in DW was also monitored over 24 h. In addition, the absorption spectra of Bi_2_S_3_ and BiH nanorods were recorded in the range of 450 to 850 nm using UV-vis spectroscopy (Genesys 10-S, USA) to observe their ability of light absorption, particularly in the NIR area. To determine the specific surface area of the synthesized nanorods, Brunauer-Emmett-Teller (BET) analysis was conducted using the Belsorp mini II instrument (Microtrac Bel Corp., Japan).

1.5. Characterization of Hydrogels

*1.5.1. Gelation Time and Elemental Analysis:* To evaluate the gelation time, the inverted tube test was used [3]. For this aim, 1 ml of prepared PG and PG-BiH hydrogels with and without PEGDGE as a cross-linker, were periodically maintained at 55 °C and inverted to check the sample flow behavior under the influence of gravity. The time at which the gel did not flow was recorded as the gelation time. Moreover, to evaluate the effect of PMVE-MA and gelatin in gelation process, 1 ml of PMVE-MA or gelatin with crosslinker were prepared and the inverted tube test was used to check the gelation at 55 °C. In addition 1 ml of PG-BiH hydrogel was fabricated and monitored 8 h, 24 h, and 48 h after preparation to evaluate the colloidal stability of nanorods in the hydrogel matrix. The EDAX analysis and elemental mapping of PG and PG-BiH (in the dried form) were also conducted by FE-SEM, TESCAN MIRA3, Czech Republic.

*1.5.2. Swelling and Degradation:* To determine the swelling characteristics of the hydrogels, at first, the PG and PG-BiH hydrogels were prepared and weighted when dried completely (W_d_). Next, the samples were immersed in 50 ml of phosphate buffer saline (PBS; pH 5.8 and pH 7.4) at 37 °C. The swelled hydrogels were weighted (W_s_) at different time points (1, 3, 6, 9, 24, 48, and 72 h) after removing the excess water on the surface of the hydrogel by mildly blotting onto a filter paper. The swelling ratio was calculated using the Equation (1):

$Swelling\%=\frac{(W_{s}-W_{d})}{(W_{d})}\times100$ (1)

To evaluate the percentage of degradation, the completely dried PG and PG-BiH hydrogels were weighted (W_initial_) and immersed in 50 ml of PBS solution (pH 5.8 and pH 7.4) at 37 °C. After 24 and 96 h, the hydrogels were taken out from the PBS solution and dried in the oven at 70 ºC for 48 h (W_d_). The percentage of degradation was calculated using the Equation (2):

$Degradation\%=\frac{(W_{\mathrm{Initial}}-W_{d})}{W_{\mathrm{Initial}}} \times100$ (2)

*1.5.3. Initial Water Content and Yield:* To calculate the initial water content (IWC), the PG and PG-BiH hydrogels were prepared and their initial wet weight was measured. Then, the hydrogels were dried completely at 70 °C in the oven for 72 h (W_d_) and their weights were measured again and the IWC (%) was calculated using Equation (3):

$$\mathrm{IWC}\left( \% \right)=\frac{(Initial wet weight of the hydrogel-W_{d})}{Initial wet weight of the hydrogel}\times100 (3)$$

In order to study the yield, PG and PG-BiH hydrogels were dried at 70 °C in the oven for 72 h and after that, the weight of dried hydrogels was measured and the yields for both types of hydrogels were calculated using Equation (4) and (5):

$$Yield\% (PG)=\frac{Weight of dried PG hydrogel}{The initial weight of ((PMVE-MA)+ PEGDGE+ Gelatin)}\times100 (4)$$

$$Yield\% \left( PG-BiH \right)=\frac{Weight of dried PG-BiH hydrogel}{Initial weight of ((PMVE-MA)+ BiH+PEGDGE+Gelatin)}\times100 (5)$$

*1.5.4. FTIR, XRD, TGA, and DTG Analysis:* To validate the successful synthesis of hydrogels, an attenuated total reflectance-Fourier transform infrared spectroscopy (ATR-FTIR; Thermo Nicolet Avatar, USA) test was conducted in the wavelength range of 4000-600 cm^-1^. The crystal structures of the samples were also analyzed by X-ray diffraction (XRD) analysis with 2θ range of 10-80° θ (XRD Philips PW1730, Netherlands). Thermogravimetric analysis (TGA; SDT-Q600, USA) and derivative thermogravimetry (DTG) analysis were used to study the thermal stability and phase transitions within the heating temperature range of 30 to 800 °C and with a heating rate increment of 10 °C min^-1^ under argon atmosphere. All above studies were also performed for the as-prepared nanorods before incorporating them into the final hydrogel.

*1.5.5. Measurement of Injection Force for the Hydrogels:* Mechanical testing machine (SANTAM, STM 5, Iran) was used for the assessment of injectability of various hydrogels 3 h, 12 h, and 24 h after preparation. The needed force was assessed for hydrogels incubated at 25 ˚C or 37 ˚C using a 500 N load cell with a speed rate of 1 mm s^-1^ [4]. PG and PG-BiH hydrogels were loaded into 10-ml syringes and then needles with 21 gauge (G) and 22 G but the same length were attached to different syringes. Displacement and force were recorded until the hydrogels in the syringes were discharged. Then all data were extracted and the force was plotted against displacement.

*1.5.6. Rheological Studies*: The viscosity of PG and PG-BiH hydrogels were assessed over shear rates ranging from 0 to 200 (s^−1^), at 3 h, 24 h, and 10 days after preparation of the hydrogels using a rheometer (R/S plus, Brookfield, Canada) with parallel plate geometry (25 mm diameter). Moreover, the PG and PG-BiH hydrogels were subjected to rheological measurement using a rotary rheometer (Anton Paar, MCR302, Austria). The test was performed 24 h and 10 days after the preparation of hydrogels. The storage modulus (G') and loss modulus (G'') of the hydrogels were evaluated using frequency sweep measurements (x=0.1–100 rad s^-1^ at 0.1% strain) and dynamic oscillatory stress sweep (c=0.01–100% at 10 rad s^-1^) to ensure the linear viscoelastic properties. The self-healing ability of the hydrogel was studied by alternating strain of 0.1% and 200% at a time interval of 100 s at a constant angular frequency of 10 rad s^-1^.

Additionally, the bio-adhesivity, stretchability, and self-healing behavior of hydrogels were characterized by macroscopic photographs. The adhesion performance of the hydrogels to the surface of different tissues, including skin, heart, kidney, and spleen were assessed. Moreover, the stretchability of the prepared hydrogels was evaluated by placing them between two fingers and stretching them. For self-healing test, PG and PG-BiH hydrogels were prepared with a diameter of 17 mm and a thickness of 8 mm. Two separate disc-shaped hydrogels were closely attached and left at room temperature for 3 min to evaluate their self-healing property by visualizing their integration to form a single disc-shaped hydrogel. All the above studies were conducted using the gels 10 days after preparation.

1.6. Photothermal Behavior of Bi_2_S_3_, BiH Nanorods, and Hydrogels: Under NIR laser irradiation (808 nm) with power densities of 1 and 1.5 W cm^-2^ over a period of 10 min, the temperature elevation of the Bi_2_S_3_ and BiH nanorods in DW (100, 200, and 400 µg ml^-1^) as well as the hydrogels were evaluated. A digital NIR camera was used to record temperature changes.

To characterize the photothermal stability of BiH nanorods, the PG-BiH-CCM-SFN hydrogel with 200 μg ml^-1^ of BiH was exposed to NIR irradiation for 10 min at 1.5 W cm^-2^ and then the sample was cooled down naturally to room temperature, and this process was continued for five cycles. The temperatures of the sample were recorded in all cycles.

*1.6.1 Photothermal Conversion Efficiency:* To investigate the photothermal conversion efficiency (η), PG-BiH-CCM-SFN hydrogel was irradiated with a NIR laser for 10 min at 1.5 W cm^-2^. Then, the laser was turned OFF and the sample was cooled naturally until reaching the ambient temperature and the temperature was recorded every 30 s in the cooling phase. The ƞ value of the BiH nanorods loaded in PG-BiH-CCM-SFN hydrogel was calculated according to Equation (6):[5]

$ƞ=\frac{hA(T_{\mathrm{Max}}-T_{\mathrm{Surr}})-Q_{\mathrm{Dis}}}{I (1-{10}^{-A808})}$ (6)

Where h exhibited the heat transfer coefficient. A was the surface area of the container. T_Max_ represents the maximum steady-state temperature (60.3 °C) and T_surr_ corresponds to the environmental temperature (28 °C), respectively. Therefore, the temperature change (T_Max_-T_Surr_) of the hydrogel was 32.3 °C. Q_Dis_ is heat dissipated from the light absorbed by the solvent and container. I represents the laser power (1500 mW cm^-2^), and A_808_ was the absorbance intensity of the BiH nanorods at 808 nm (0.67). The value of hA was calculated from Equation (7):

$\tau_{s}=\frac{m_{D}\times C_{D}}{\mathrm{hA}}$ (7)

The time constant (τ_s_) of the sample can be calculated using Equation (8):

$t=- \tau$_s_ ln(θ) (8)

and θ was calculated as follows Equation (9):

$\theta=\frac{T-T_{\mathrm{Surr}}}{T_{\mathrm{Max}}- T_{\mathrm{Surr}}}$ (9)

The τ_s_ for heat transfer from the sample is assessed to be τ_s_=250.3 (Figure 4j) by using the linear time data from the cooling period versus the negative natural logarithm of driving force temperature, which is taken from the cooling section of the Figure 4i.

Therefore, the hA value can be calculated from Equation (7). m_D_ and c_D_ were the mass (0.8 g) and heat capacity (4.2 J g^-1^ **°**C^-1^) of the DW present in the PG-BiH-CCM-SFN hydrogel, respectively. So the hA was calculated to be 13 mW °C^-1^. Q_Dis_ exhibited the heat dissipation from the light absorbed by the DW and the container, and was calculated according to Equation (10):

$Q_{\mathrm{Dis}}=\frac{m_{D}\times c_{D}(T_{\mathrm{Max}}-T_{\mathrm{Surr}})}{\tau_{s}}$ (10)

Where the m_D_ (the mass of DW irradiated upon 808 nm laser (1.5 W cm^-2^) for 10 min) was 1 g, T_max_ (the maximum temperature of DW after 10 min NIR irradiation) was 29.9 °C, T_Surr_ of the DW was 23 °C, and its τ_s_ was 519.5. According to these values, the final calculated Q_Dis_ was 55 mW. In addition, the η value of the BiH nanorods loaded in PG-BiH-CCM-SFN hydrogel was calculated to be 30.9%, according to the Equation (6).

*1.6.2. In Vivo Photothermal Behavior of BiH Nanorods and Hydrogels:* To assess the in vivo photothermal potential of PBS, PG, BiH, and PG-BiH, they were injected subcutaneously (*s.c.*) into the mice (100 μl) and irradiated by NIR laser for 5 min (808 nm, 1.5 W cm^-2^). In addition, the mice received the *s.c.* injection of PG-BiH hydrogel (100 μl) and 2 h later, the injected area was exposed to an 808 nm laser with different powers of 0.65, 1, and 1.5 W cm^-2^ for 8 min while the temperature was monitored and imaging was conducted by a thermal camera.

1.7. Cell Viability Studies: To evaluate the cell viability of the Bi_2_S_3_ and BiH nanorods, approximately 1.5×10^4^ 4T1 cancer cells were seeded in each well of the 96-wells plate overnight before the treatment with nanorods. The culture medium was then replaced with the fresh culture medium containing Bi_2_S_3_ and BiH with different concentrations (50, 100, 200, and 400 μg ml^-1^) and incubated for 24 and 48 h. Afterward, the samples were gently washed by the Hank’s balanced salt solution (HBSS)−(4-(2-hydroxyethyl)-1 piperazineethanesulfonic acid (HEPES) (pH 7.4) for one time before using CellTiter-Glo to test viability by a multimode microplate reader (Varioskan LUX, ThermoFisher, USA). A cell culture medium without any tested materials was used as control. All samples were tested in 4 replicates.

Moreover, 4T1 cancer cells were used for the cytotoxicity study of different hydrogels. The cells were seeded in a 24-well Transwell^®^ plate at 8×10^4^ cells per well. The cells were allowed to attach overnight. Then the freshly prepared PG, PG-BiH, PG-BiH-CCM, and PG-BiH-CCM-SFN hydrogels (350 µl final volume) were added to the upper chamber of the Transwell^®^. The samples were incubated at 37 ˚C for 24 h. A cell culture medium without any tested materials was used as the negative control. After treatment, cell viability was measured using a CellTiter-Glo^®^ luminescent assay by the transfer of the reagents and cells to a 96-well plate after treatment with CellTiter-Glo^®^ reagents. Varioskan™ LUX multimode microplate reader was used to read the luminescence of the samples. All samples were tested in 4 replicates.

Furthermore, the in vitro PTT treatment effects were assessed in 4T1 cells. 4T1 cells were seeded in a 96-well plate at a density of 1.5×10^4^ cells per well one day prior to the experiment. Then the cell culture medium in each well was replaced by 75 µl of HBSS-HEPES buffer, and 75 µl PG or PG-BiH hydrogels were injected into the wells. Cells with PBS injection were used as controls. The cells receiving in vitro PTT treatment were exposed to NIR laser (808 nm) at 1 W cm^-2^ for 10 min. After the laser exposure, the gel and HBSS-HEPES buffer were removed from the wells and the cell viability was measured using a CellTiter-Glo^®^ luminescent assay according to the manufacturer’s protocol. The luminescence intensity was obtained by a Varioskan™ LUX multimode microplate reader.

1.8. Interaction of the Nanoparticles with Cancer Cells: Flow cytometric analysis was conducted to evaluate the cellular interaction of Bi_2_S_3_ and BiH nanoparticles with the breast cancer cells. The cells were first seeded in 6-well plates at a density of 7×10^5^ cells per well and allowed to attach overnight at 37 °C. The attached cells were subsequently washed with HBSS–HEPES (pH 7.4) and exposed to the Alexa Fluor 488-labeled nanoparticles at the concentrations of 100 μg ml^−1^ for 8 h and 24 h at 37 °C. The cells were then washed gently with PBS (pH 7.4) for two times and harvested using 300 μl of 0.25% trypsin–PBS–EDTA solution. The cells were centrifuged at 22 g for 3 min and washed two times with PBS (pH 7.4) again to remove the non-associated nanoparticles. Next, the cells were fixed with 2.5% glutaraldehyde in PBS buffer (pH 7.4) for 30 min and re-suspended in 700 μl of PBS (pH 7.4). The measurements were performed at a laser excitation wavelength of 488 nm and a minimum of 10
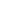
000 events per sample were collected. The data were analyzed using Flowjo 7.6 software (Tree Star, Ashland, USA).

1.9. Hemocompatibility of the Hydrogels: The hemolytic potential of the hydrogels was assessed by measuring the absorbance of released hemoglobin after the red blood cell (RBC) lysis. Blood was taken from a healthy human volunteer and it was drawn into K_2_-EDTA-coated tubes to prevent coagulation. 20 ml of PBS buffer (pH 7.4) was added to 10 ml of blood and the mixture was centrifuged at 1200 g for 6 min. This step was repeated 5 times to completely wash blood cells by aspirating the supernatant after each centrifugation step. Next, the final obtained RBCs were diluted with PBS buffer (pH 7.4) to obtain a concentration of 5% (v/v). Dried PG, PG-BiH, and PG-BiH-CCM hydrogels were dispersed in 800 μl of PBS buffer (pH 7.4) and then the suspension was mixed with the 200 μl of RBC suspension to reach the final concentration of 0.5, 1, 1.5 and 2 mg ml^-1^ in a 1.5 ml microtube. After incubating at room temperature for 2 h, 4 h, 8 h, and 24 h, the samples were centrifuged at 3000 g for 5 min. Then, 150 µl of the supernatants were pipetted to a 96-well plate and the absorbance of the samples was recorded at 540 nm using a plate reader (infinite M200, Austria). DW was used as a positive control and PBS buffer (pH 7.4) as a negative control. Each sample was studied in triplicate and the percentage of non-hemolyzed RBC was calculated for each sample using Equation (11):

$$Non-hemolyzed RBC \%=100-\left( \frac{Absorbance of (sample- negative control)}{Absorbance of (positive control-negative control)}\times100 \right) (11)$$

1.10. In Vitro Immunological Transwell^®^ Experiment: 4T1 cells (10^5^ cells per well) were seeded into the upper transwell^®^ chamber and cultured overnight. Then, the cells were administered with PBS, PG, PG-BiH, and PG-BiH-CCM hydrogels. After 2 h incubation, the 4T1 cells were irradiated by 808 nm laser (1.5 W cm^−2^, 5 min). Then bone marrow-derived dendritic cells (BMDCs) were cultured in the lower transwell^®^ compartment for 24 h at 5×10^5^ cells per well. The BMDCs were collected and rinsed by PBS. Finally, the BMDCs stained by anti-CD80 and anti-CD86 were detected by ﬂow cytometry. The supernatants of co-cultured medium were collected to quantify cytokines including tumor necrosis factor-alpha (TNF-α), interleukin 6 (IL-6), interferon-gamma (IFN-γ) using ELISA kits.

1.11. Antibacterial Studies

*1.11.1. In Vitro Antibacterial Activity of BiH Nanorods and PG-BiH Hydrogel:* To evaluate the antibacterial activity and photothermal-bactericidal activity of BiH nanorods and PG-BiH hydrogel, both Gram-positive Staphylococcus aureus (*S. aureus*) (ATCC 25923) and Gram-negative Escherichia coli (*E.* *coli*) (ATCC 25922) bacteria were tested in this study using colony counting assay.

Both types of bacteria were suspended in sterilized nutrient broth before incubating in a shaking incubator (150 rpm) at 37°C overnight. Then, the 0.5 McFarland turbidity standard bacteria solution was prepared and diluted to reach the final concentration of 0.5×10^8^ CFU ml^-1^ with normal saline (0.9% w/v). Next, 20 μl of the obtained bacteria suspension (10^6^ CFU ml^-1^ bacteria) were added into 1 ml of an aqueous dispersion of BiH nanorods (200 μg ml^-1^), or PG-BiH hydrogel, which were initially prepared under sterile conditions. Normal saline was used as the control. The resulting mixtures then received laser irradiation for 10 min (1.5 W cm^-2^) followed by incubating at 25 °C for 2 h. Next, the mixture was diluted in two steps, initial via adding 2 ml of normal saline followed by adding another 9 ml of normal saline to 1 cc of acquired solution. Afterward, 100 µl of all diluted samples were dispersed uniformly on an agar plate and incubated at 37 °C overnight to visualize the colony formation on the plates. All samples were studied in triplicate.

*1.11.2. In Vivo Antibacterial Activity of BiH Nanorods and PG-BiH Hydrogel:* The in vivo antibacterial properties of BiH nanorods and PG-BiH hydrogel were assessed using the animal model of a subcutaneous abscess [6]. Firstly, the dorsal hair of each mouse was carefully shaved and the injected site was disinfected. Afterward, 100 μl of the Methicillin-resistant Staphylococcus aureous (*MRSA*; 1×10^7^ CFU ml^-1^) was injected into the back of each mouse. After 24 h, a subcutaneous abscess was observed on each side of the dorsal surface of the mice. Then the mice were randomly divided into eight groups, including healthy skin (healthy mice without any treatment), infected (infected mice without any treatment), PBS (with and without NIR exposure), BiH (with and without NIR exposure), and PG-BiH (with and without NIR exposure). For the latter three groups (with and without NIR exposure), 100 µl of PBS (pH 7.4), BiH (200 μg ml^-1^), or PG-BiH were directly injected into the infected site of each group, respectively. At 2 h post-injection, the abscess of the mice in the NIR-treated groups was exposed to an 808 nm NIR laser (1.5 W cm^-2^) in a way to keep the temperature of the infected site constantly at 50 °C for 3 min. After 13 days, the abscess was photographed and the infected tissues were harvested and fixed in 10% formalin for hematoxylin and eosin (H&E) and immunohistochemical staining for TNF-α identification. Moreover, the harvested bacteria from the infected tissues were immersed in 8 ml of PBS (pH 7.4) and homogenized. Next, the obtained samples were diluted 50 times in PBS (pH 7.4) and were cultured on agar plates by taking 100 µl of the diluted suspension. After 20 h of incubation, the growth of colonies was visualized.

**1.12. In Vivo Toxicity Evaluation of Hydrogels:** Twenty adult Wistar rats (male; 250±20 g) were randomly divided into 5 groups (N=4 in each group). To evaluate the toxicity of hydrogels, the animals were first anesthetized by *intraperitoneal* (*i.p.*) injection of 0.25 ml of ketamine (50 mg ml^-1^)-xylazine (20 mg ml^-1^) cocktail (6:4 v/v). Then, the four groups of animals were *s.c.* injected in the dorsal region with 1 ml of PG, PG-BiH, PG-BiH-CCM, and PG-BiH-CCM-SFN hydrogels. The animals in the control group received normal saline *s.c.* instead of the hydrogel. After 14 days, 3 ml of blood was collected from the heart of each animal for the assessment of blood biochemistry and hematological factors. In addition, the animals were scarified and the main organs, including the liver, kidney, spleen, and the skin layer of the injected area were collected for histopathological evaluation of the tissues by H&E staining and visualization under optical microscopy (Olympus BX61, Japan).

**1.13. In Vivo Combined Photothermal-Chemo-Immunotherapy Studies:** To assess the in vivo anticancer effect of the hydrogel, 1.5×10^6^ 4T1 cells were suspended in 100 μl PBS, and were *s.c.* injected into the mammary fat pad of female BALB/c mice. The tumor volume was measured using the following formula: Tumor volume = [(Tumor length)×(Tumor width)^2^]/2, where length shows the largest tumor diameter and width shows the perpendicular tumor diameter. When the tumor volume reached approximately 80 mm^3^ (after 10 days), mice were randomly divided into ten groups (N=6) for intratumoral injection of 100 μl of PBS (pH 7.4), PG, PG-BiH, PG-BiH-CCM, or PG-BiH-CCM-SFN hydrogels, all with and without NIR irradiation. The hydrogel formulations were injected 3 h after their fabrication. At 2 h after the injection of hydrogels, tumors of the mice in the NIR-treating groups were exposed to NIR light (808 nm, 1.5 W cm^-2^) until the temperature reached 52 °C and then remained constant for 3 min while tumor temperature was monitored by an IR thermal camera. The body weight of all animals and tumor dimensions (by a digital caliper) were measured every 72 h. Relative tumor volumes (RTV) were calculated by the following Equation: RTV=(tumor volume on a measured day)/(tumor volume on day 0). The mice were sacrificed on day 15 and all tumors were collected and fixed in 10% formaldehyde for histological analysis.

For all animal studies, the animals were properly given food and water ad libitum and were housed in the animal room at a controlled ambient temperature of 22±2 °C with 50±10% relative humidity and a 12-h light/12-h dark cycle. The study was approved by the Ethics Committee of Zanjan University of Medical Sciences (IR.ZUMS.REC.1399.168).

2. **Results and Discussion**

**Table S1.** BET analysis of the Bi_2_S_3_ and BiH nanorods, representing the adsorbed volume of nitrogen, which corresponds to a monolayer coverage (V_m_), and surface area.

**
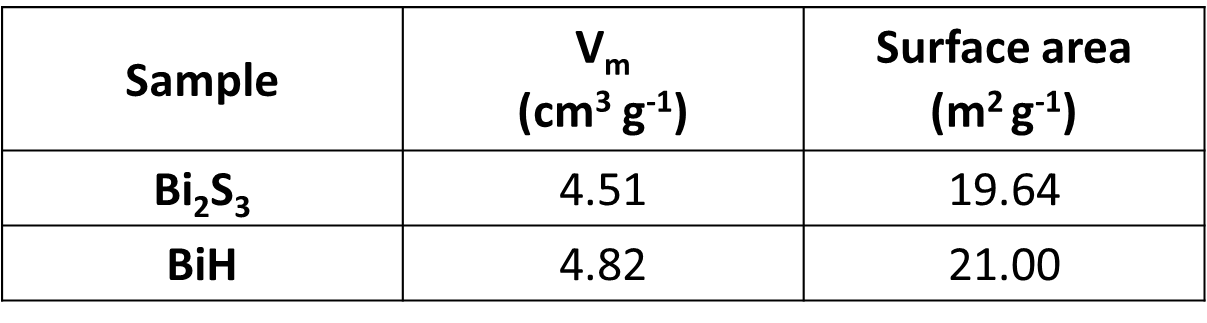
**


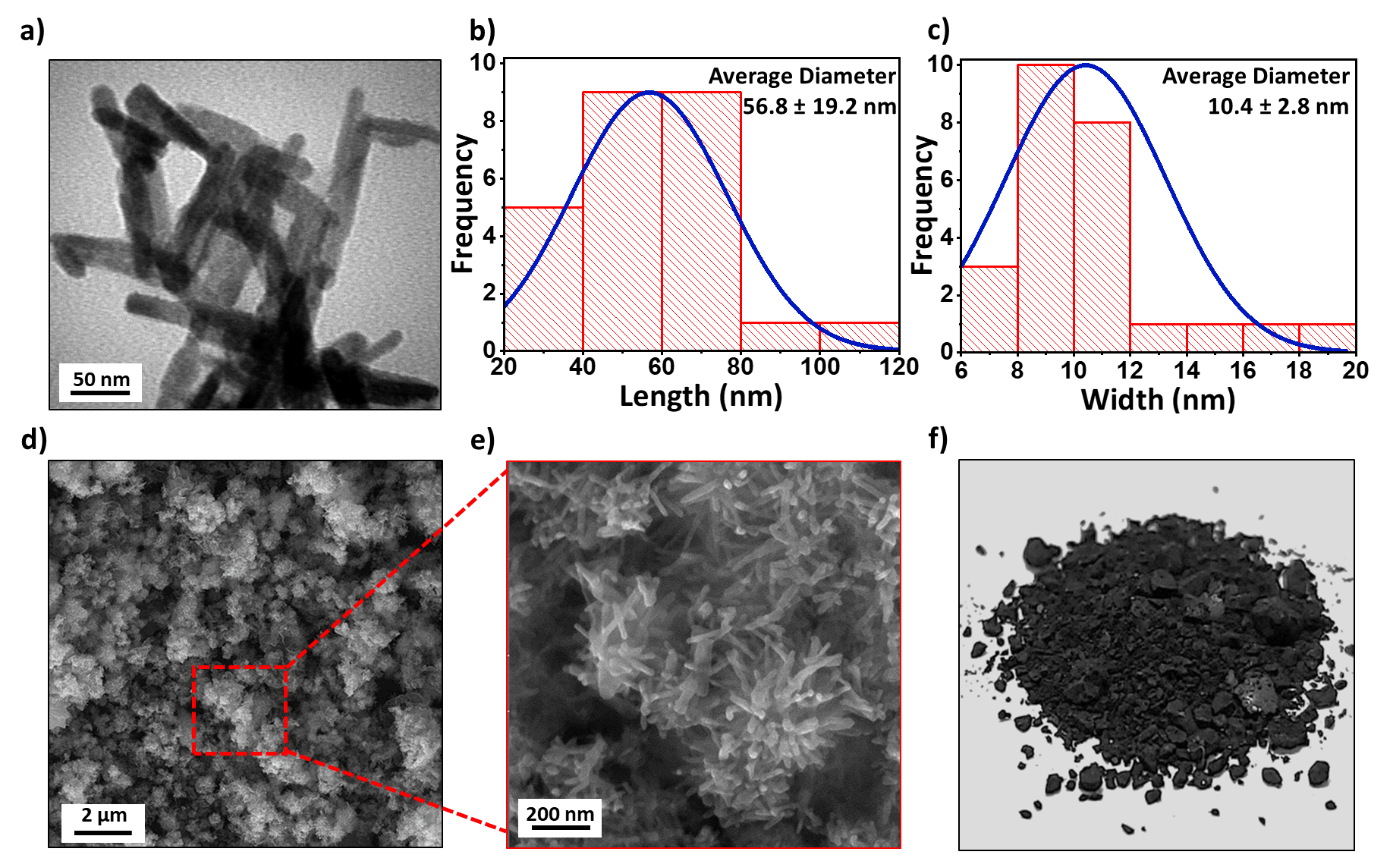


**Figure S1.** a) TEM image of the Bi_2_S_3_ nanorods. b,c) Length and width of the Bi_2_S_3_ nanorods obtained from TEM image. d,e) FE-SEM images of Bi_2_S_3_ nanorods under different magnifications. f) The photograph of the Bi_2_S_3_ nanorods.


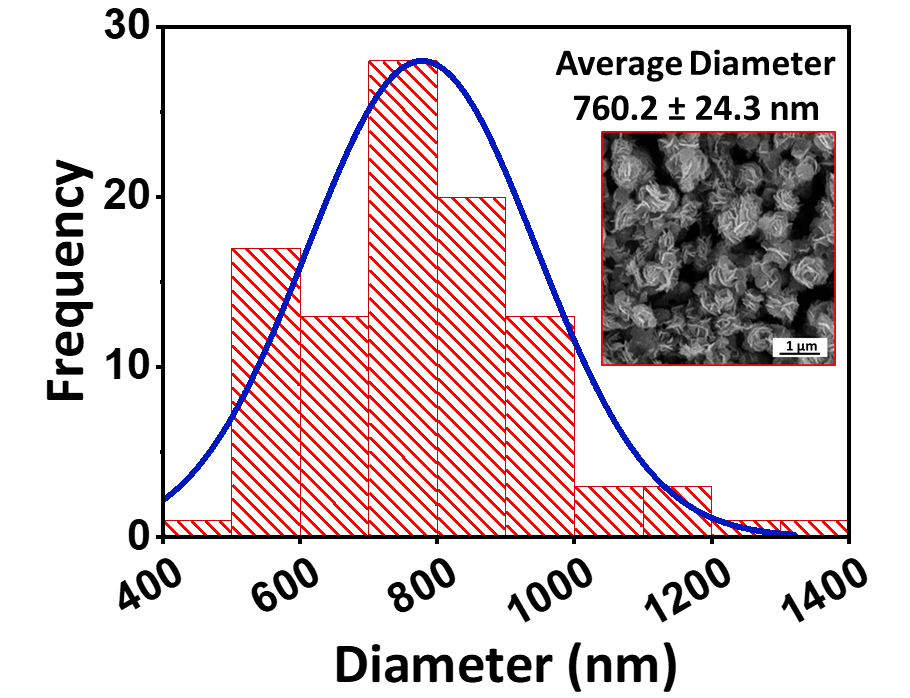


**Figure S2.** The size distribution of the microspheres formed by the self-assembly of the BiH nanorods. The quantitative measurement was conducted on FE-SEM image using ImageJ software.


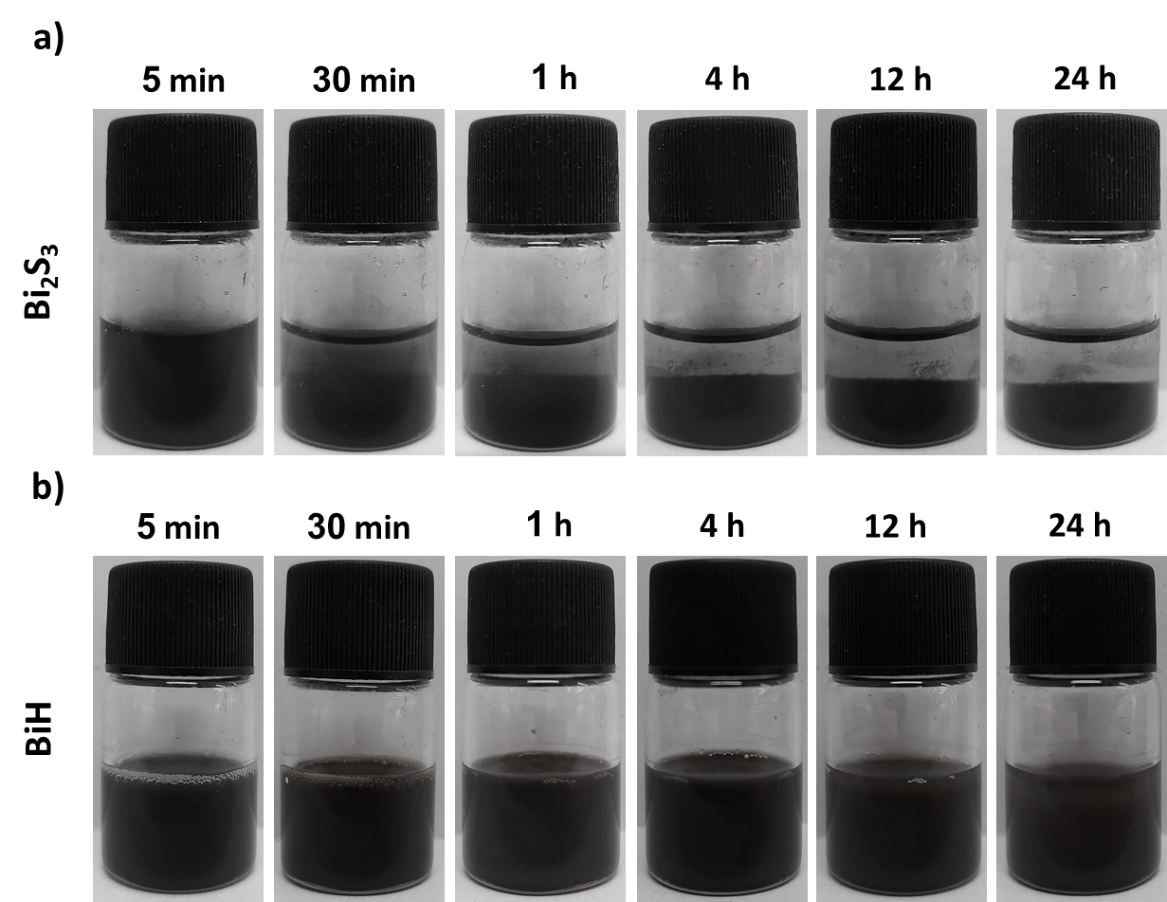


**Figure S3.** The effect of HA coating on the stability of nanorods dispersed in DW for 24 h. The precipitation rate of the a) Bi_2_S_3_ and b) BiH nanorods.


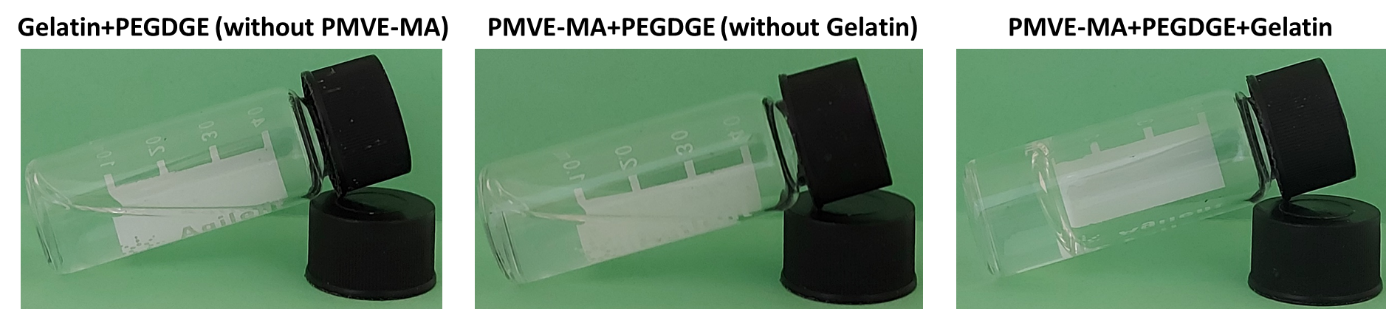


**Figure S4.** Digital images of various formulations 20 h after preparation at 55 °C. The images show both polymers are needed for the crosslinking and gel formation.


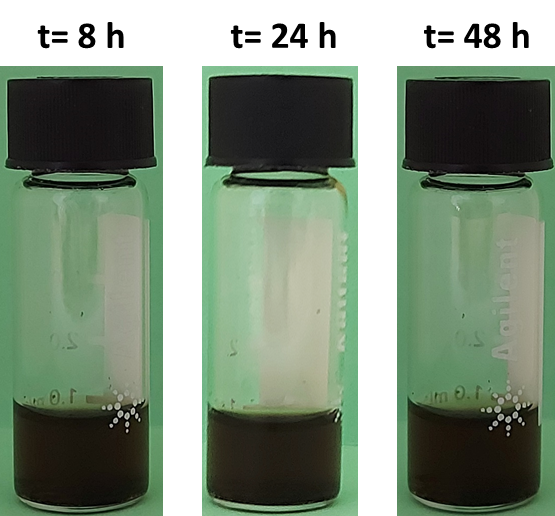


**Figure S5.** The stability of BiH nanorods dispersed in the hydrogel matrix 8 h, 24 h, and 48 h after preparation.

**
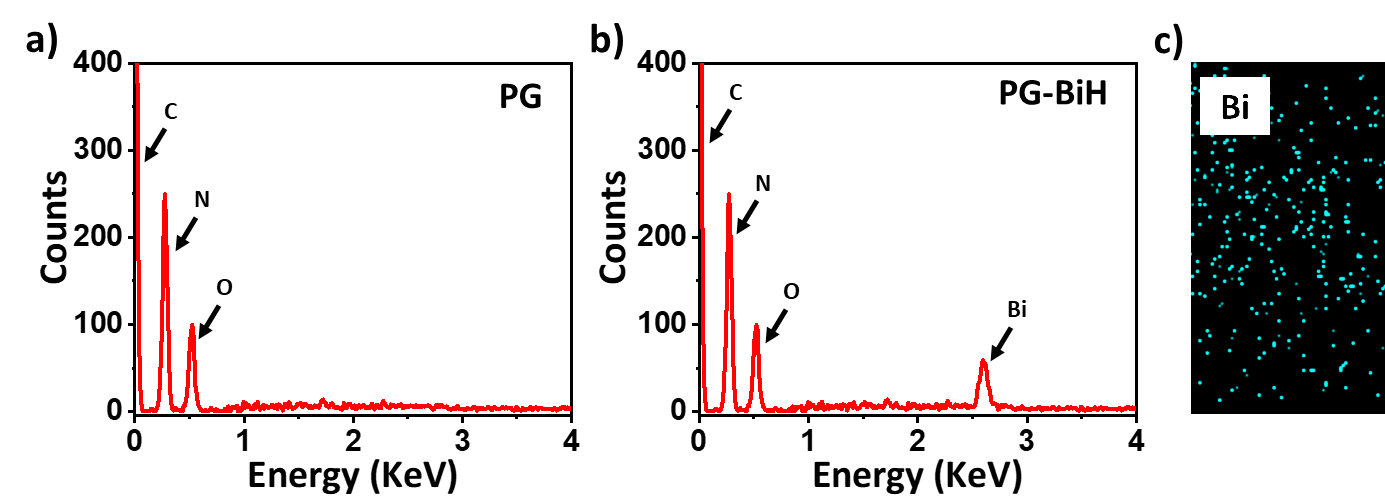
**

**Figure S6.** EDAX analysis of a) PG and b) PG-BiH hydrogels. EDAX was used to determine the elemental content of the hydrogels and showed the presence of C, O, and N in the PG hydrogel and C, O, N, and Bi in the PG-BiH hydrogel, which confirms the loading of BiH nanorods in the hydrogel. c) Elemental mapping of Bi in PG-BiH hydrogel, showing the uniform dispersity of the particles in the matrix of the hydrogel.

**The Swelling and Degradation Rate of the PG and PG-BiH Hydrogels**

Swelling is an important factor that can define the biological property and cargo release rate of a hydrogel [7]. Therefore, the swelling behavior of the PG and PG-BiH hydrogels were evaluated at different time points (1, 3, 6, 9, 24, 48, and 72 h) at two different pH values of 5.8 to mimic the microenvironment of the tumor site, and pH 7.4 to simulate the physiological environment [8]. Figure S7a shows the swelling ratio of both hydrogels was increased for 9 h and after that reached a steady state. The reason for this constant swelling ratio over time is that when the volume of hydrogels increased, polymer chains were stretched by the penetration of water molecules across the cross-linked network, and after that, the polymer chains were not expandable by crosslinking. The ratio of swelling was lower at pH 5.8 as compared to pH 7.4. The pH-responsive swelling behavior of hydrogels is due to the ionization of the functional groups in the gels, which be dependent on the pH of the surroundings. PMVE-MA has maleic anhydride that can be hydrolyzed into carboxyl groups when in contact with water. This observation can be explained by the fact that the PMVE-MA as a polyacid could deprotonate at neutral pH and therefore electrostatic repulsion forces induce the swelling of the polymer. However, at the pH of 5.8, the hydrolyzed polymer groups are protonated and the polymer chains shrink, so reduced the transfer of water into the hydrogels. In addition, an increase in the swelling ratio of PG-BiH hydrogel was observed compared to the PG hydrogel in neutral pH, which is attributed to the interference of BiH nanorods with the crosslinker and increased penetration of water molecules across the cross-linked network. However, it is the same in pH 5.8 due to the shrinkage of the polymer chains.

The degradation of the PG and PG-BiH hydrogels was studied in PBS with different pH values (pH 7.4 and 5.8) at 37 ˚C. The results of degradation are in line with swelling. As shown in Figure S7b, the rate of degradation was a little higher in acidic pH. This result demonstrated that the acidic environment could increase the degradation rate of the hydrogel, which would accelerate the release of incorporated drugs after intratumoral injection. In addition, the PG-BiH hydrogel showed a higher rate of degradation, which might be due to the interference of BiH nanorods with the crosslinker during the hydrogel formation through the chemical reaction of the functional groups in polymers and the epoxides of the crosslinker.

**
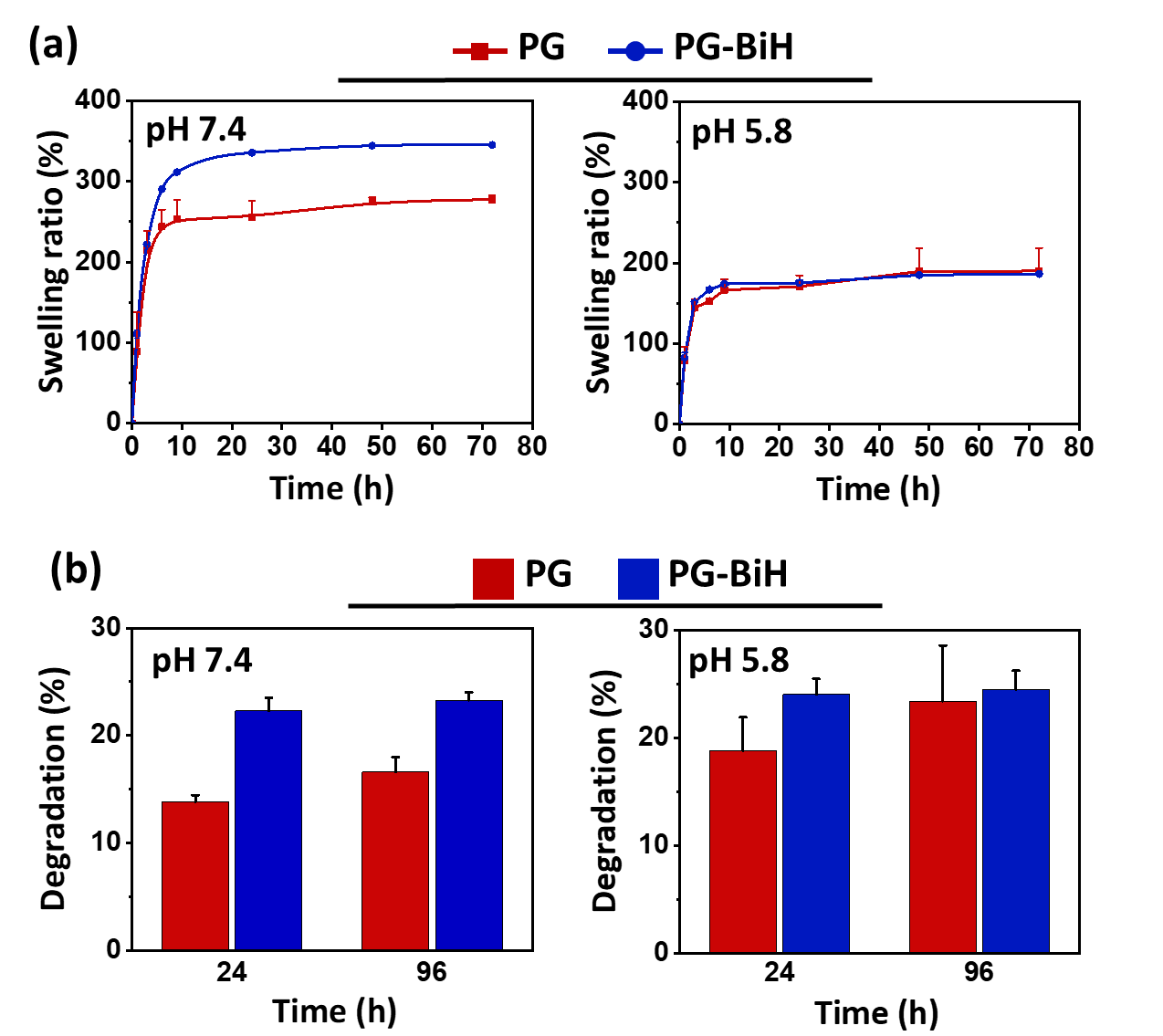
**

**Figure S7.** a) The evaluation of the PG and PG-BiH hydrogelsʼ swelling capacity at pH values of 7.4 and 5.8. b) The degradation percentage of the PG and PG-BiH hydrogels after 24 and 96 h of treatment in PBS at pH values of 7.4 and 5.8. Results are presented as mean±SD (N=3).

**The Initial Water Content and Yield of the PG and PG-BiH Hydrogels**

The initial water content of the PG and PG-BiH hydrogels is almost the same (Figure S8a). In addition, the yield (Figure S8b) of both hydrogels was about 90%. This observation confirmed that the presence of BiH in the hydrogel does not harm the yield (%).

**
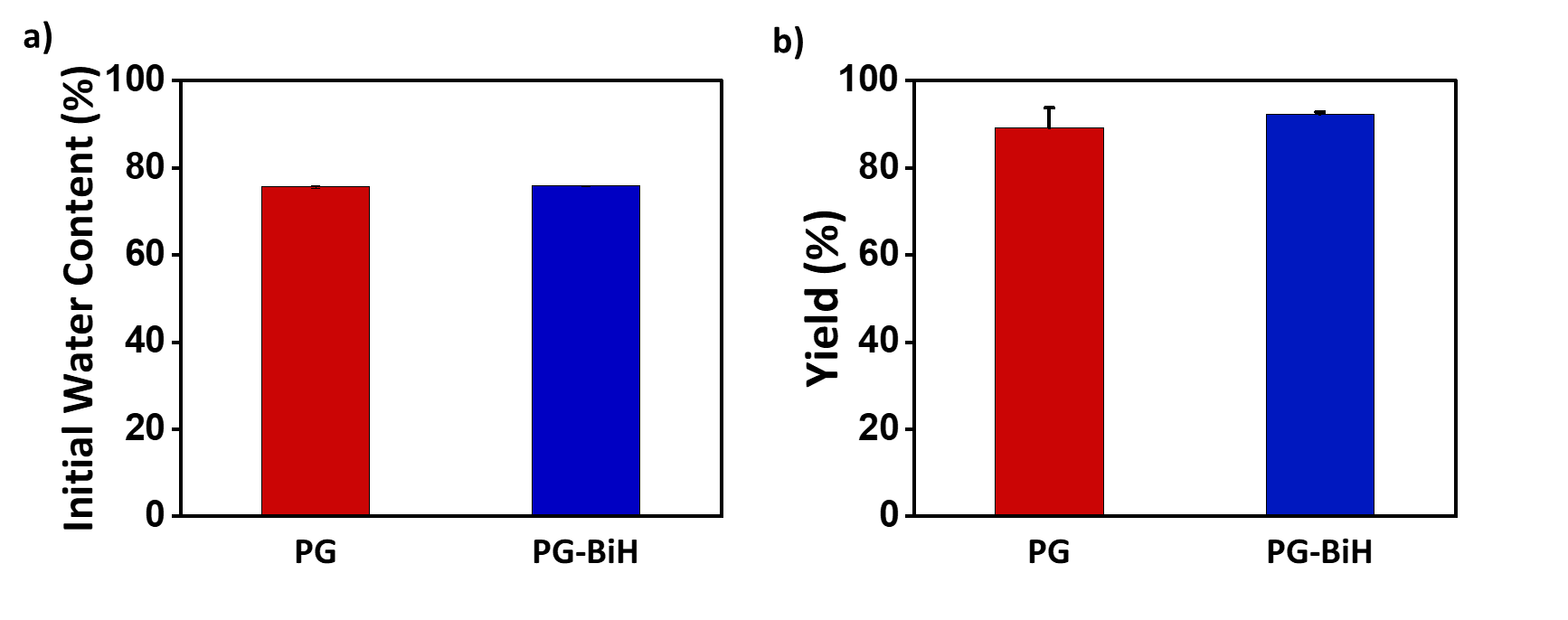
**

**Figure S8.** a) Initial water content and b) the yield of PG and PG-BiH hydrogels. Results are presented as mean±SD (N=3).

**ATR-FTIR Analysis**

Figure S9 presents the ATR-FTIR spectra of pure components and hydrogel. An absorption band at about 1092 cm^-1^ was observed in the spectrum of Bi_2_S_3_ nanorods, which is assigned to the Bi–S vibration modes and approves the formation of Bi_2_S_3_ nanorods [9]. The bands at 1409 and 1010 cm^-1^ are attributed to carboxylate symmetric stretching and C−O−C stretching vibration of HA, and a band at 1627 cm^-1^ is assigned to carboxylate asymmetric stretching vibration of HA [10]. In addition, a band at 1396 cm^-1^ for N-H bending vibration, is one of the characteristics of HA [11]. In the BiH spectrum, the absorption band at 1396 cm^−1^ and 1624 cm^-1^ are attributed to the N-H bending vibration and the C=O stretching vibration of the carboxyl groups of HA, respectively, which demonstrate the successful coating of Bi_2_S_3_ nanorods with HA.

In the spectrum of PEGDGE, the bands at 1091 cm^-1^ and 2867 cm^-1^ are due to the C–O stretching and C-H stretching vibration, respectively [12]. In the spectrum of gelatin, the band at 1639 cm^-1^ is attributed to the presence of C=O stretching vibration of amide I. A band at 1541 cm^-1^ is due to the –NH bending and C-N stretching vibrations of amide II. The 1233 cm^-1^ band is also due to the vibrations in the plane mode of C-N and N-H groups of amide III.[12]

In the spectrum of PMVE-MA, a strong band was observed at 1705 cm^-1^, which can be attributed to –COO– asymmetrical stretching vibrations. The hydroxyl stretching band is observed at 3450 cm^-1^. The bands at 1174 and 1092 cm^-1^ were due to the C–O stretching vibrations and the band at 2923 cm^-1^ can be attributed to C–H stretching vibrations [13].

In the spectrum of PG hydrogel, the band, which is around 1076 cm^-1^ attributed to C–O stretching (ether bond C-O-C) confirms the presence of PEGDGE in the final hydrogel. Moreover, hydroxyl groups –OH constructed during the crosslinking between PEGDGE and gelatin or PMVE-MA are observed at 3459 cm^-1^.


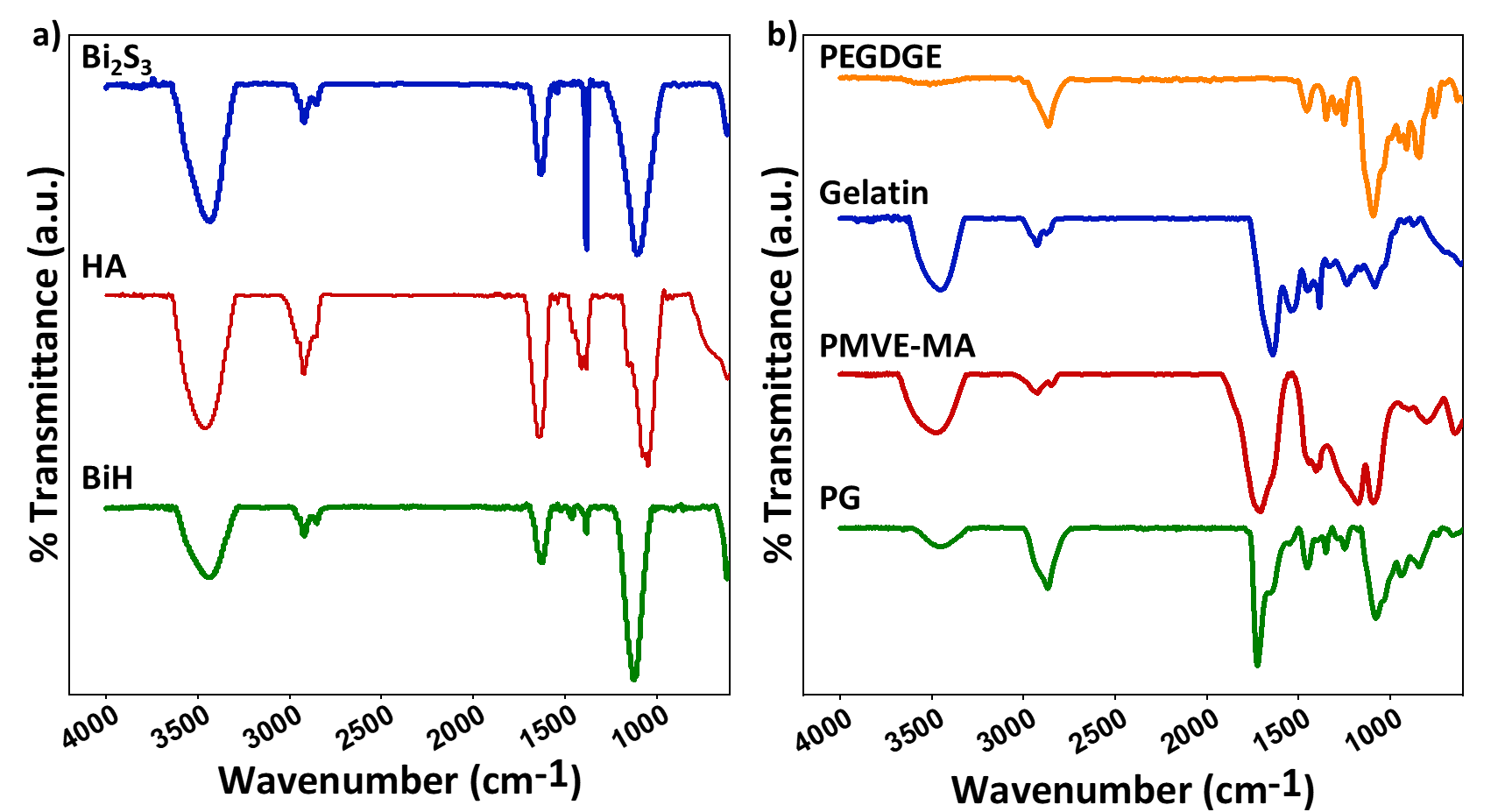


**Figure S9.** ATR-FTIR spectra of a) the Bi_2_S_3_ nanorods, HA and BiH nanorods, as well as b) PG hydrogel and its constructive components.

**XRD Analysis**

The crystallinity of all samples was examined by the X-ray diffraction (XRD) technique (Figure S10). The notable peaks of Bi_2_S_3_ nanorod show its high crystallinity while HA is completely amorphous. In addition, it is shown that HA coating does not affect the crystalline structure of the Bi_2_S_3_ nanorods.

The XRD patterns of gelatin sample revealed an amorphous structure with a broad peak at 2θ~20°. In addition, XRD results of PMVE-MA displayed a broad peak at 2θ~16.5°, suggesting an amorphous structure. In the PG hydrogel, the diffraction peak of PMVE-MA shifted to ~20° due to crosslinking with PEGDGE and the addition of gelatin. Because BiH nanorods were embedded into the network of the PG-BiH hydrogel, its crystal structure is covered and the peaks are not shown.

Furthermore, the peaks of PG-BiH-CCM, and PG-BiH-CCM-SFN hydrogels are almost the same as the PG due to the little amount of CCM, and SFN in the hydrogels.

**Figure S10.** XRD analysis of all components and formulations.

**TGA and DTG Analysis**

Figures S11a and S11b presented the TGA curves and relevant analysis data of all samples under argon atmosphere. There was a slight thermal event (13%) in the Bi_2_S_3_ curve near 200 ^°^C corresponding to the evaporation of absorbed and crystalline water. T10% (temperature for 10% weight loss) of Bi_2_S_3_ was found at 765 °C. The TGA curve of BiH indicated 21% weight loss starting at 200 °C because of the thermal decomposition of HA. T10% of BiH was found at 309 °C. For HA, the first weight loss from 60 °C to 120 °C was due to the evaporation of residually bound water from HA. The second weight loss started at 200 °C, and a char residue of 22.8% remained at 800 °C. The PVME-MA showed a multi-step weight loss curve. The initial degradation step starting at 160 °C corresponded to the dehydration of carboxyl groups, and the final steps displayed the degradation of the copolymer backbone [14]. The thermal degradation of the gelatin started at 250 °C and 80.8% weight loss was observed at 800 °C. The main mass loss of PEGDGE occurred at 160–370 °C and nearly 100% weight loss was observed at 800 °C [15]. All prepared hydrogels exhibited two-step degradation. The initial one started at ~200 °C and was associated with the evaporation of residual moisture and the obvious degradation started at ~310 °C due to the degradation of the plasticizer. In addition, these hydrogels are also composed of both PMVE-MA and gelatin; therefore, they showed the same property with the char residue of 9.4%, 9.6%, and 9.8% in PG, PG-BiH, and PG-BiH-CCM hydrogels respectively.

Furthermore, DTG curves of the hydrogels showed that the maximum decomposition rate occurred in the sharp peak at about 390 °C indicating the improved thermal stability of hydrogels compared to the pure constructive components (Figure S11c).

**
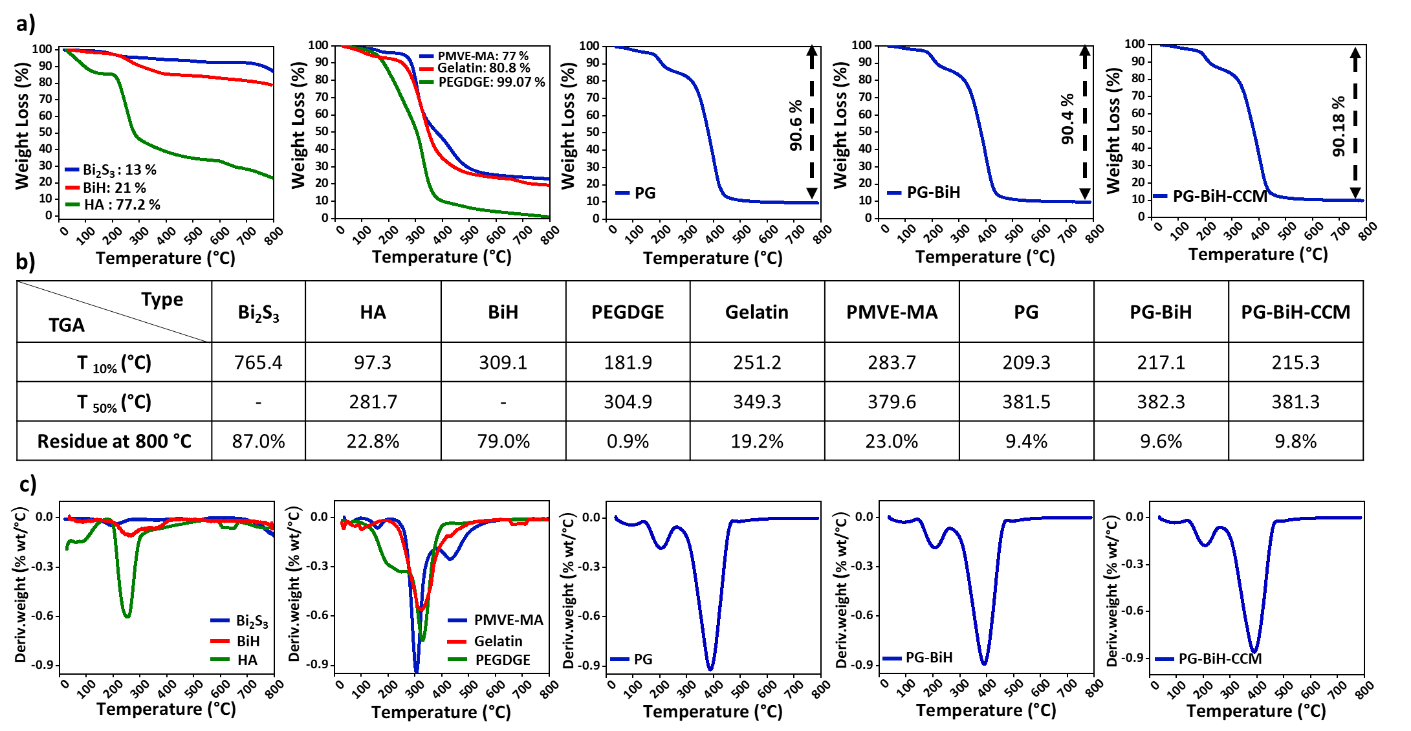
**

**Figure S11.** a) TGA analysis. b) T10% (temperature (°C) at 10% weight loss), T50% (temperature (°C) at 50% weight loss), and residual content at 800 °C retrieved from TGA. c) DTG analysis.


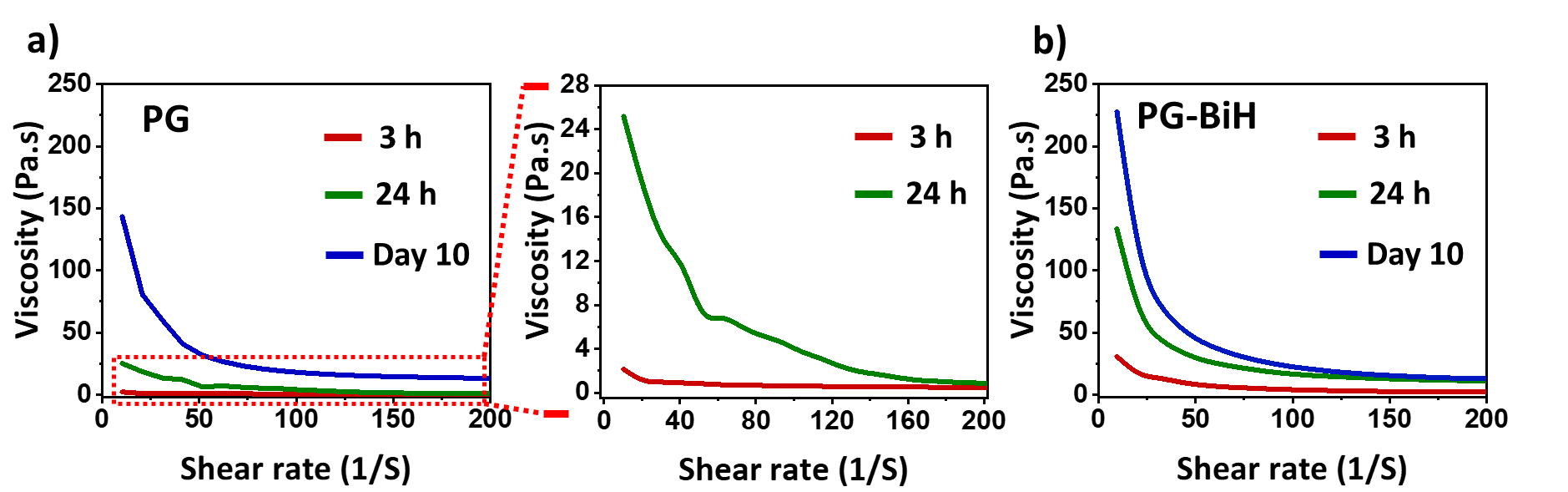


**Figure S12.** Viscosity of a) PG and b) PG-BiH hydrogels at 25 °C as a function of shear rate at 3 h, 24 h, and 10 days after hydrogel formation. Both hydrogels represented a shear thinning behavior. The results showed the time-dependent increment in the viscosity of both PG and PG-BiH hydrogels. Moreover, the PG-BiH hydrogel showed higher viscosity in comparison to PG due to the incorporation of BiH nanorods in the hydrogel.

.
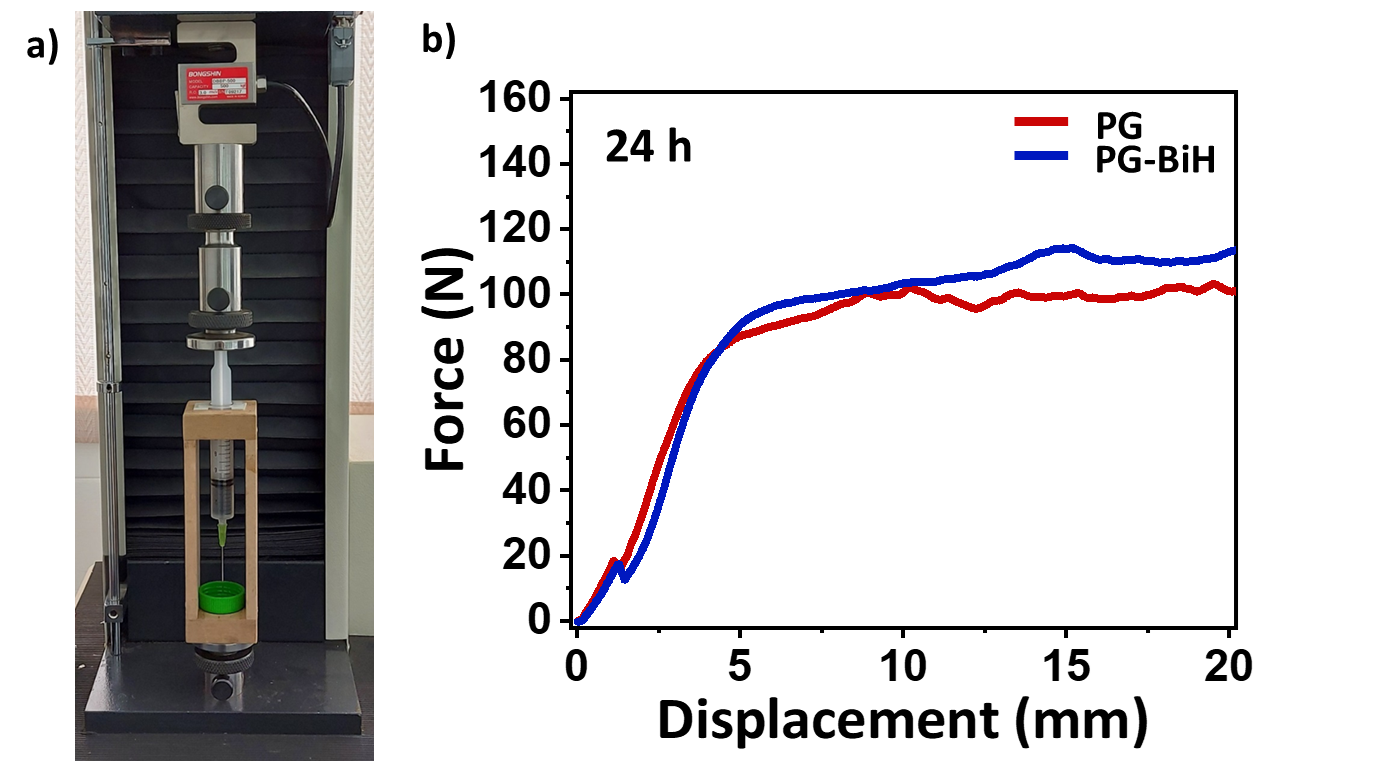


**Figure S13.** Injectability evaluation of hydrogels 24 h post-fabrication at room temperature using a 21 G needle. a) Set up of the injectability assay. b) Injectability force versus displacement curve for PG and PG-BiH hydrogels injected from a 10-ml syringe with 21 G needle. The hydrogels were fabricated and kept for 24 h at room temperature before starting the test, and the results showed that the needed force for injection was increased time-dependently compared to 3 h and 12 h incubated samples.

*
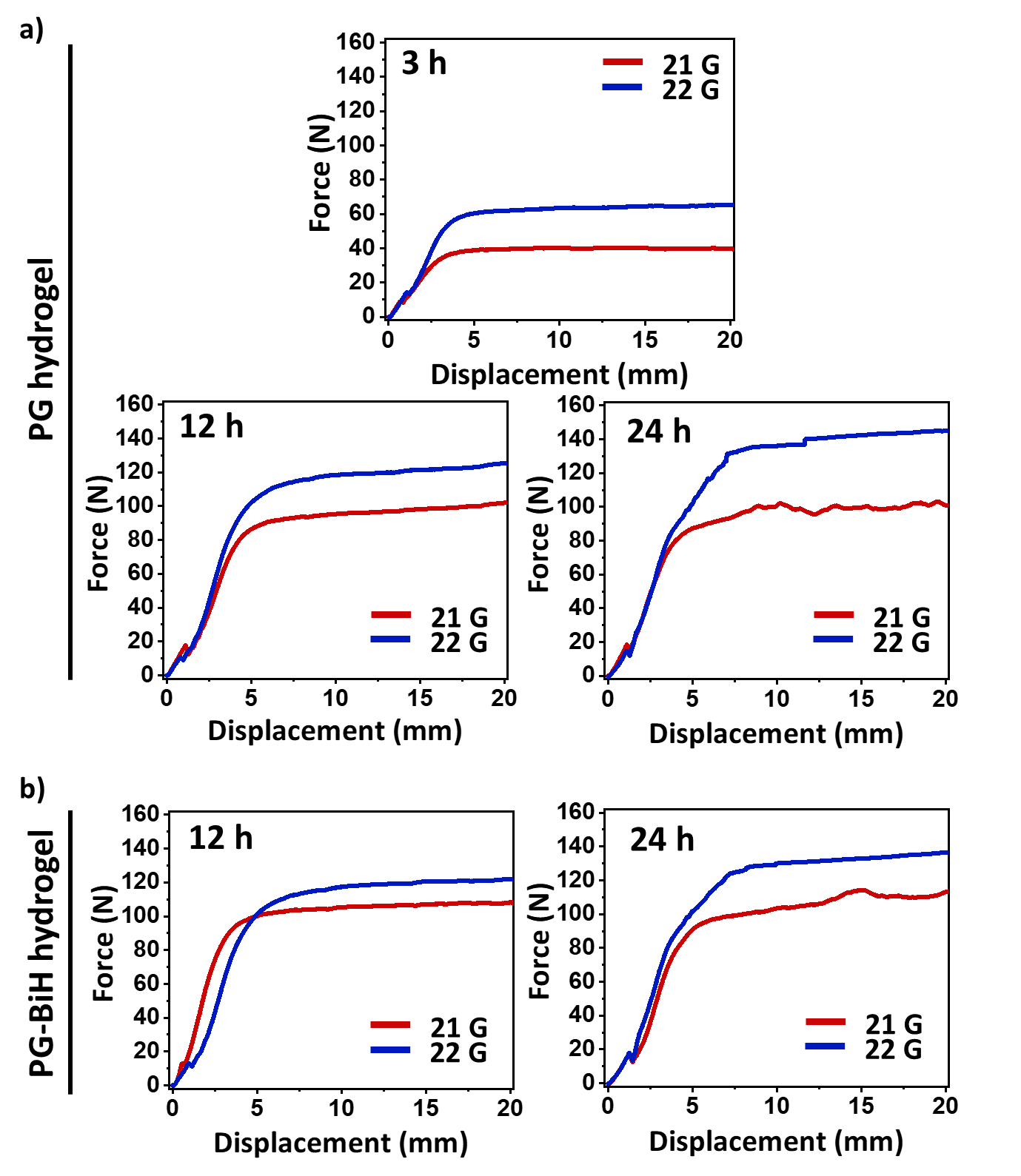
*

**Figure S14.** Injectability curves show the injection force required for the injection of a) PG and b) PG-BiH hydrogels using a 10-ml syringe with 21 G and 22 G needles at different time points after fabrication and keeping at room temperature. The force needed to extrude the hydrogel from the syringe increases as the diameter of the needle decreases. Moreover, the injection force was increased over time in comparison to the needed force for injection at 3 h after the fabrication of hydrogels. This is due to the time-dependent increment in chemical crosslinking in the hydrogelsʼ network and enhanced viscosity.


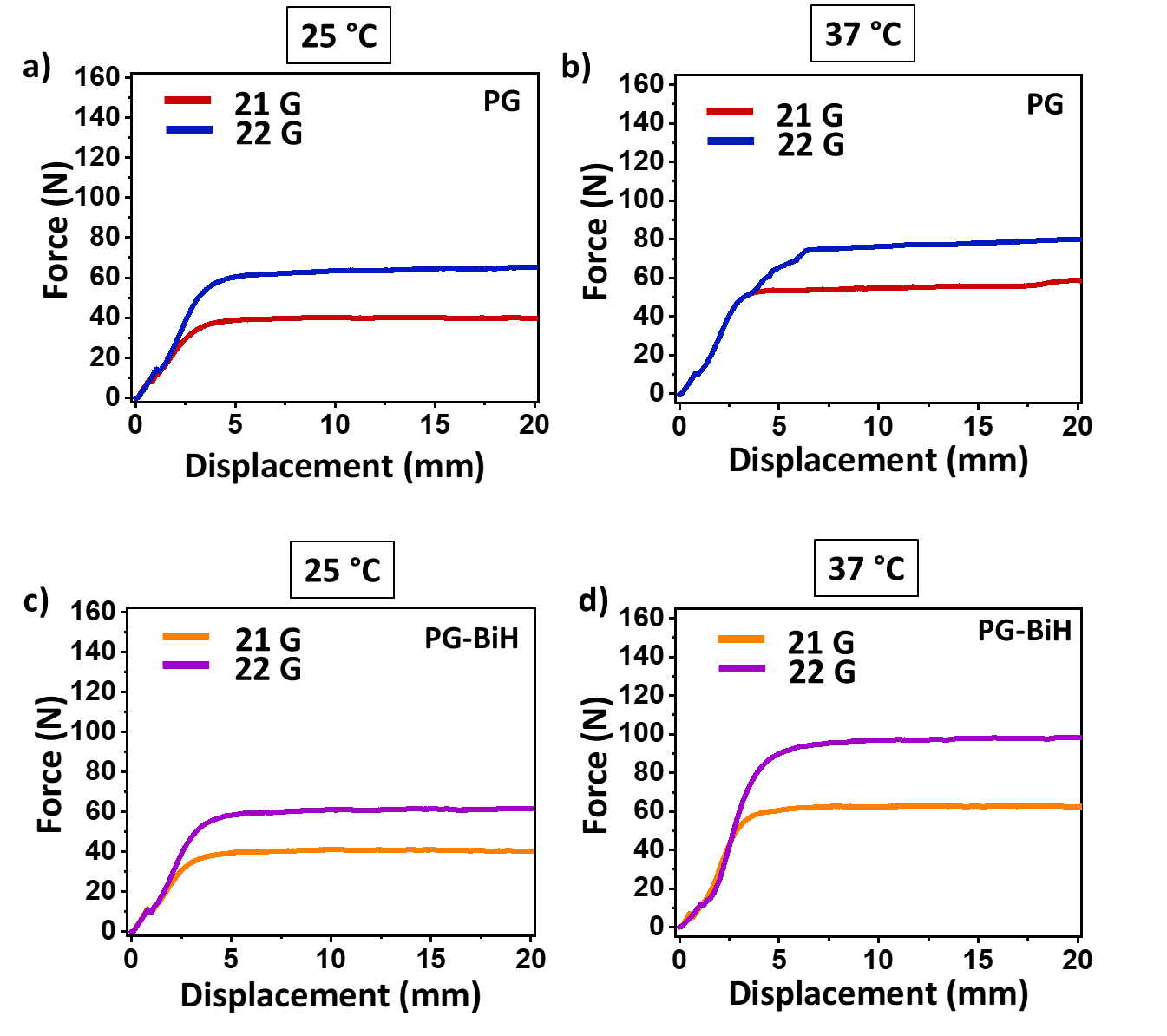


**Figure S15.** Temperature dependent injectability behavior of the hydrogels. Injectability curves demonstrate the needed force for b) PG and d) PG-BiH to be injected from a 10-ml syringe with 21 G and 22 G needles when the hydrogels were incubated at 37 °C for 3 h post-fabrication. a,c) were repeated in order to better understanding of the effect of temperature on the force required for injection. The force needed for the ejection of PG hydrogel after 3 h incubation in 37 °C increased about 14.6 N and 12.7 N using 21 G and 22 G needles, respectively, at 10 mm displacement compared to the 3 h incubation in room temperature. Moreover, the increased amount of force for PG-BiH hydrogel was 21 N and 35.8 N using 21 G and 22 G needles, respectively at 10 mm displacement in comparison to incubation in room temperature, which can be due to higher crosslinking reactions assisted by the increased temperature.


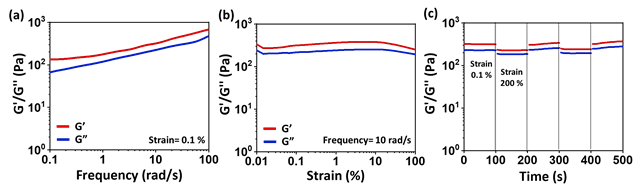
**Figure S16.** Rheological characterizations of the PG-BiH hydrogels 24 h after preparation. a) Frequency-dependent (strain fixed as 0.1%) and b) strain-dependent (frequency fixed as 10 rad s^-1^) oscillatory shear rheology of the hydrogels. c) Continuous step-strain measurements by the substitution of strain between 0.1 and 200%, indicating the self-healing property of the PG-BiH hydrogel. The PG-BiH hydrogel showed higher G′ and G″ values on day 10 compared to 24 h post-fabrication of hydrogels, which can be ascribed to the time-dependent increase of chemical crosslinking of polymers in the hydrogel.

**
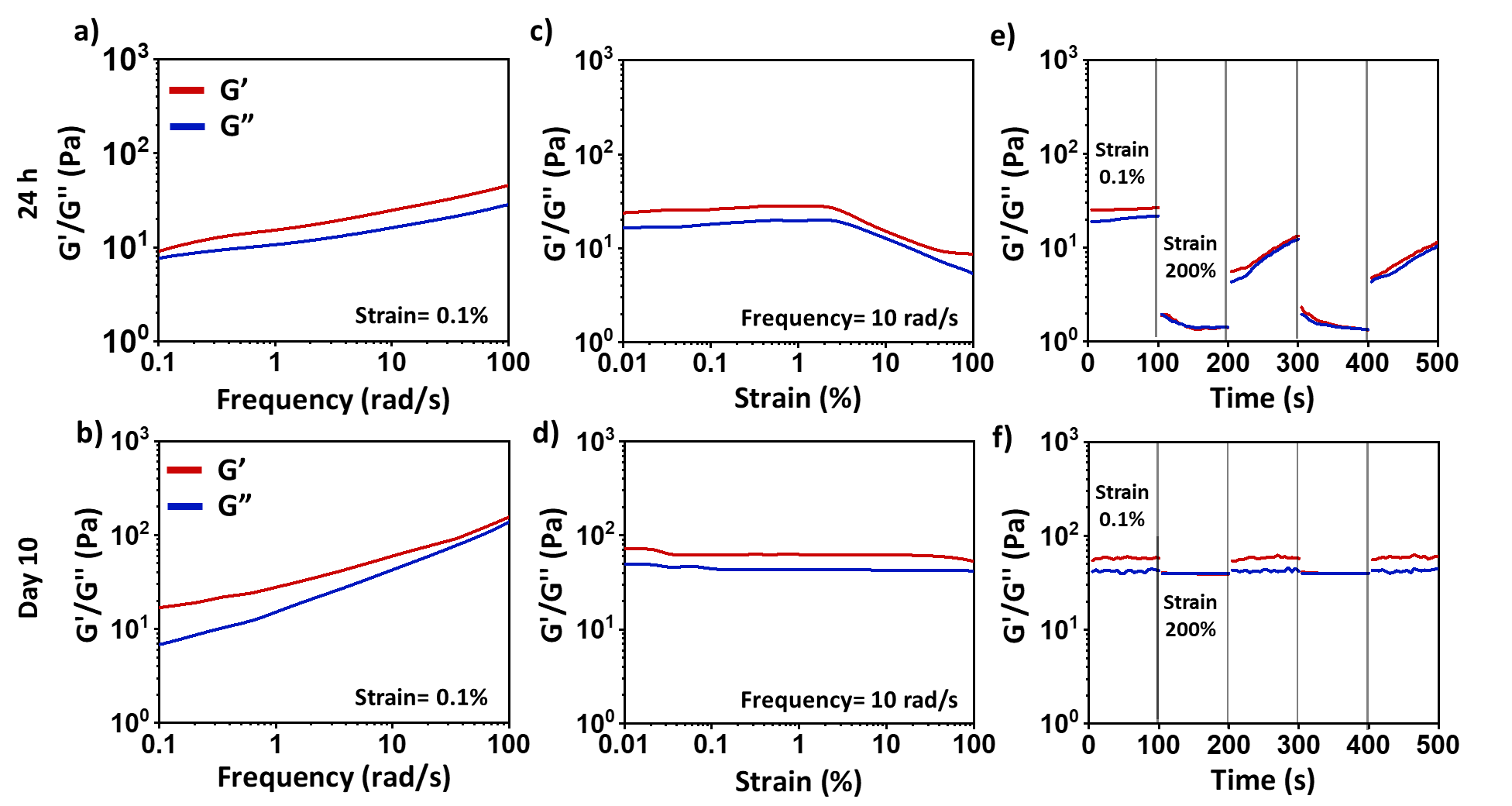
**

**Figure S17.** Rheological characterizations of the PG hydrogel 24 h and 10 days after preparation. a, b) Frequency sweep (strain fixed as 0.1%), which demonstrates gel-like behavior of hydrogel due to the higher G′ values than G″ values. c,d) Strain-dependent (frequency fixed as 10 rad s^-1^) oscillatory shear rheology of the hydrogels. The moduli of PG were independent of strain amplitude at low strain ranging from 0.01 to 2% at 24 h post-fabrication, as well as 0.01 to 100% on day 10 after fabrication. e,f) Continuous step-strain measurements by substitution of strain between 0.1 and 200%, indicating the self-healing property of PG hydrogel after 24 h and 10 days after preparation, respectively.


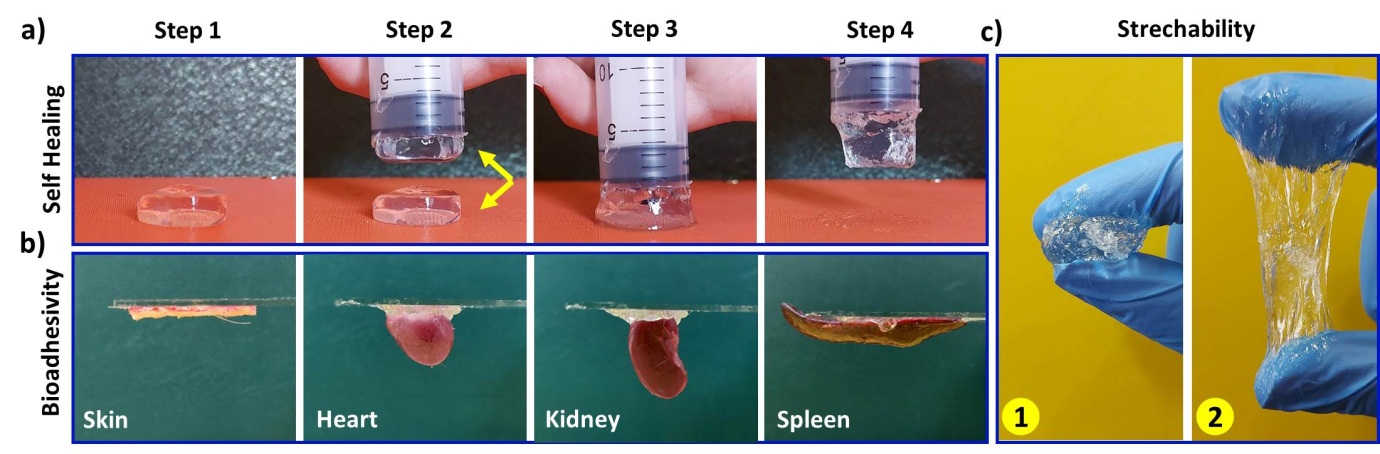


**Figure S18.** a) Self-healing, b) bioadhesivity, and c) stretchability of PG hydrogel 10 days after hydrogel formation.


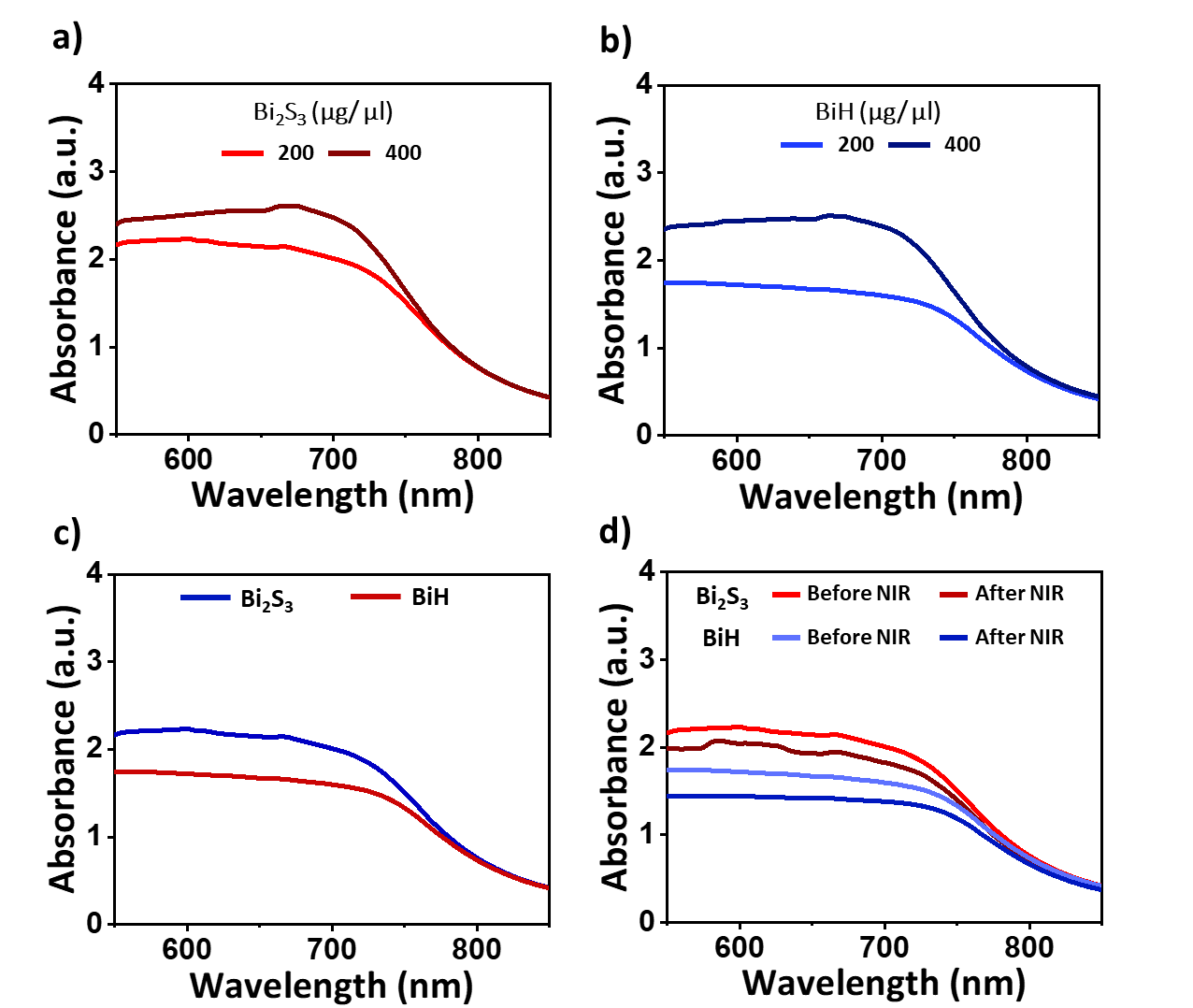


**Figure S19.** a,b) UV–vis absorption spectra of Bi_2_S_3_ and BiH nanorods with different concentrations. c) UV–vis absorption spectra of Bi_2_S_3_ and BiH nanorods (200 μg ml^-1^). d) UV-vis absorption spectra of Bi_2_S_3_ and BiH nanorods before and after irradiation (10 min, 1.5 W cm^-2^). Both Bi_2_S_3_ and BiH nanorods possess absorption in 808 nm, which can be used to mediate PTT. Moreover, the NIR irradiation (10 min, 1.5 W cm^-2^, 808 nm) did not cause any effect on the UV-vis absorption rate of both nanorods at 808 nm.


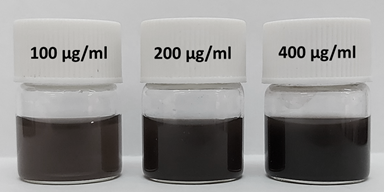


**Figure S20.** The digital photograph of 100, 200, and 400 μg ml^-1^ of BiH nanorods solution. The photo is taken 1 h after the synthesis of nanorods. The intensity of black color, which is characteristic of Bi_2_S_3_ nanorods, increased by increment in BiH concentration.

**
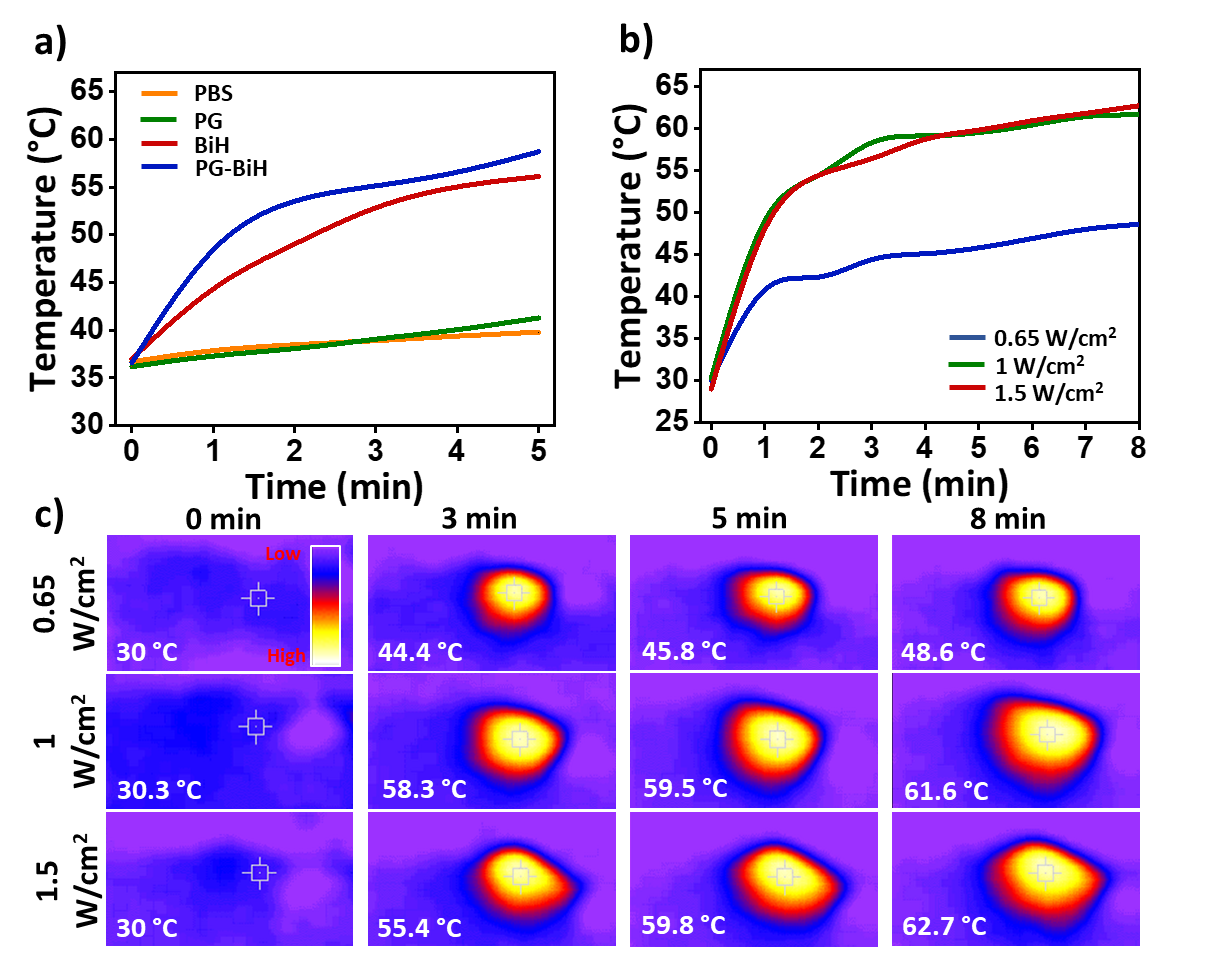
Figure S21.** In vivo assessment of the NIR-mediated heat generation by the nanoparticles and the hydrogel. a) Body temperature of the mice injected with 100 μl of PBS, PG, BiH nanorods (200 μg ml^-1^), and PG-BiH over 5 min of NIR irradiation (808 nm; 1.5 W cm^-2^). b) Body temperature of the mice injected with PG-BiH hydrogel (100 μl) under NIR laser irradiation with different power densities (0.65, 1, and 1.5 W cm^-2^) for 8 min. c) Representative IR thermal images of the mice after injection with PG-BiH upon NIR irradiation (0.65, 1, and 1.5 W cm^-2^) for 8 min.

**Cell Viability Studies of Bi_2_S_3_ Nanorods**

**
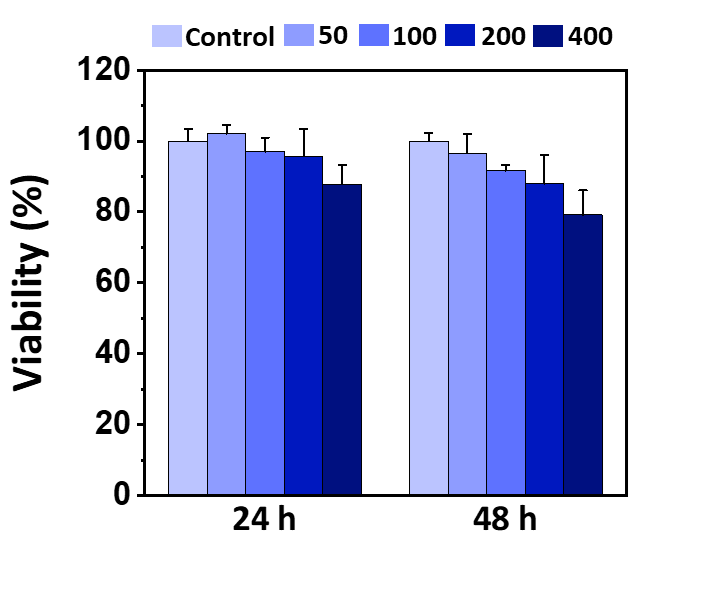
**

**Figure S22.** Cell viability of untreated 4T1 cells as the control group and the cells treated with different concentrations of Bi_2_S_3_ (50, 100, 200, and 400 μg ml^-1^) for 24 h and 48 h. The results were plotted as mean±SD (N=3).


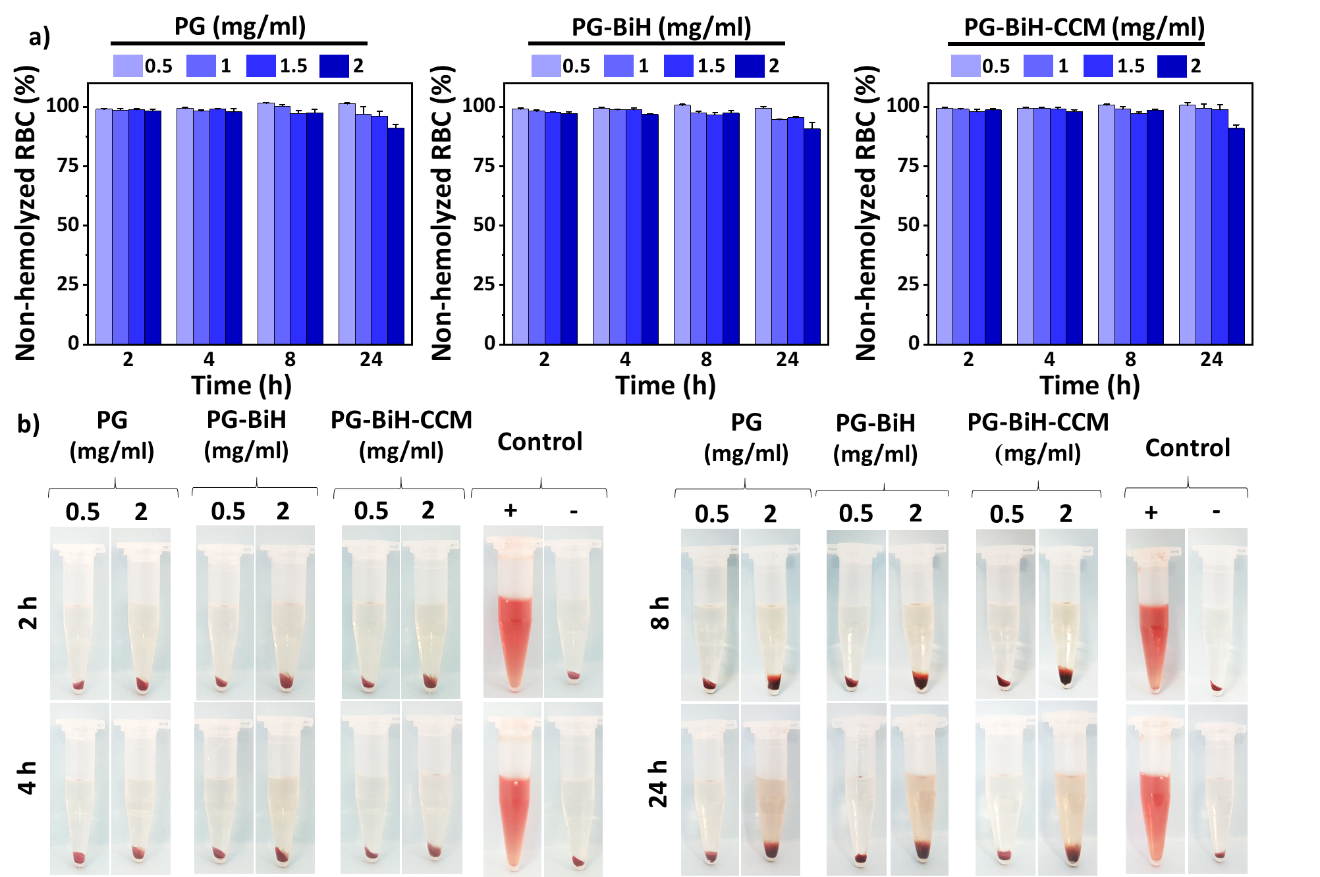


**Figure S23.** a) The percentage of non-hemolyzed RBCs after treatment with different concentrations of hydrogels as a function of time. The results were plotted as mean±SD (N=3). b) The photos of centrifuged samples at different time points and concentrations. All groups showed nearly a transparent supernatant similar to the RBCs treated with PBS (pH 7.4) as the negative control, while the DW lysed all RBCs and created a red color in the supernatant (positive control).


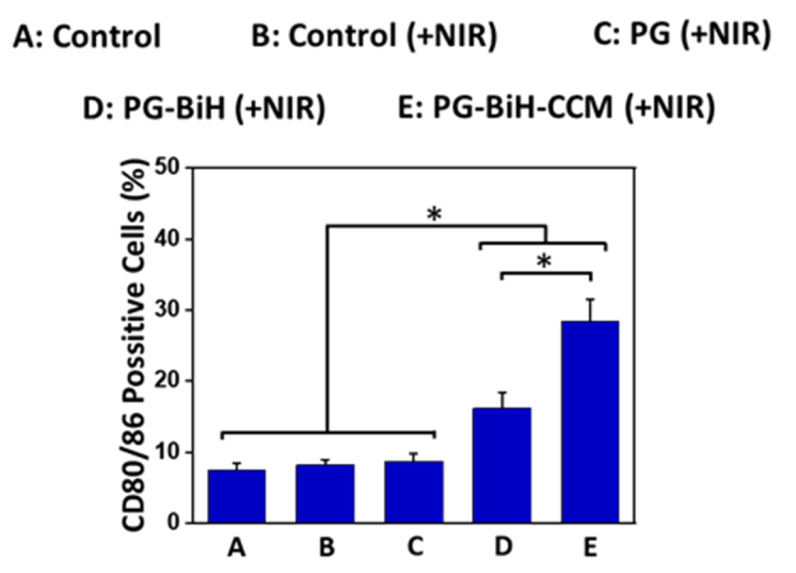


**Figure S24.** Quantification of the expression levels of CD80 and CD86 on the BMDCs after treatment under different conditions. The results are plotted as mean±SD (N=3), (*p<0.05).


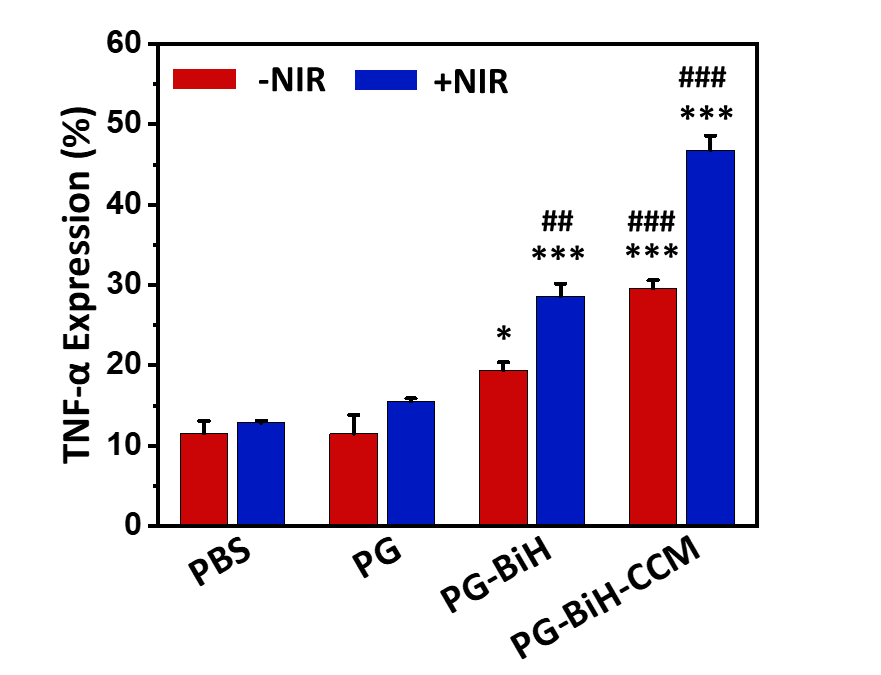


**Figure S25.** The percentage of TNF-α expression obtained from microscopy images of tumor tissues of different groups treated with or without NIR irradiation. Results are presented as mean±SD (N=3). The statistical analysis was performed using One-way ANOVA, by comparing each group with the PBS without NIR (*p<0.05. ** p<0.01 and *** p<0.001) and with NIR (# p<0.05. ## p<0.01 and ### p<0.001).

**
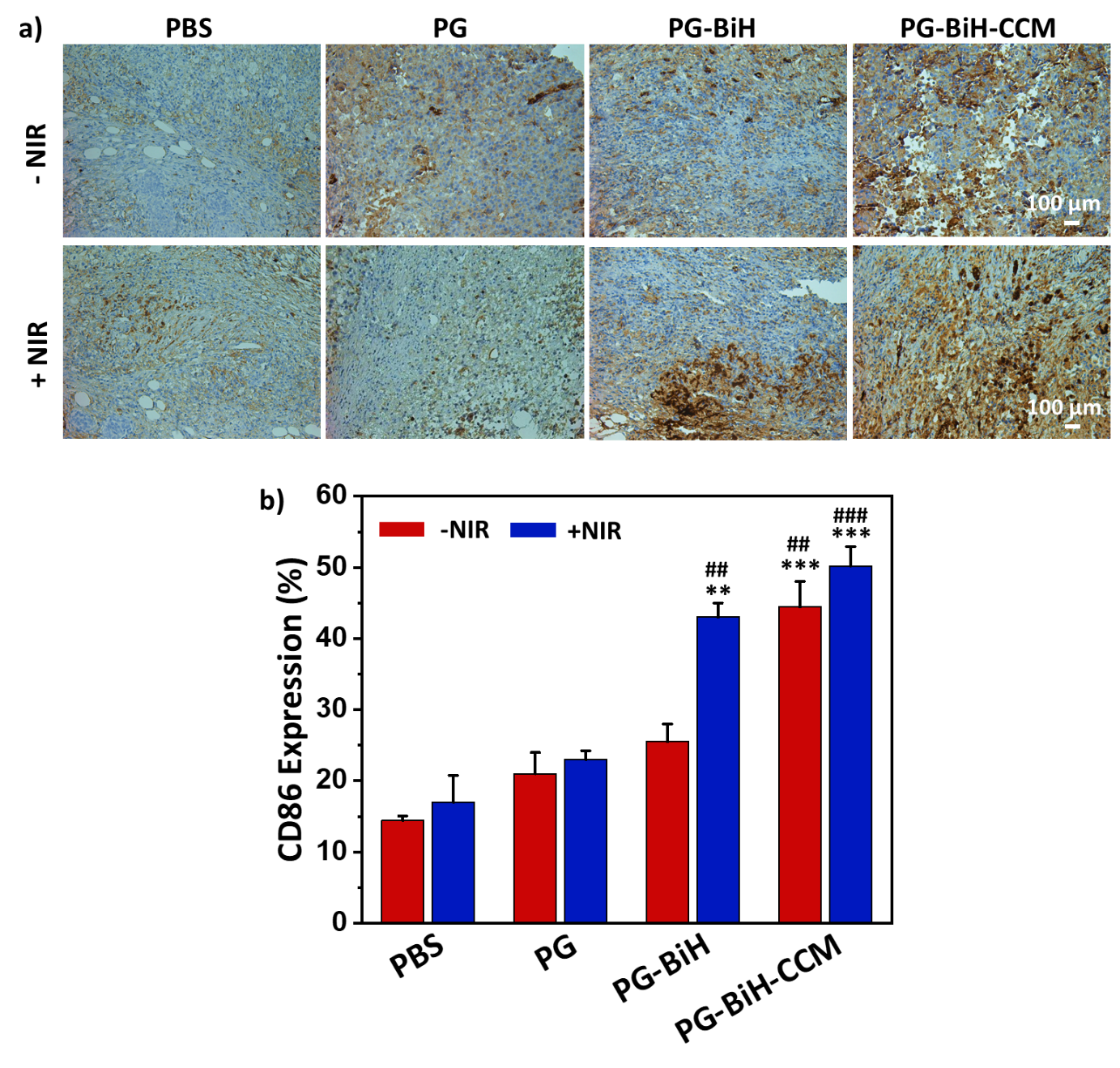
**

**Figure S26. a)** Representative photographs of CD86 expression in tumor tissues of mice treated with different formulations after 15 days. b) The percentage of CD86 expression obtained from microscopy images of tumor tissues of different groups treated with or without NIR irradiation. Results are presented as mean±SD (N=3). The statistical analysis was performed using One-way ANOVA, by comparing each group with the PBS without NIR (*p<0.05. ** p<0.01 and *** p<0.001) and with NIR (# p<0.05. ## p<0.01 and ### p<0.001).


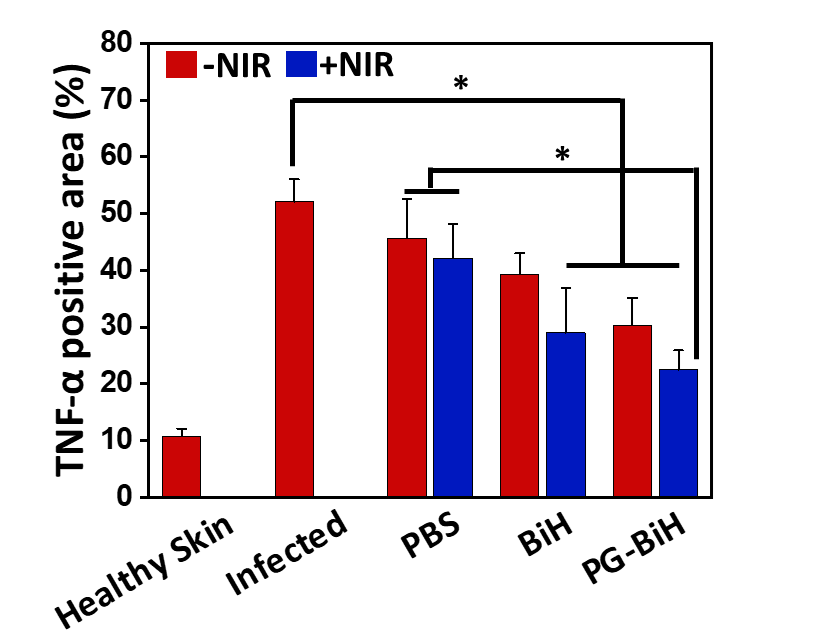


**Figure S27.** The percentage of TNF-α positive area of obtained microscopy images from different groups treated with or without NIR irradiation in a skin abscess model. Results are presented as mean±SD (N=3). The statistical analysis was performed using One-way ANOVA (* demonstrates differences with a significance level of p<0.05), by comparing each group with the infected group (without any treatment) and PBS-treated groups.


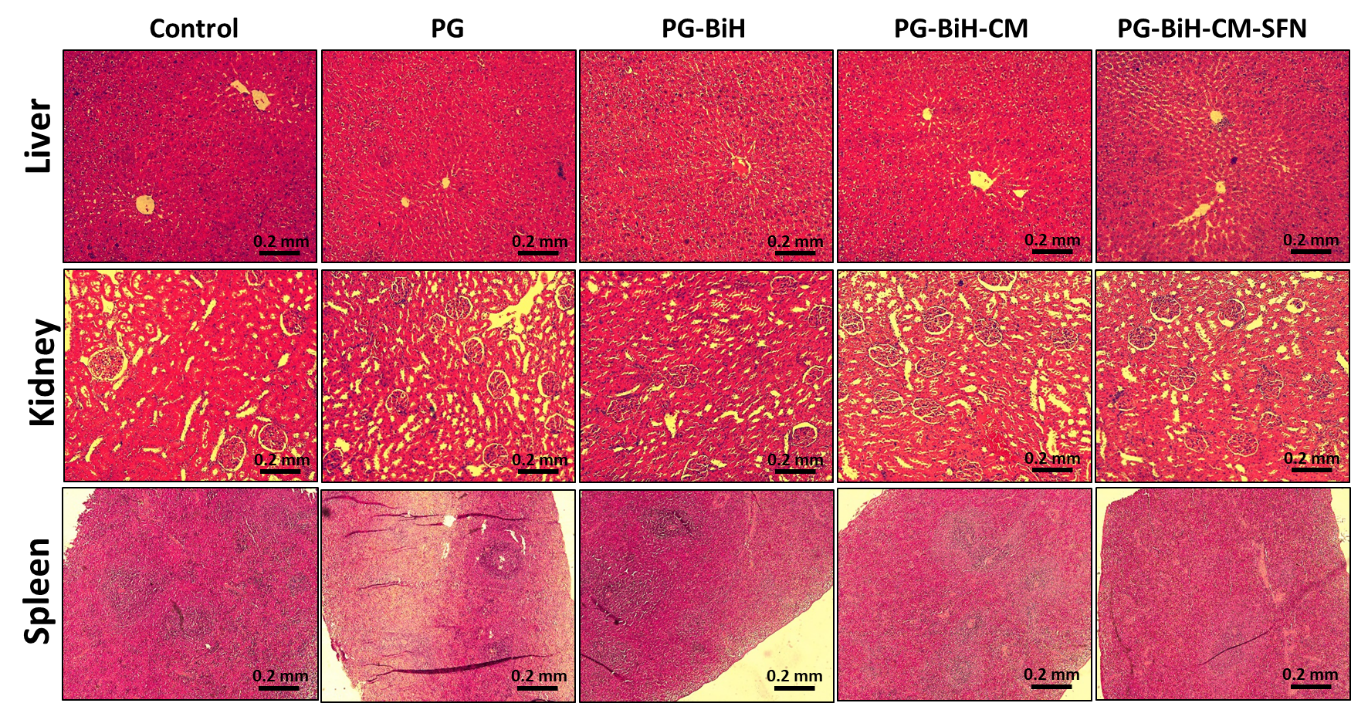


**Figure S28.** H&E stained liver, kidney, and spleen 14 days after injection of PG, PG-BiH, PG-BiH-CCM and PG-BiH-CCM-SFN hydrogels (1 ml) at the dorsal region of rats.

**
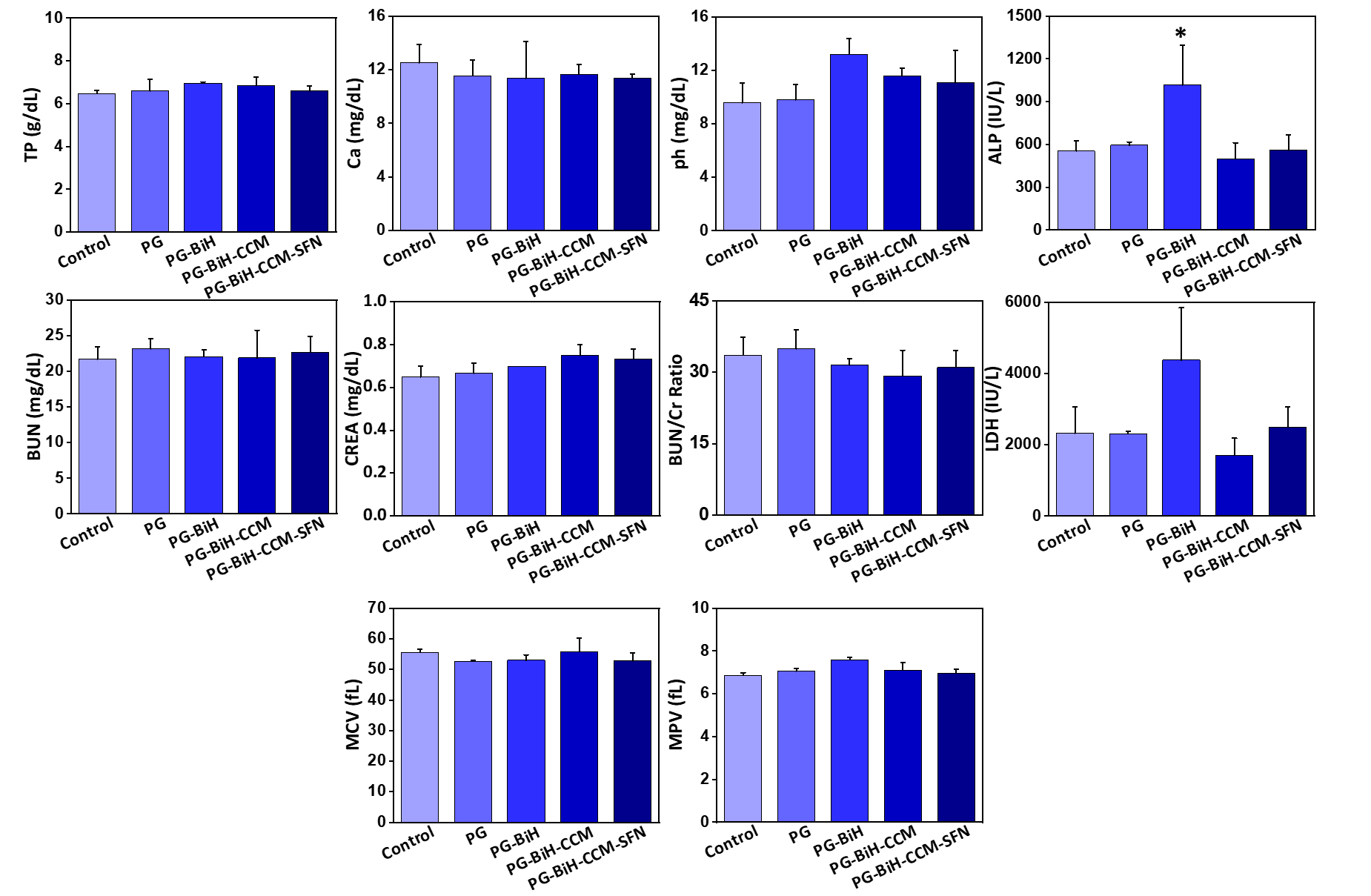
**

**Figure S29.** Biochemical and hematological factors of animals 14 days after treatment with different types of hydrogels. Results are presented as mean±SD (N=4). The statistical analysis was performed using One-way ANOVA (*p<0.05 *vs*. control group).


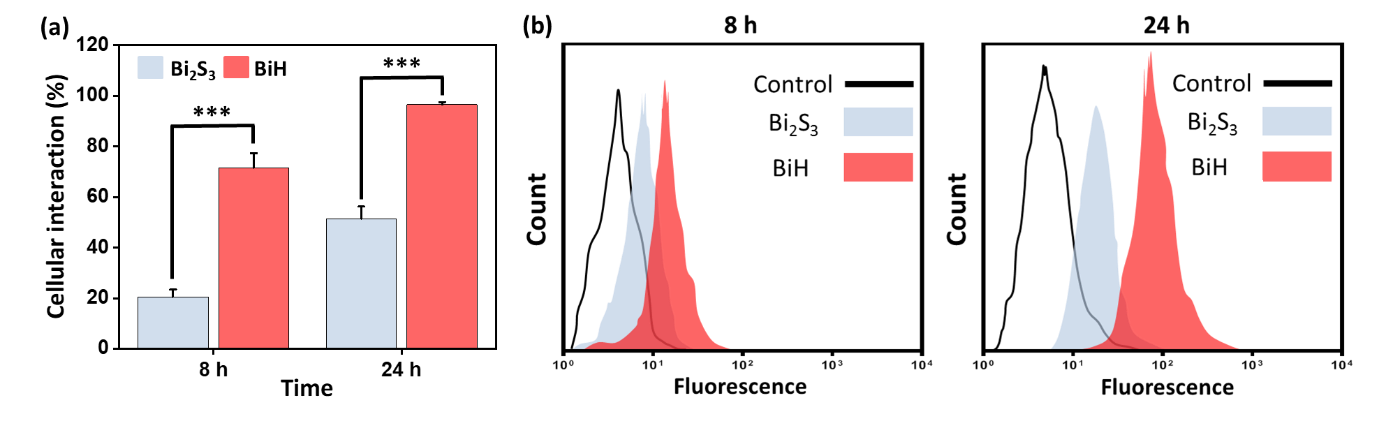


**Figure S30.** (a) Cellular interaction of Bi_2_S_3_ and BiH nanorods with the breast cancer cells after 8 h and 24 h. Data are presented as mean±SD (N=3), (***p<0.001). (b) Representative fluorescence intensity of control, Bi_2_S_3_ and BiH nanorods after 8 h and 24 h incubation with the cancer cells.


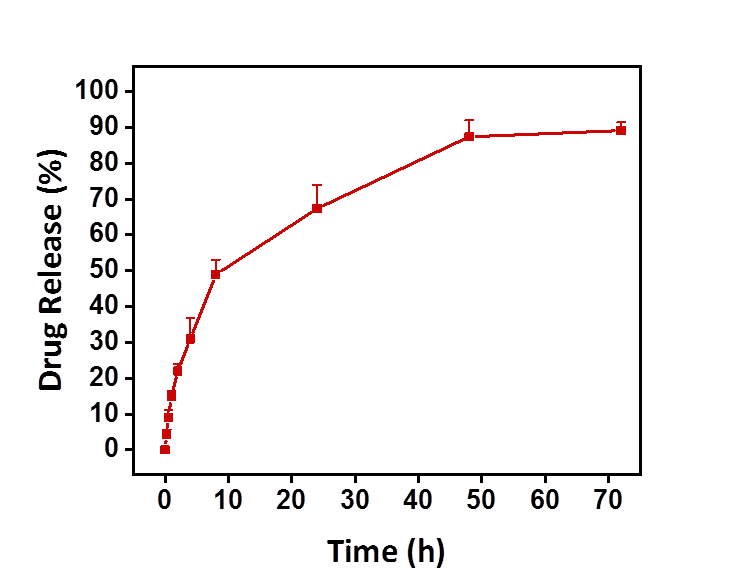


**Figure S31.** Drug release graph showing cumulative SFN release (%) from the final hydrogel during 72 h. Data are presented as mean±SD (N=3). The sustained release of the drug can provide an effective anti-cancer environment in the tumor tissue.


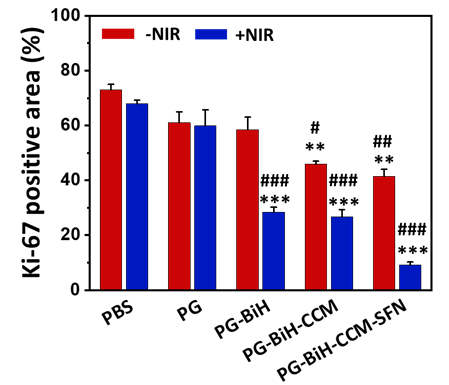


**Figure S32.** The percentage of Ki-67 positive area in the obtained microscopy images from different groups treated with or without NIR irradiation. Results are presented as mean±SD (N=3). The statistical analysis was performed using One-way ANOVA, by comparing each group with the PBS without NIR (*p<0.05. ** p<0.01 and *** p<0.001) and with NIR (# p<0.05. ## p<0.01 and ### p<0.001).

**References**

[1] Y. Ramos Reynoso, A. Martinez-Ayala, M. Pal, F. Paraguay-Delgado, N. R. Mathews, Bi2S3 nanoparticles by facile chemical synthesis: Role of pH on growth and physical properties, Adv. Powder. Technol. 29 (2018) 3561-3568. <https://doi.org/10.1016/j.apt.2018.09.037>.

[2] J. Li, D. Huang, R. Cheng, P. Figueiredo, F. Fontana, A. Correia, S. Wang, Z. Liu, M. Kemell, G. Torrieri, E. M. Mäkilä, J. J. Salonen, J. Hirvonen, Y. Gao, J. Li, Z. Luo, H. A. Santos, B. Xia, Multifunctional Biomimetic Nanovaccines Based on Photothermal and Weak-Immunostimulatory Nanoparticulate Cores for the Immunotherapy of Solid Tumors, Adv. Mater. 34 (2022) 2108012. <https://doi.org/10.1002/adma.202108012>.

[3] Z. Ahmadian, A. Correia, M. Hasany, P. Figueiredo, F. Dobakhti, M. R. Eskandari, S. H. Hosseini, R. Abiri, S. Khorshid, J. Hirvonen, H. A. Santos, M.-A. Shahbazi, A Hydrogen-Bonded Extracellular Matrix-Mimicking Bactericidal Hydrogel with Radical Scavenging and Hemostatic Function for pH-Responsive Wound Healing Acceleration, Adv. Healthc. Mater. 10 (2021) 2001122. <https://doi.org/10.1002/adhm.202001122>.

[4] T. E. Robinson, E. A. B. Hughes, N. M. Eisenstein, L. M. Grover, S. C. Cox, The Quantification of Injectability by Mechanical Testing, J. Vis. Exp. (2020) <https://doi.org/10.3791/61417>.

[5] F. Wu, L. Chen, L. Yue, K. Wang, K. Cheng, J. Chen, X. Luo, T. Zhang, Small-Molecule Porphyrin-Based Organic Nanoparticles with Remarkable Photothermal Conversion Efficiency for in Vivo Photoacoustic Imaging and Photothermal Therapy, ACS Appl. Mater. Interfaces 11 (2019) 21408-21416. <https://doi.org/10.1021/acsami.9b06866>.

[6] L.-X. Yan, L.-J. Chen, X. Zhao, X.-P. Yan, pH Switchable Nanoplatform for In Vivo Persistent Luminescence Imaging and Precise Photothermal Therapy of Bacterial Infection, Adv. Funct. Mater. 30 (2020) 1909042. <https://doi.org/10.1002/adfm.201909042>.

[7] F. M. Carbinatto, A. D. de Castro, R. C. Evangelista, B. S. F. Cury, Insights into the swelling process and drug release mechanisms from cross-linked pectin/high amylose starch matrices, Asian J. Pharm. Sci. 9 (2014) 27-34. <https://doi.org/10.1016/j.ajps.2013.12.002>.

[8] S. Trombino, T. Ferrarelli, M. Pellegrino, E. Ricchio, L. Mauro, S. Andò, N. Picci, R. Cassano, Anticancer activity of a hydrogel containing folic acid towards MCF-7 and MDA-MB-231 cells, Anticancer Res. 33 (2013) 4847-4854.

[9] H. Li, F. Deng, Y. Zheng, L. Hua, C. Qu, X. Luo, Visible-light-driven Z-scheme rGO/Bi2S3–BiOBr heterojunctions with tunable exposed BiOBr (102) facets for efficient synchronous photocatalytic degradation of 2-nitrophenol and Cr(vi) reduction, Environ. Sci. Nano 6 (2019) 3670-3683. <https://doi.org/10.1039/C9EN00957D>.

[10] B.-P. Jiang, L. Zhang, Y. Zhu, X.-C. Shen, S.-C. Ji, X.-Y. Tan, L. Cheng, H. Liang, Water-soluble hyaluronic acid–hybridized polyaniline nanoparticles for effectively targeted photothermal therapy, J. Mater. Chem. B 3 (2015) 3767-3776. <https://doi.org/10.1039/C4TB01738B>.

[11] S. Cordeiro, B. Silva, A. M. Martins, H. M. Ribeiro, L. Gonçalves, J. Marto, Antioxidant-Loaded Mucoadhesive Nanoparticles for Eye Drug Delivery: A New Strategy to Reduce Oxidative Stress, Processes 9 (2021) 379. <https://doi.org/10.3390/pr9020379>.

[12] K. Dey, S. Agnelli, M. Serzanti, P. Ginestra, G. Scarì, P. Dell'Era, L. Sartore, Preparation and properties of high performance gelatin-based hydrogels with chitosan or hydroxyethyl cellulose for tissue engineering applications, Int. J. Polym. Mater. Polym. Biomater. 68 (2018) 1-10. <https://doi.org/10.1080/00914037.2018.1429439>.

[13] Z. Wang, W. Xu, H. Peng, X. Tang, Polyanion modulated evolution of perovskite BiFeO3 microspheres to microcubes by a microwave assisted hydrothermal method, J. Mater. Res. 28 (2013) 1498-1504. <https://doi.org/10.1557/jmr.2013.130>.

[14] A. V. Torres-Figueroa, C. J. Pérez-Martínez, J. C. Encinas, S. Burruel-Ibarra, M. I. Silvas-García, A. M. García Alegría, T. Del Castillo-Castro, Thermosensitive Bioadhesive Hydrogels Based on Poly(N-isopropylacrilamide) and Poly(methyl vinyl ether-alt-maleic anhydride) for the Controlled Release of Metronidazole in the Vaginal Environment, Pharmaceutics 13 (2021) 1284. <https://doi.org/10.3390/pharmaceutics13081284>.

[15] B. Oktay, Poly(ethylene glycol)/Polyvinyl chloride Composite Form‐Stable Phase‐Change Materials by the Azide‐Alkyne Click Reaction, ChemistrySelect 3 (2018) 11737-11743. <https://doi.org/10.1002/slct.201802604>.
